# Palladium Catalyzed Allylic C-H Oxidation Enabled by Bicyclic Sulfoxide Ligands

**DOI:** 10.3390/org4020023

**Published:** 2023-06-13

**Authors:** Yuming Wen, Jianfeng Zheng, Alex H. Evans, Qiang Zhang

**Affiliations:** Department of Chemistry, University at Albany, State University of New York, Albany, NY 12222, USA

**Keywords:** C-H activation, bicyclic ligand, sulfoxide, palladium, amino acids

## Abstract

The activation of C-H bonds is a potent tool for modifying molecular structures in chemistry. This article details the steps involved in a novel ligand bearing a bicyclic [3.3.1]-nonane framework and bissulfoxide moiety. A palladium catalyzed allylic C-H oxidation method enables a direct benzyl-allylic functionalization with the bissulfoxide ligand. Bissulfoixde ligand possesses a rapidly constructed bicyclic [3.3.1] framework and it proved to be effective for enabling both N-and C-alkylation. A total of 13 C-H activation productions were reported with good to excellent yields. This report validated that it is necessary to include bissulfoxide as a ligand for superior reactivities. Naftifine was produced utilizing developed C-H functionalization methodology in good overall yields.

## 1. Introduction

The field of organic chemistry has witnessed significant advancements, allowing for the achievement of challenging covalent bond formations. In recent years, the area of interest revolving around the direct activation of inert carbon-hydrogen (C-H) bonds has drawn substantial attention. This powerful strategy (C-H activation) enables the transformation of these traditionally unreactive bonds into diverse functional groups, thereby facilitating molecular structure modifications with broad applications across various domains of chemistry. C-H bonds are a ubiquitous feature of many molecules and are often considered to be unreactive. In particular, C(sp3)–H bonds are notoriously difficult to activate compared to other types of C-H bonds. However, C(sp3)–H bonds located in the allylic position are comparatively more reactive. Remarkable strides have been made in this area of research, including the identification of effective metal and ligand complexes for C-H activation. In terms of catalyst selection, it is clear that Pd salts are a rather cost-effective choice with wide accessibility from abundant commercial sources. In addition, Pd-catalyzed C-H functionalization systems generally tolerate a variety of elements, and, therefore, stringent conditions such as oxygen- or moisture-free environments are not required, which enable the Pd-catalyzed system to be much more practical to deploy. These observations have been documented in several notable studies by leading researchers in the field, including Yu, Sanford, Dorta, and Poli [1–4]. Evidentially, there is a continuing need to explore new ligand entities that can enhance the reactivity and the selectivity of C-H activation reactions.

The direct functionalization of allylic alkenes has been the subject of extensive investigation, and, among many approaches, an efficient catalytic system involving the use of Pd metal and sulfoxide ligands was first explored by the Shi and White research groups, who employed a bissulfoxide ligand in palladium-catalyzed allylic amination and alkylation reactions [5–7]. Subsequently, the White group further investigated the usage of sulfoxideoxazoline ligands as an alternative for these transformations [8,9]. Meanwhile, Stambuli and Zhang demonstrated that a palladium sulfide catalyst can effectively catalyze allylic oxidation and oxidative Heck reactions [10,11]. These pioneering works represent significant advancements in the development of novel catalytic systems for direct functionalization of allylic alkenes.

**Figure 1.**
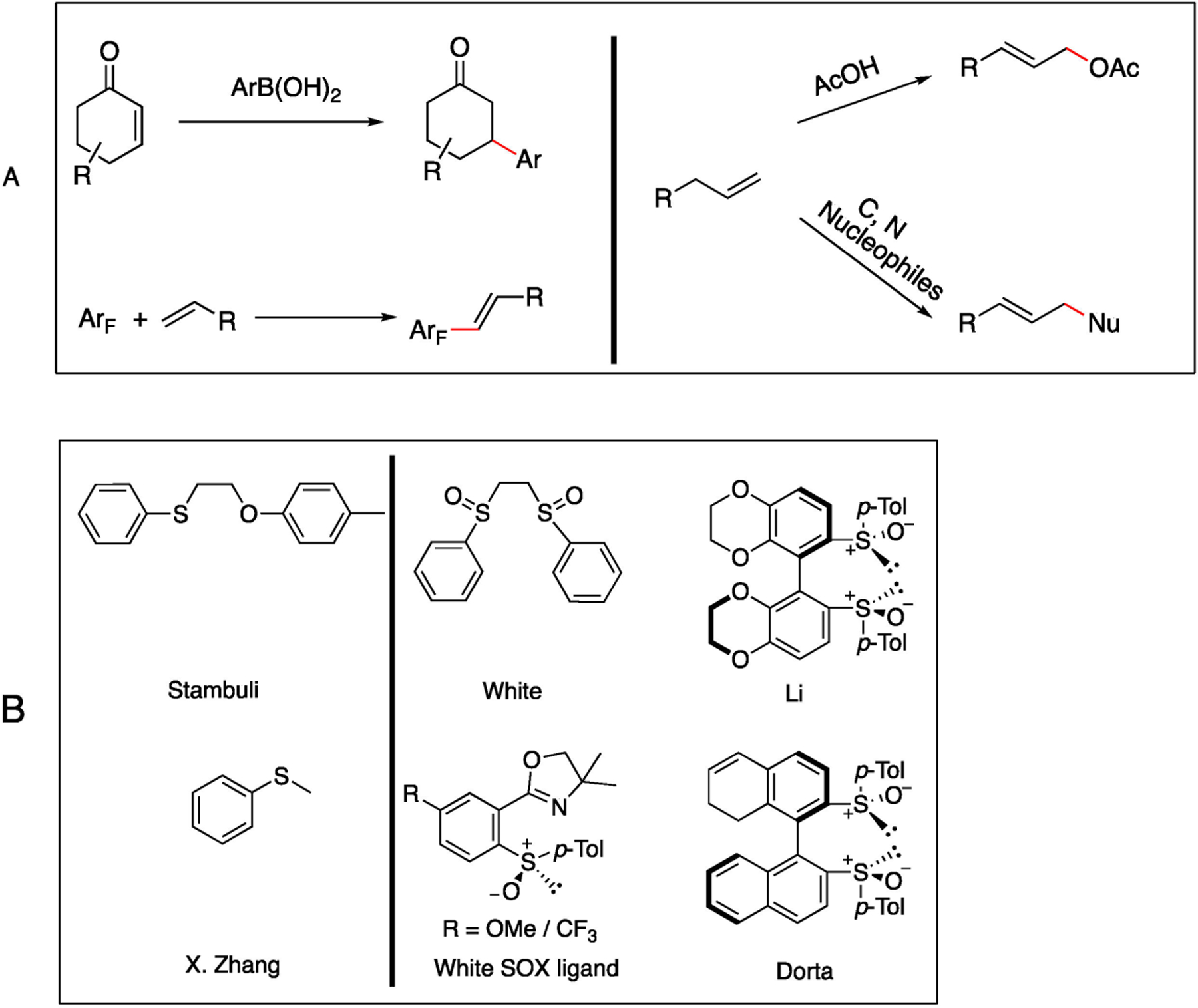
(**A**) Pd/Rh Thioether/Bissulfoxide Complexes Catalyzed Allylic Oxidation Reactions. (**B**) Notable Ligands Employed in C-H Activation [8–11,15,16].

Our group discovered a novel bicyclic [3.3.1] framework for primary amine labeling in 2019 [12]. Drawing inspiration from the Briphos ligand developed by Kim et al. [13], we recognized that the potential of our newly discovered framework may potentially serve as a novel class of geometrically constrained bisulfide/sulfoxide ligands. With the inclusion of a tertiary amine and two sulfide/sulfoxide moieties, we hypothesized that the framework might promote the formation of allylpalladium species while attracting amine nucleophiles to the reaction site [14]. Through extensive ligand screening for C-H activation, DMSO and bissulfoxide were proved as effective promoters for the formation of (σ-allyl)–palladium complexes with allyl compounds. These observations have been documented in several notable studies by researchers including Dorta [3] and White (Figure 1) [5]. In addition, the Dorta and Li groups utilized bissulfoxide ligand in Rh catalyzed addition of arylboronic acid to electron deficient olefins (Figure 1) [15,16]. Along this line, we herein report a direct benzyl-allylic functionalization through Pd-catalyzed C–H activation with a novel bissulfoxide ligand.

## 2. Material and Methods

Compounds **7**, **8** and **S3–9** are reported in [5,9,17–22]. All detailed experiment conditions and spectral data are available in Supplementary Materials.

X-ray crystal structure data collection was performed on a Bruker D8 VENTURE X-ray diffractometer with PHOTON 100 CMOS shutterless mode detector equipped with a Mo-target X-ray tube (*λ* = 0.71073 Å) at *T* = 100(2) K. Data reduction and integration were performed with the Bruker software package SAINT (version 8.38A). Data were corrected for absorption effects using the empirical methods as implemented in SADABS (version 2016/2). The structure was solved by SHELXT and refined by full-matrix leastsquares procedures using the Bruker SHELXTL (version 2017/1) software package. All non-hydrogen atoms were refined anisotropically [23–26].

## 3. Results and Discussion

**Scheme 1.**
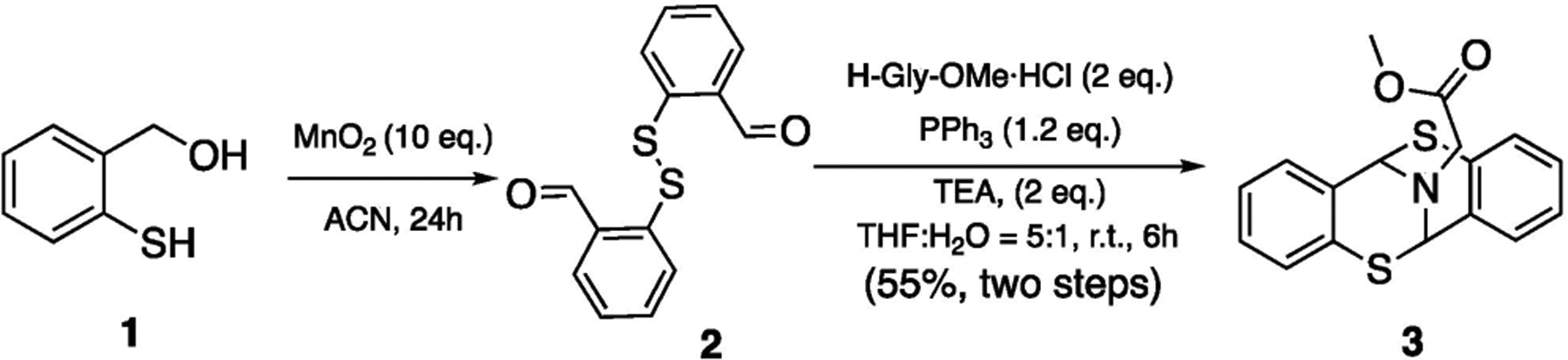
Rapid Construction of Bicyclic [3.3.1]-nonane Framework.

Derived from our protein conjugation study [12], it is revealed that the bicyclic [3.3.1] framework could be rapidly constructed (Scheme 1). Starting from commercially available thiophenol **1**, homo-disulfide **2** was furnished upon oxidation of compound **1**. In situ reduction of disulfide bond followed by a glycine amination meant bicyclic [3.3.1] nonane motif **3** could be obtained in high yield. Overall, substrate **3** was secured in two steps and features a strikingly geometrically constraint. In addition, the presence of a tertiary amine and sulfide moieties may potentially facilitate the formation of organometallic complexes.

Subsequently, we evaluated compound **3** as a potential ligand for C-H functionalization. Unfortunately, despite multiple attempts, the complex of palladium and **3** did not exhibit significant catalytic activity.

Motivated by a recent study [14], we hypothesized that introducing a sulfoxide group to the molecule could enhance its metal chelation ability. Thus, we explored the possibility of oxidizing compound **3** to its sulfoxide form (Table 1). The process of sulfoxide formation presented challenges, primarily due to difficulties of controlling the oxidation state of sulfides. An uncontrolled sulfide oxidation could potentially lead to moieties at different oxidation states. Sulfoxide, sulfone, and a combination of both could be produced as mixtures. Commonly employed oxidants such as Oxone, NaIO_4_, or PhI(OAc)_2_ yielded no product. Peroxide-based oxidants were evaluated and mCPBA furnished trace amounts of product in CH_2_Cl_2_, but, unfortunately, when other solvents, such as acetone and THF, could not produce any desired oxidation product. Alternatively, DMDO and Sharpless reagents failed to generate correct sulfoxide. We eventually resorted to H_2_O_2_, with the assistance from Na_2_WO_4_ as a co-oxidant, and the desired sulfoxide was finally furnished in comfortable yield. Furthermore, there is no correct product observed when acetone was replaced by other solvents (HOAc, MeOH, HFIP, and THF). Under the optimized conditions (H_2_O_2_, Na_2_WO_4_, Acetone), overoxidation was overcome and provided correct disulfide motif with a 51% isolation yield [27].

**Scheme 2.**
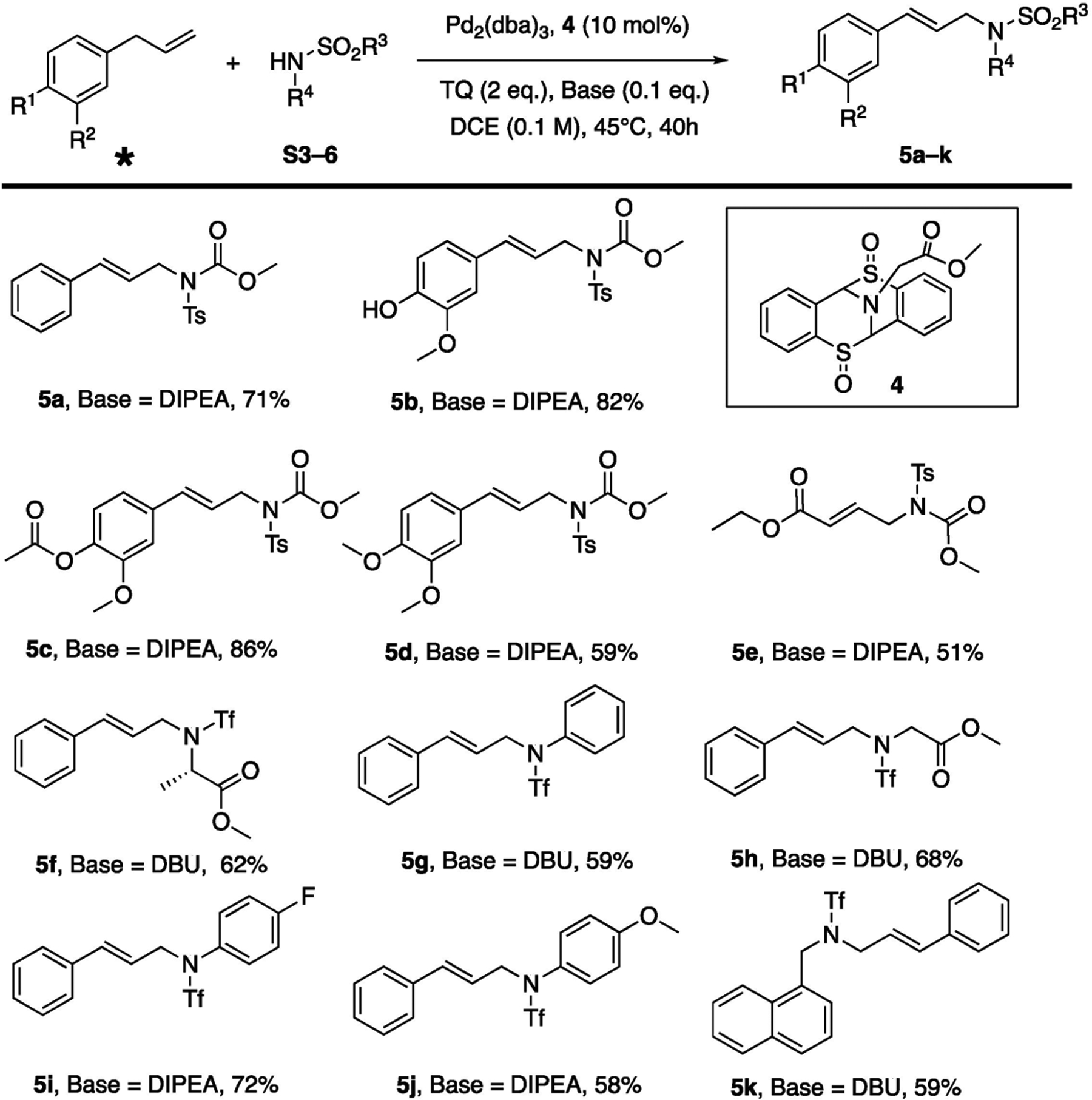
Pd-bissulfoxide Catalyzed Allylic C-H Amination. * Purchased from commercial sources.

After successfully obtaining the bissulfoxide ligand 4, the subsequent evaluation of bissulfoxide ligand with Palladium catalyst was immediately carried out. Luckily, the bissulfoxide moiety enabled the C-H activation of allyl benzene while utilizing amine as nucleophiles (Scheme 2). Two different bicyclic sulfoxide ligands were explored (**4** and see Supplementary Materials compound S2), and a list of palladium catalysts was examined (Pd(PPh_3_)_4_, and PdOAc_2_, Allylpalladium(II) chloride), and other additives were also investigated, including the addition of benzoquinones (BQs) and the usage of bases, and the reaction temperatures were then screened (see Supplementary Materials Section S4.2). After the initial evaluations, we concluded that a combination of ligand **4** and Pd_2_(dba)_3_ is a slightly superior complex compared to Pd_2_(dba)_3_/S2. The pair furnished the ideal outcomes in the presence of *p*-toluquinone (TQ) and base. Amination adduct **5a** was produced in good yield (71%) and reactions can be carried out within a relatively shorter reaction time (40 h) compared to other reported instances [5]. The electron-withdrawing induction effect of R^1^ groups seem to contribute to excellent yields of **5b** (82%) and **5c** (86%), while the methoxy group on R^1^ position, whose ability to donate electron density might overpower its induction ability, caused the lower yield of **5d** (59%). When comparing **5f** and **5h**, α-substituted aliphatic amine returned a diminished yield of 62% (**5f**). To achieve successful outcomes in C-H functionalization, electron withdrawing groups, such as tosyl and triflyl, are required to facilitate the transformation. Therefore, it is speculated that the pKa of the amine nucleophile plays a critical role in this process. Triflyl substituted amine nucleophiles provided corresponding C-H activation adducts in acceptable yields (58–72%, **5f**–**k**).

The absence of the palladium catalyst led to no reaction. In the effort to identify the role of ligand aromatic rings, analogous aliphatic bicyclic scaffold was prepared (S11), but, unfortunately, the oxidation of S11 to its bissulfoxide form was not successful and led to the decomposition of S11, which suggests the possible stabilizing effect of the aromatic ring in the oxidation process. Moreover, the contribution of base is critical, and no product could be obtained when base was not employed, and it could be fine-tuned to either DIPEA or DBU for optimal outcomes. Under the optimized conditions, removal of ligand **4** could generate products with much lower yields (S4.2). The replacement of the carboxylic ester group on the ligand with benzyl group did not impact ligand activity substantially (S4.2). Finally, ligands that possess alternative sulfur oxidation states (bis-sulfide, bis-sulfone, or mono-sulfoxide) failed to catalyze transformations in meaningful yields, and bissulfoxide is required in the ligand framework for an efficient C-H activation.

**Scheme 3.**
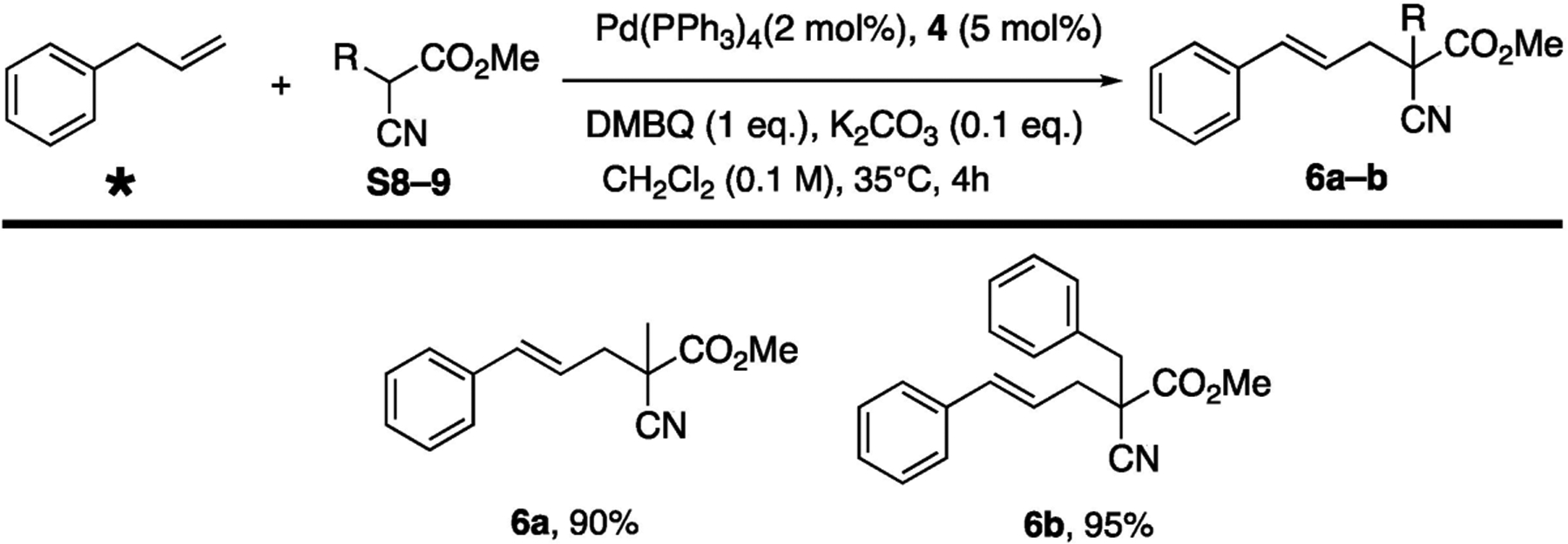
Pd-bissulfoxide Catalyzed Allylic C-H Alkylation. * Purchased from a commercial source.

The application of bissulfoxide ligand was investigated in the alkylative C-H activation settings (Scheme 3). Carbon nucleophile methyl cyanoacetate successfully rendered adduct in excellent yield (**6a**, 90%). Furthermore, adduct **6b** were prepared with benzyl cyanoacetate nucleophile at close to ambient temperature (35 °C/4 h). We were pleased to find out that catalytic loading could be reduced to 2 mol% of palladium and 5 mol% of ligand **4**, and that the absence of ligand **4** resulted in poor yields (S4.3). The trisubstituted nucleophiles are preferred, since disubstituted reagents (e.g., cyanoacetate) generated the mixture monoalkylated and di-alkylated products, which complicated the outcome elucidation.

**Figure 2.**
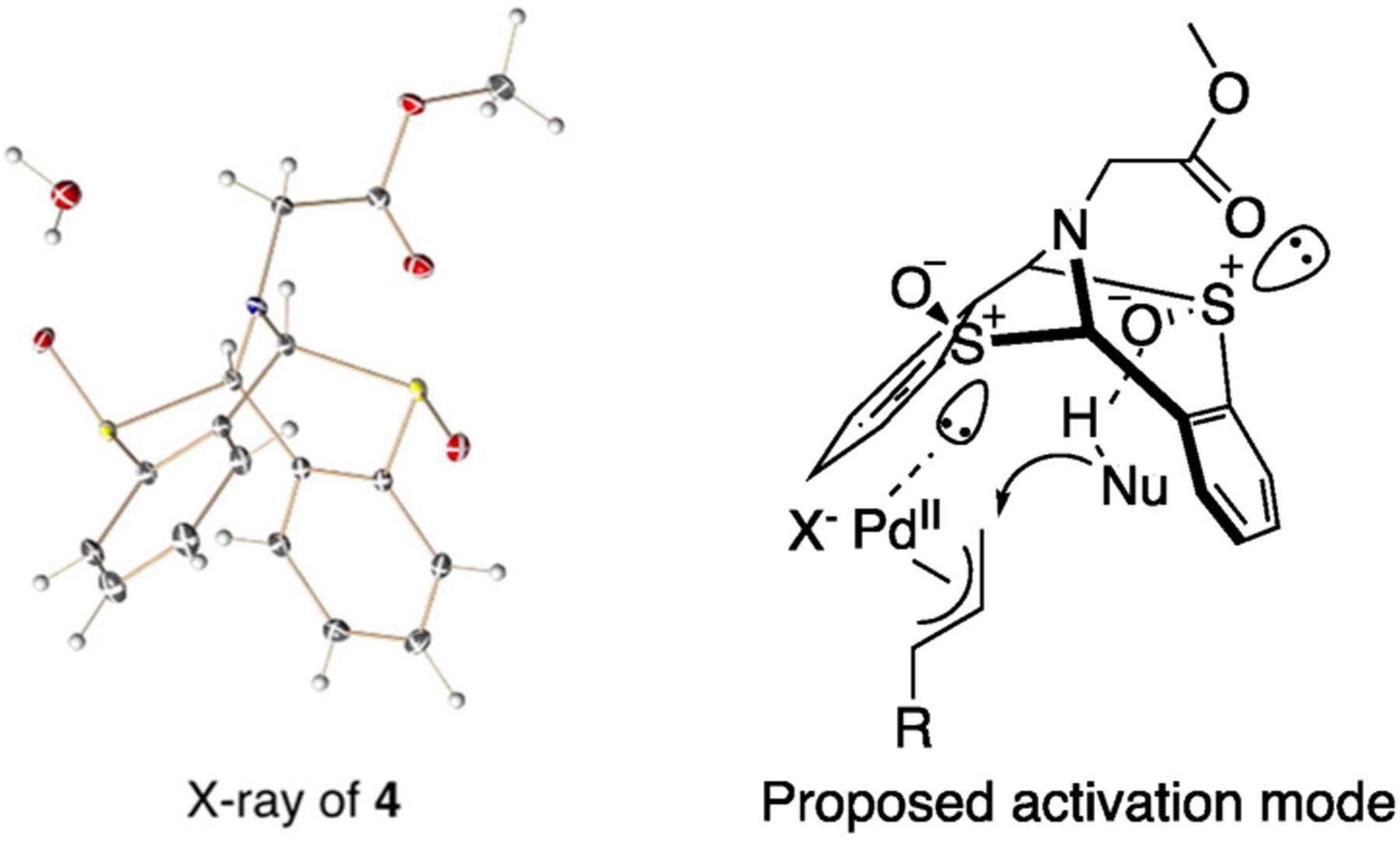
Proposed Hydrogen-bond Activated of Allylic Oxidation.

Mechanistically, attempts to identify the palladium and ligand complex were not successful after multiple attempts. Solution ^1^H NMR could not observe any distinct chemical shift change upon mixing Pd_2_(dba)_3_ with ligand at 35 °C. Regardless, despite the presence of TQ and allylbenzene in stoichiometric or catalytic amount, no coordination between Pd and ligand species were observed in NMR spectroscopy. Obtaining a single crystal of catalyst complex failed after multiple experiments. Fortunately, we were able to secure X-ray structures of ligand **4** and **S2**. Based on X-ray data and the literature precedence [14], the tentative proposed complex is demonstrated in Figure 2. Palladium sulfoxide (Pd-S) complex along with hydrogen bond between sulfoxide and nucleophiles facilitated the C-H functionalization.

**Scheme 4.**
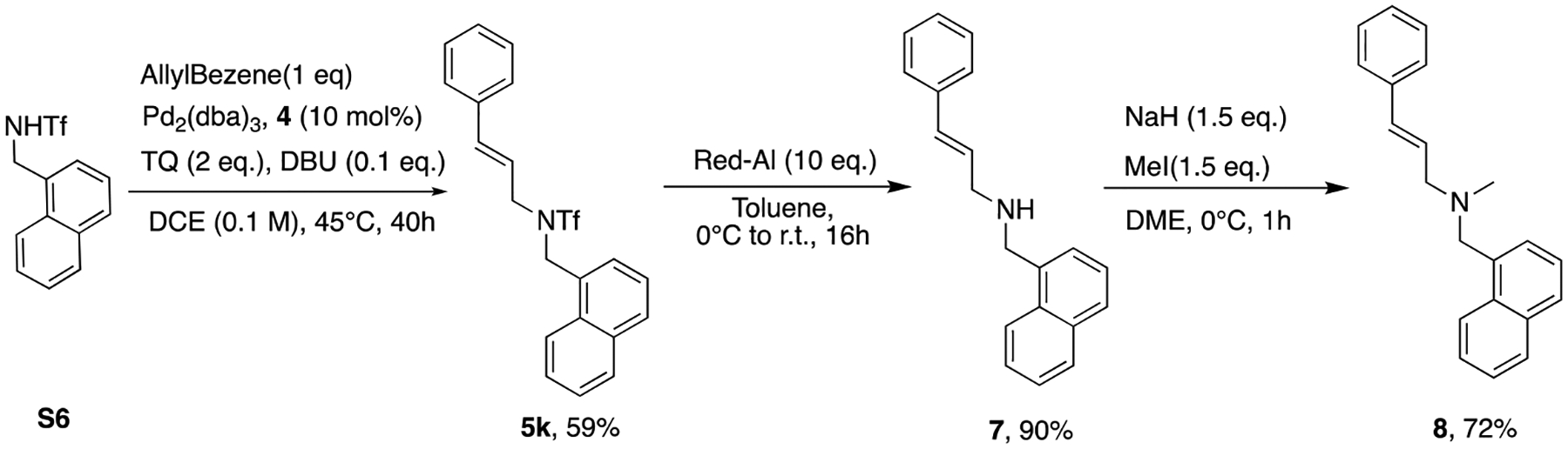
Preparation of Naftifine.

To illustrate the practicality of C-H functionalization tactic, a therapeutic agent, Naftifine [28] (treatment of fungal infection), was prepared in a concise manner (Scheme 4).Aforementioned adduct **5k** (Scheme 2) was successfully reduced to secondary amine **7** in 90% yield via Red-Al reduction. Subsequent *N*-methylation smoothly provided Naftifine **8** without any issue. Overall, the synthesis of Naftifine was completed in four steps from commercially available material with approximately 30% overall isolation yield boosted by this methodology.

## 4. Conclusions

Overall, in this study, we present a comprehensive investigation of a new class of sulfoxide ligands for C-H functionalization. The utilization of these ligands, featuring a unique [3.3.1] nonane scaffold, offer an intriguing avenue for catalyst activation. We have demonstrated that the combination of sulfoxides with this specific ligand architecture leads to remarkable catalytic outcomes. Despite the apparent structural complexity of the sulfoxide ligand, its chemical synthesis is surprisingly straightforward. By employing commercially available thiophenol, we accomplished the synthesis of the bissulfoxide ligand through a concise sequence of three synthetic steps, resulting in decent overall yields. Notably, compared to conventional sulfoxide ligands, the introduction of the bicyclic sulfoxide ligand leads to notable improvements in reaction kinetics, allowing for shorter reaction times and enhanced product yields. One of the notable advantages of our catalytic system is its compatibility with a wide range of nucleophiles, including both nitrogen-based (N) and carbon-based (C) nucleophiles. This broad substrate scope highlights the versatility and applicability of the developed ligand system. For successful C-H functionalization mediated by the bissulfoxide ligand, careful selection of the palladium source, specifically Pd_2_(dba)_3_, and the use of the appropriate additive, such as TQ, are crucial. Furthermore, the role of the base in the reaction system is found to be indispensable and plays a vital role in achieving the desired outcomes. To gain insights into the mechanism of our catalyst system, we propose a tentative activation mode that takes into account the unique features of the sulfoxide ligands and their interactions with the metal catalyst. This proposed mechanism provides a valuable framework for further exploration and refinement of our catalytic system. As a proof of concept, we applied our developed methodology to the rapid synthesis of the therapeutic agent Naftifine. By employing the C-H functionalization tactic in a concise four-step sequence, the success of obtaining this valuable compound highlights the synthetic utility and efficiency of our approach. Our ongoing efforts involve the introduction of chiral polypeptides and other heterocycles into the ligand motif, aiming to expand the range of accessible molecules and potentially enable enantioselective transformations.

## Supplementary Material

si

## Figures and Tables

**Table 1.** Sulfoxide Ligand Preparation.

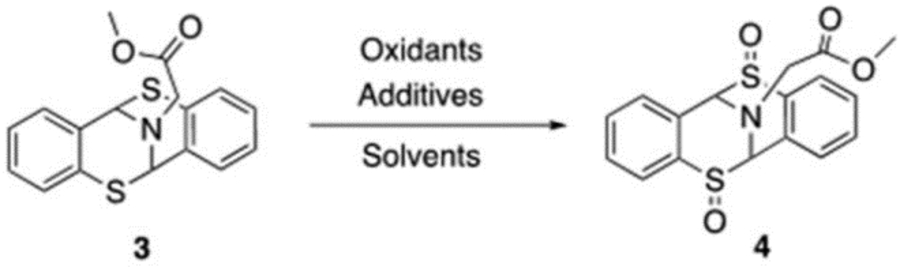					
Oxidants	Solvents	Additives	Temperature	Time	Yield
Oxone (2 eq.)	CH_2_Cl_2_	-	RT	O/N	N.D.
NaIO_4_ (2 eq.)	CH_2_Cl_2_	DDQ (2 eq.)	RT	O/N	N.D.
	MeOH	-			N.D.
PhI(OAc)_2_ (2 eq.)	MeOH	(NH4)_2_CO_3_(2eq.)	RT	O/N	N.D.
Sharpless reagents ^a^	-	-	0 °C	O/N	N.D.
DMDO (2 eq.)	-	-	0 °C	O/N	N.D.
m-CPBA (2 eq.)	CH_2_Cl_2_	-	0 °C	1h	<5%
		TBHP(0.1 eq.)	0 °C	1h	N.D.
	Acetone	-	0 °C	1h	N.D.
	THF	-	0 °C	1h	N.D.
H_2_O_2_ (2 eq.)	HOAc	-	RT	O/N	<10% conversion
	MeOH	-	RT	O/N	N.D.
	HFIP	-	RT	O/N	N.D.
	THF	Na_2_WO_4_·2H_2_O	RT	O/N	<5%
	Acetone:H_2_O ^b^	Na_2_WO_4_·2H_2_O	0 °C	50 min	51%

Room temperature (RT), Overnight (O/N), Not detected (N.D.), 2,3-Dichloro-5,6-dicyano-1,4-benzoquinone (DDQ), *t*BuOOH (TBHP), Dimethyldioxirane (DMDO). Conditions:

a Ti(OiPr)_4_ (1 eq.), (+)DET (4 eq.), TBHP (2 eq.).

b Acetone: H_2_O = 4:1, Na_2_WO_4_·2H_2_O (1 eq.), H_2_O_2_ (6 eq.).

## Data Availability

Complete experimental procedures and characterization data for all new compounds (PDF).
