# Supplementary material for: Palladium Catalyzed Allylic C-H Oxidation Enabled by Bicyclic Sulfoxide Ligands
Source: Organics. Author manuscript; Available in PMC 2024 Nov 27. (PMC11601123; doi:10.3390/org4020023)
Supplement: si [file NIHMS1994686-supplement-si.pdf]

Supporting Information

Palladium Catalyzed Allylic C-H Oxidation Enabled by Bicyclic Sulfoxide Ligands

Yuming Wen, Jianfeng Zheng, Alex H. Evans, and Qiang Zhang* Department of Chemistry, University at Albany, State University of
1400 Washington Avenue, Albany, New York, NY 12222, USA
E-mail: qzhang5@albany.edu

Table of Contents

1. **General Information: .... pg 2**
2. **Preparation of bicyclic ligands : .... pg 2**
3. **Preparation amine and carbon nucleophiles : .... pg 5**
   3.1. Preparation of N-Tf/Ts protected amine nucleophiles .... pg 5
   3.2. Preparation of tertiary carbon nucleophiles .... pg 7
4. **Reaction development of Pd-bissulfoxide catalyzed allylic C—H oxidation and control experiments : .... pg 8**
   4.1. Reaction development of Pd-bissulfoxide catalyzed allylic C—H amination
   4.2. Control experiments of Pd-bissulfoxide catalyzed allylic C—H amination
   4.3. Control experiments of Pd-bissulfoxide catalyzed allylic C—H alkylation
5. **Reaction scope: .... pg 9**
6. **Synthesis of Naftifine: .... pg 15**
7. **X-Ray Structure of 4 and S2: .... pg 16**
8. **NMR spectra: .... pg 21**

1. General Information

All commercially obtained reagents were used as received unless otherwise noted. All reagents were purchased from Sigma-Aldrich, Tokyo Chemical Industry, Acros, Alfa Aesar, Chemimpex, and Oakwood Chemicals which were used without further purification. The catalyst $Pd_2(dba)_3$ was purchased from Sigma-Aldrich and $Pd(PPh_3)_4$ from Ambeed. All allylic C–H amination and alkylation reactions were set up and run under ambient air with no precautions taken to exclude moisture. All other reactions were run in flame- or oven-dried glassware under an atmosphere of Ar gas with dry solvents unless otherwise stated. High-resolution mass spectra were obtained at the University at Albany- SUNY Core Facility Center using an Agilent G6530BA Q-TOF Mass Spectrometer. Analytical thin layer chromatography was performed on SiliCycle silica gel 60 F254 plates and flash column chromatography was performed on SiliaFlash P60, 40-63 µm, 60 Å (SiliCycle). For flash column chromatography purifications using triethylamine deactivated silica, a silica slurry was made with a solution of 3% triethylamine in hexanes (v/v). The slurry was added to the column, packed, and at least 2 column volumes of the starting eluent was passed through the column. The crude material was then introduced and purified, using the corresponding solvent(s) noted. Yields refer to chromatographically and spectroscopically pure materials unless otherwise stated. Abbreviations in the text are as follows: Allylbenzene (AB), 1,4-benzoquinone (BQ), dichloromethane (DCM), dichloroethane (DCE), 4,4′-Di-tert-butylbiphenyl (DTBBP), toluene (Tol), triethylamine (TEA) dimethylformamide (DMF), ethyl acetate (EtOAc), tetrahydrofuran (THF), hexanes (Hex), dimethyl sulfoxide (DMSO), 2,6-dimethylbenzoquinone (DMBQ), methyl tert-butyl ether (MTBE), methyl tosylcarbamate (MT), Methyl-p-benzoquinone (TQ), Trifluoromethanesulfonic anhydride (Tf_2O), trifluoromethanesulfonyl (Tf), trifluoroacetic acid/trifluroacetyl (TFA), toluenesulfonyl (Ts), room temperature (RT), and overnight (o/n).

NMR spectra were recorded on a Bruker Ascend 500 (500 MHz) for ^1H and ^{19}F, and (125 MHz) for proton decoupled ^{13}C using $CDCl_3$ (1H, 7.26 ppm; 13C, 77.12 ppm), CD_3OD (1H, 3.31 ppm; 13C, 49.00 ppm), and CD_3COCD_3(1H, ppm; 13C, ppm) as internal standard. Data reported as: s = singlet, d = doublet, t = triplet, q = quartet, quin. = quintet, sext. = sextet, sept. = septet, o = octet, m = multiplet, br = broad; coupling constant(s) in Hz.

2. Preparation of bicyclic ligands

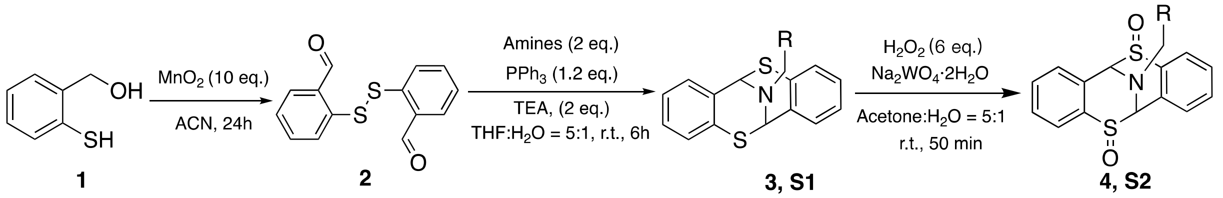

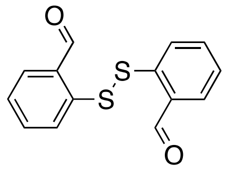

2,2'-disulfanediyldibenzaldehyde (2)

An 100 mL flask was charged with a stir bar, (2-mercaptophenyl)methanol (3 g, 21.4 mmol, 1 eq.) and anhydrous ACN (7 mL, 1 M). The reaction was cooled to 0 °C and MnO_2 (18.6 g, 214 mmol, 10 eq.) was added in portions and the reaction was allowed to warm up to RT and stirred at RT for 24 h. The reaction mixture was passed through celite and used in next step without purification.

^{1}H NMR (500 MHz, Chloroform-*d*) δ 10.22 (s, 2H), 7.87 (dd, *J* = 7.6, 1.5 Hz, 2H), 7.80 – 7.74 (m, 2H), 7.49 (ddd, *J* = 8.1, 7.3, 1.6 Hz, 2H), 7.39 (td, *J* = 7.4, 1.1 Hz, 2H). These data are in agreement with those previously reported in the literature [12].

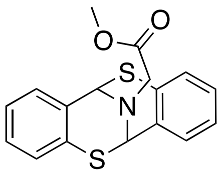

Methyl 2-(6*H*,12*H*-6,12-epiminodibenzo[*b*,*f*][1,5]dithiocin-13-yl)acetate (3)

To a solution of glycine methyl ester hydrochloride (1698 mg, 13.5 mmol, 2 eq.) in 5 mL water, was added TEA (1.89 mL, 13.5 mmol, 2 eq.), followed by the addition of **2** (1855 mg, 6.76 mmol, 1 eq.) and triphenylphosphine (2128 mg, 8.1 mmol, 1.2 eq.) in 24 mL of THF. The mixture was stirred at room temperature for 6 h, monitored by TLC. Upon completion, the resulting solution was concentrated under vacuum and extracted with ethyl acetate (10 mL×3). The combined the organic phase was washed with brine and dried over Na_2SO_4. Removing the solvent in vacuo, the residue was purified by silica gel column chromatography (Hex/DCM = 1/2 to 1/3) to give 1612 mg product in 55% yield.

^{1}H NMR (500 MHz, Chloroform-*d*) δ 8.24 – 6.40 (m, 8H), 5.53 (s, 2H), 3.87 (d, *J* = 17.2 Hz, 1H), 3.73 (s, 3H), 3.51 (d, *J* = 17.2 Hz, 1H). These data are in agreement with those previously reported in the literature [12].

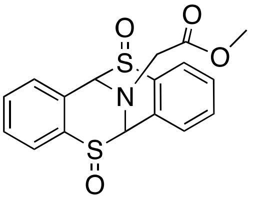

Methyl 2-(5,11-dioxido-6*H*,12*H*-6,12-epiminodibenzo[*b*,*f*][1,5]dithiocin-13-yl)acetate (4)

To a 250 mL oven-dried round-bottom flask was added the **3** (1.16 g, 3.5 mmol, 1 eq.), $Na_2WO_4 \cdot 2H_2O$ (1.16 g, 3.5 mmol, 1 eq.), Acetone (56 mL), and H_2O (12 mL). The solution was cooled to 0 °C, H_2O_2 (30~35% in H_2O, 21 mmol, 2.03 mL, 6 eq.) was then added. The reaction was slowly warmed to room temperature and stirred for 50 min. Upon completion, the reaction was quenched with sat. Na_2SO_3 solution at 0 °C. The resulting solution was extracted with ethyl acetate (30 mL×3). The combined the organic phase was washed with brine, dried over

Na_2SO_4, concentrated under vacuum, and subjected to a flash column chromatography (Hex/EA = 1/3 with 0.1% TEA) to provide 650 mg white powder in 51% yield.

^{1}H NMR (500 MHz, Acetone-*d*$_6$) δ 7.84 – 7.21 (m, 8H), 6.19 (s, 1H), 5.98 (s, 1H), 4.72 – 4.31 (m, 2H), 3.71 (s, 3H). **^{13}C NMR** (126 MHz, Acetone) δ 171.03, 141.68, 137.01, 133.31, 132.56, 130.70, 130.50, 130.02, 129.98, 128.54, 128.04, 126.89, 122.56, 76.95, 71.64, 58.95, 52.12. **HRMS** (ESI) *m/z* Calculated for $C_{17}H_{15}NO_4S_2Na$ $[M+Na]^+$: 384.0335, found 384.0368.

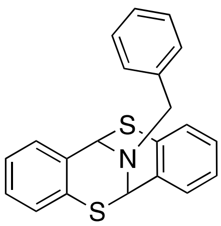

13-benzyl-6*H*,12*H*-6,12-epiminodibenzo[*b*,*f*][1,5]dithiocine (S1)

To a solution of glycine methyl ester hydrochloride (1698 mg, 13.5 mmol, 2 eq.) in 5 mL water, was added TEA (1.89 mL, 13.5 mmol, 2 eq.), followed by the addition of **2** (1855 mg, 6.76 mmol, 1 eq.) and triphenylphosphine (2128 mg, 8.1 mmol, 1.2 eq.) in 24 mL of THF. The mixture was stirred at room temperature for 6 h, monitored by TLC. Upon completion, the resulting solution was concentrated under vacuum and extracted with ethyl acetate (10 mL×3). The combined the organic phase was washed with brine and dried over Na_2SO_4. Removing the solvent in vacuo, the residue was purified by silica gel column chromatography (Hex/DCM = 1/2 to 1/3) to afford 816 mg product in 53.7% yield.

^{1}H NMR (500 MHz, Chloroform-*d*) δ 7.54 – 7.42 (m, 2H), 7.40 – 7.30 (m, 3H), 7.18 (dd, *J* = 7.4, 1.9 Hz, 2H), 7.11 – 6.97 (m, 6H), 5.38 (s, 2H), 4.17 (d, *J* = 13.5 Hz, 1H), 3.93 (d, *J* = 13.5 Hz, 1H). **^{13}C NMR** (126 MHz, $CDCl_3$) δ 136.60, 133.51, 130.18, 129.58, 129.55, 129.09, 128.26, 128.12, 127.99, 125.11, 61.84, 58.75. **HRMS** (ESI) *m/z* Calculated for $C_{21}H_{17}NS_2$ $[M+H]^+$: 348.0836, found 348.0877.

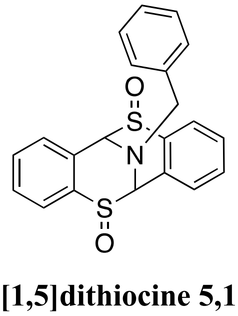

13-benzyl-6*H*,12*H*-6,12-epiminodibenzo[*b*,*f*][1,5]dithiocine 5,11-dioxide (S2)

To a 25 mL oven-dried round-bottom flask was added the **S1** (100 mg, 0.28 mmol, 1 eq.), $Na_2WO_4 \cdot 2H_2O$ (94.8 mg, 0.29 mmol, 1 eq.), Acetone (4.5 mL), and H_2O (0.9 mL). The solution was cooled to 0 °C, H_2O_2 (35% in H_2O, 2.24 mmol, 216 µL, 8 eq.) was then added. The reaction was slowly warmed to room temperature and stirred for 2h. Upon completion, the reaction was quenched with sat. Na_2SO_3 solution at 0 °C. The resulting solution was

extracted with ethyl acetate (30 mL×3). The combined the organic phase was washed with brine, dried over Na_2SO_4, concentrated under vacuum, and subjected to a flash column chromatography (Hex/EA = 1/3 with 0.1% TEA) to provide 90.9 mg product in 85% yield.

^{1}H NMR (400 MHz, Chloroform-*d*) δ 7.67 – 7.53 (m, 3H), 7.48 – 7.10 (m, 10H), 5.72 (s, 1H), 5.62 (s, 1H), 5.06 – 4.78 (m, 2H). **^{13}C NMR** (126 MHz, $CDCl_3$) δ 139.8, 136.5, 135.6, 133.6, 133.0, 130.7, 130.2, 130.1, 129.9, 129.3, 129.2, 128.8, 128.6, 126.6, 126.4, 120.8, 76.9, 70.0, 62.0. **HRMS** (ESI) *m/z* Calculated for $C_{21}H_{17}NO_2S_2$ $[M+H]^+$: 380.0734, found 380.0809. **IR** (neat): cm^{-1} 3060, 3029, 2940, 2872, 2855, 1716, 1452, 1437, 1359, 1271,1114, 1068, 1039, 1019, 753, 740, 697, 668.

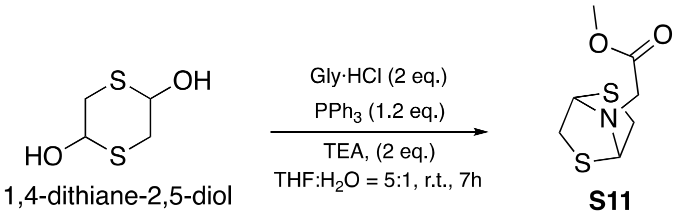

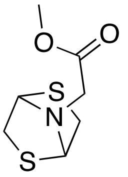

methyl 2-(2,5-dithia-7-azabicyclo[2.2.1]heptan-7-yl)acetate (S11)

To a solution of glycine methyl ester hydrochloride (330 mg, 2.6 mmol, 2 eq.) in 0.6 mL water, was added TEA (0.366 mL, 2.6 mmol, 2 eq.), followed by the addition of 1,4-dithiane-2,5-diol (200 mg, 1.3 mmol, 1 eq.) and triphenylphosphine (413 mg, 1.6 mmol, 1.2 eq.) in 3 mL of THF. The mixture was stirred at room temperature for 7 h, monitored by TLC. Upon completion, the resulting solution was concentrated under vacuum and extracted with ethyl acetate (3 mL×3). The combined the organic phase was washed with brine and dried over Na_2SO_4. Removing the solvent in vacuo, the residue was purified by silica gel column chromatography (Hex/EA = 1/5 to 1/4) to afford 160 mg product in 60% yield.

^{1}H NMR (500 MHz, Chloroform-*d*) δ 5.17 – 4.97 (m, 2H), 3.77 (s, 3H), 3.43 – 3.34 (m, 3H), 3.31 – 3.22 (m, 3H). **^{13}C NMR** (126 MHz, $CDCl_3$) δ 170.2, 70.5, 52.5, 50.1, 43.9. **HRMS** (ESI) *m/z* Calculated for $C_7H_{11}NO_2S_2$ $[M+H]^+$: 206.0304, found 206.1234.

3. Preparation of the amine and carbon nucleophiles

3.1. Preparation of N-Tf/Ts protected amine nucleophiles

General procedure for N-Tf protected amine nucleophiles:

Procedure A (From amines)

A flame dried round-bottom flask equipped with a stir bar was charged with amine (1.0 equiv), anhydrous DCM (0.5M) and TEA (1.1 equiv). The flask was cooled to -78 °C, and Tf_2O (1.0 equiv) was added dropwise. The reaction was stirred vigorously at -78 °C for 30 min, allowed to gradually warm up to RT and stirred o/n. The reaction was then quenched with H_2O. The reaction mixture was partitioned between H_2O and DCM and layers were separated. The aqueous layer was extracted with DCM three times. The organic layers were combined, dried over Na_2SO_4, filtered, and concentrated under reduced pressure. The concentrated organic layers was applied to a flash silica column for purification.

Procedure B (From amine hydrochlorides)

Same as procedure A except that 2.2 eq. of TEA was used.

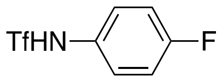

1,1,1-trifluoro-N-(4-fluorophenyl)methanesulfonamide (S3)

The reaction was performed according to general procedure A with 4-fluoroaniline.

^{1}H NMR (500 MHz, $CDCl_3$) δ 7.7 (br s, 1H), 7.47 (m, 2H), 7.26 (m, 2H). These data are in agreement with that previously reported in the literature [17].

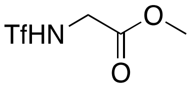

methyl ((trifluoromethyl)sulfonyl)glycinate (S4)

The reaction was performed according to general procedure B with glycine methyl ester hydrochloride.

^{1}H NMR (500 MHz, $CDCl_3$) δ 5.73 (br s, 1H), 4.07 (d, J = 4.57, 2H), 3.83 (s, 3H). These data are in agreement with that previously reported in the literature [18].

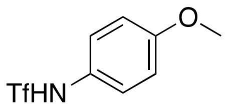

1,1,1-trifluoro-N-(4-methoxyphenyl)methanesulfonamide (S5)

The reaction was performed according to general procedure A with p-Anisidine.

^{1}H NMR (500 MHz, $CDCl_3$) δ 7.56-7.1 (br s, 1H), 7.23 (d, J = 8.99, 2H), 6.91 (d, J = 8.99, 2H), 3.82 (s, 3H). These data are in agreement with that previously reported in the literature [17].

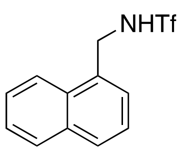

1,1,1-trifluoro-N-(naphthalen-1-ylmethyl)methanesulfonamide (S6)

The reaction was performed according to general procedure A with 1-Naphthylmethylamine.

^{1}H NMR (500 MHz, $CDCl_3$) δ 8.09-7.85 (m, 3H), 7.69-7.39 (m, 4H), 5.30 (br s, 1H), 4.88 (d, *J* = 5.4 Hz, 2H). These data are in agreement with that previously reported in the literature [9].

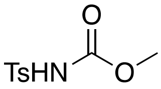

Methyl tosylcarbamate (S7)

To an oven dried round-botton flask equipped with a stir bar anhydrous MeOH was added. The flask was cooled to 0 °C and 4-methylbenzenesulfonyl isocyanate was added dropwise. The reaction was allowed to warm to RT and stirred for 3h. The reaction mixture was concentrated under reduced pressure and applied to a flash silica column for purification.

^{1}H NMR (500 MHz, $CDCl_3$) δ 8.73 (s, 1H), 7.94 (dd, *J* = 8.2, 2.5 Hz, 2H), 7.32 (dd, *J* = 8.4, 2.4 Hz, 2H), 3.67 (s, 2H), 2.41 (d, *J* = 2.7 Hz, 3H). These data are in agreement with that previously reported in the literature [5].

3.2. Preparation of tertiary carbon nucleophiles

General procedure for tertiary carbon nucleophiles:

A suspension of NaH (1.2 eq.) in THF is cooled at 0 °C in a flamed dried flask. Methyl cyanoacetate (1.0 eq.) was slowly added. The reaction was stirred for 30min at 0 °C. A solution of alkylhalide (1.2 eq.) in THF is added dropwise. The reaction was stirred at 0 °C for another 4h. The reaction was then quenched with sat. NH_4Cl. The reaction mixture was partitioned between H_2O and DCM and layers were separated. The aqueous layer was extracted with DCM three times. The organic layers were combined, dried over Na_2SO_4, filtered, and concentrated under reduced pressure. The concentrated reaction mixture was applied to a flash silica column for purification.

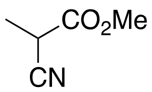

Methyl 2-cyanopropanoate (S8)

The reaction was performed according to general procedure with MeI.

^{1}H NMR (500 MHz, $CDCl_3$) δ 3.76 (s, 3H), 3.56 (q, *J* = 7.4 Hz, 1H), 1.53 (d, *J* = 7.5 Hz, 3H). These data are in agreement with that previously reported in the literature [19].

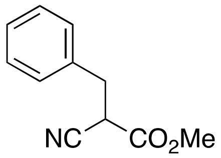

Methyl 2-cyano-3-phenylpropanoate (S9)

The reaction was performed according to general procedure with BnBr.

^{1}H NMR (500 MHz, $CDCl_3$) δ 7.41 – 7.27 (m, 5H), 3.82 (s, 3H), 3.77 (dd, J = 8.5, 5.7 Hz, 1H), 3.31 (dd, J = 13.9, 5.8 Hz, 1H), 3.22 (dd, J = 13.8, 8.5 Hz, 1H). These data are in agreement with that previously reported in the literature [20].

4. Reaction development of Pd-bissulfoxide catalyzed allylic C—H oxidation

4.1. Reaction development of Pd-bissulfoxide catalyzed allylic C—H amination

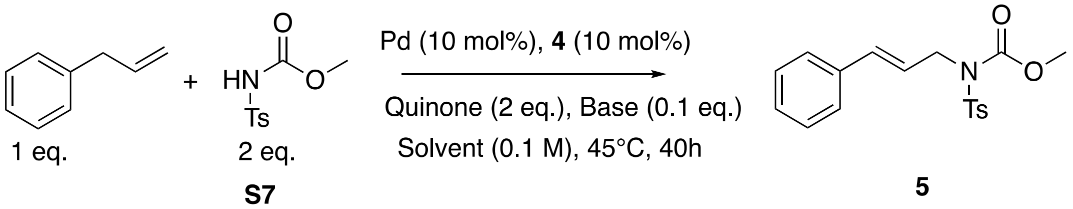

Entry	Pd	Quinone	Base	Solvent	Time (h)	%Yield
1[a]	$Pd(OAc)_2$	BQ	DIPEA	TBME	12	34
2	$Pd(OAc)_2$	BQ	DIPEA	DCE	45	33
3	$Pd_2(dba)_3$	BQ	DIPEA	DCE	16	43
4[b]	$Pd_2(dba)_3$	BQ	DIPEA	DCE	40	40
5	$Pd_2(dba)_3$	BQ	DIPEA	DCE	40	55
6	$Pd_2(dba)_3$	2,5-DMBQ	DIPEA	DCE	40	22
7	$Pd_2(dba)_3$	2,6-DMBQ	DIPEA	DCE	40	36
8	$Pd_2(dba)_3$	TQ	DIPEA	DCE	40	69

[a] 6% DIPEA, 0.66M TBME. [b] Nitrogen atomosphere.

4.2. Control experiments of Pd-bissulfoxide catalyzed allylic C—H Amination

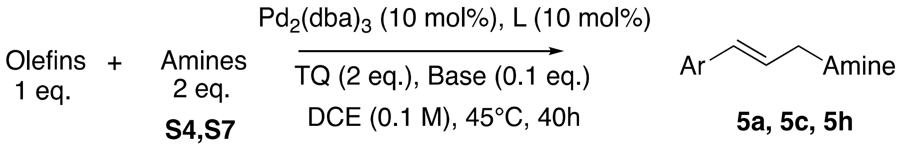

Entry	Olefin	Amine	Base	Ligand	%Yield
1	AB	S4	DBU	**4**	68
2	AB	S4	DBU	DMSO	28*
3	AB	S4	DBU	-	24*
4	Eugenol acetate	S7	DIPEA	**4**	86
5	Eugenol acetate	S7	DIPEA	DMSO	26*

6	Eugenol acetate	S7	DIPEA	-	22*
7	AB	S7	DIPEA	**S2**	52*

* NMR yields.

4.3. Control experiment of Pd-bissulfoxide catalyzed allylic C—H alkylation

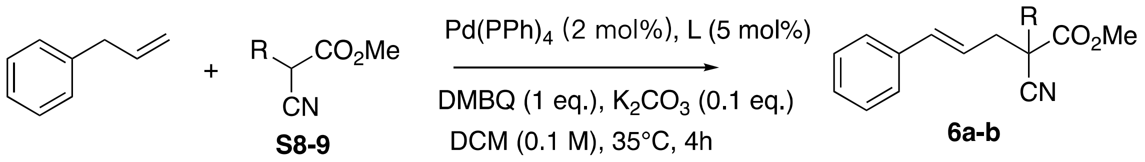

Entry	R	Ligand	%Yield
1	Bn	4	95
2	Bn	-	49
3	Me	4	90
4	Me	-	46

5. Reaction scope

General procedure:

Procedure A (amination)

To a 4 mL vial was added a stir bar, Pd_2dba_3 (9.2 mg, 0.01 mmol, 0.1 eq.), L (3.6 mg, 0.01 mmol, 0.1 eq.), TQ (22.4 mg, 0.2 mmol, 2 eq.), base (0.01 mmol, 0.1 eq.), olefin (0.1 mmol, 1.0 eq.) and N-Tf/Ts protected amine (0.2 mmol, 2 eq.). DCE (1 mL, 0.1 M) was added and the vial was capped and heated to 45 °C for 40 hours (monitored by TLC). The vial was allowed to cool to room temperature and diluted with a mixture of DCM and EtOAc. The reaction mixture was filtered through a pipette silica plug into a 20 mL vial with a mixture of DCM and EtOAc. The mixture was concentrated under reduced pressure, diluted with 2 mL $CDCl_3$ and analyzed by crude 1H NMR with an internal standard (DTBBP, 8 mg, 0.03 mmol, 0.3 eq.). The mixture was then concentrated under reduced pressure and subjected to flash column chromatography.

Procedure B (alkylation)

To a 4 mL vial was added a stir bar, $Pd(PPh_3)_4$ (3.5 mg, 0.003 mmol, 0.02 eq.), L (2.7 mg, 0.008 mmol, 0.05 eq.), DMBQ (20.4 mg, 0.1 mmol, 1 eq.), olefin (0.1 mmol, 1.0 equiv), K_2CO_3 (and carbon nucleophiles (0.1 mmol, 1.0 equiv). DCM (1 mL, 1.0 M) was added and the vial was capped and heated to 35 °C for 4 hours (monitored by TLC). The vial was allowed to cool to room temperature and diluted with a mixture of DCM and EtOAc. The reaction mixture was filtered through a pipette silica plug into a 20 mL vial with a mixture of DCM and EtOAc. The mixture was concentrated under reduced pressure, diluted with 2 mL $CDCl_3$ and analyzed by crude 1H NMR with an internal standard (DTBBP, 8 mg, 0.03 mmol, 0.3 eq.). The mixture was then concentrated under reduced pressure and subjected to flash column chromatography.

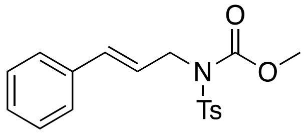

Methyl cinnamyl(tosyl)carbamate (5a)

The reaction was performed according to general procedure A with AB, MT and DIPEA as the base. Purification by flash column chromatography (Hex/EA = 4/1) to give 32.5 mg product in 92% yield as a light yellow thick oil.

^{1}H NMR (500 MHz, $CDCl_3$) δ 7.84 (d, *J* = 8.3 Hz, 2H), 7.63 – 6.98 (m, 7H), 6.67 (d, *J* = 15.8 Hz, 1H), 6.24 (dt, *J* = 15.9, 6.5 Hz, 1H), 4.62 (dd, *J* = 6.5, 1.3 Hz, 2H), 3.72 (s, 3H), 2.42 (s, 3H). These data are in agreement with that previously reported in the literature [5].

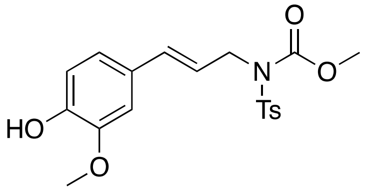

Methyl (*E*)-(3-(4-hydroxy-3-methoxyphenyl)allyl)(tosyl)carbamate (5b)

The reaction was performed according to general procedure A with eugenol, MT and DPIEA as the base. Purification by flash column chromatography (DCM/EA = 40/1) provided to give 32 mg product in 82% yield as a yellow solid.

^{1}H NMR (500 MHz, Chloroform-*d*) δ 7.84 (d, *J* = 8.3 Hz, 2H), 7.32 – 7.22 (m, 2H), 6.97 – 6.81 (m, 3H), 6.61 (dt, *J* = 15.8, 1.3 Hz, 1H), 6.10 (dt, *J* = 15.7, 6.6 Hz, 1H), 5.72 (s, 1H), 4.61 (dd, *J* = 6.6, 1.3 Hz, 2H), 3.91 (s, 3H), 3.73 (s, 3H), 2.42 (s, 3H). **^{13}C NMR** (126 MHz, $CDCl_3$) δ 152.8, 146.7, 145.9, 144.6, 136.6, 134.2, 129.4, 128.9, 128.6, 121.4, 120.5, 114.5, 108.6, 56.0, 53.9, 49.0, 21.6. **HRMS** (ESI) *m/z* Calculated for $C_{19}H_{21}NO_6SNa$ $[M+Na]^+$: 414.0982, found 414.0952.

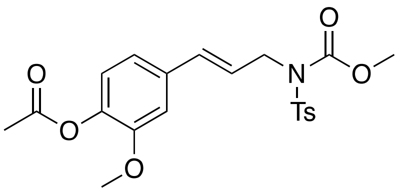

(*E*)-2-methoxy-4-(3-((*N*-(methoxycarbonyl)-4-methylphenyl)sulfonamido)prop-1-en-1-yl)phenyl acetate (5c)

The reaction was performed according to general procedure A with eugenol acetate, MT and DPIEA as the base. Purification by flash column chromatography (Hex/EA = 2/1 with 0.3% TEA) provided to give 37.2 mg product in 86% yield as a beige solid.

^{1}H NMR (500 MHz, Chloroform-*d*) δ 7.83 (d, *J* = 8.4 Hz, 2H), 7.34 – 7.23 (m, 2H), 7.04 – 6.90 (m, 3H), 6.71 – 6.58 (m, 1H), 6.19 (dt, *J* = 15.8, 6.5 Hz, 1H), 4.61 (dd, *J* = 6.5, 1.3 Hz, 2H), 3.84 (s, 3H), 3.71 (s, 3H), 2.41 (s, 3H), 2.31 (s, 3H). **^{13}C NMR** (126 MHz, $CDCl_3$) δ 169.0, 152.7, 151.2, 144.7, 139.6, 136.4, 135.3, 133.5, 129.4, 128.5, 124.1, 122.9, 119.2, 110.3, 55.9, 53.9, 48.7, 21.6, 20.7. **HRMS** (ESI) *m/z* Calculated for $C_{21}H_{23}NO_7SNa$ $[M+Na]^+$: 456.1088, found 456.1064.

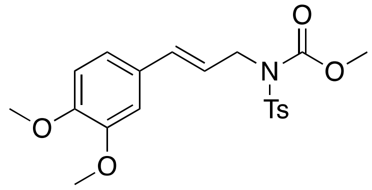

Methyl (*E*)-(3-(3,4-dimethoxyphenyl)allyl)(tosyl)carbamate (5d)

The reaction was performed according to general procedure A with 4-Allyl-1,2-dimethoxybenzene, MT and DPIEA as the base. Purification by flash column chromatography (1st: Hex/EA = 5/1, 2nd: Hex/EA = 2/1 with 0.3% TEA) to give 21.4 mg product in 58% yield as a white solid with a reddish tinge.

^{1}H NMR (500 MHz, $CDCl_3$) δ 7.86 (d, *J* = 8.4 Hz, 2H), 7.44 – 7.19 (m, 2H), 6.97 – 6.93 (m, 2H), 6.85 (d, *J* = 8.5 Hz, 1H), 6.64 (d, *J* = 15.8 Hz, 1H), 6.13 (dt, *J* = 15.7, 6.6 Hz, 1H), 4.62 (dd, *J* = 6.6, 1.3 Hz, 2H), 3.92 (s, 3H), 3.91 (s, 3H), 3.74 (s, 3H), 2.43 (s, 3H). **^{13}C NMR** (126 MHz, $CDCl_3$) δ 153.1, 149.5, 149.4, 144.9, 136.8, 134.3, 129.7, 128.9, 122.1, 120.2, 111.4, 109.3, 56.3, 56.2, 54.2, 49.3, 21.9. **HRMS** (ESI) *m/z* Calculated for $C_{20}H_{23}NO_6SNa$ $[M+Na]^+$: 428.1139, found 428.1153.

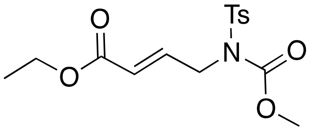

Ethyl (*E*)-4-((*N*-(methoxycarbonyl)-4-methylphenyl)sulfonamido)but-2-enoate (5e)

The reaction was performed according to general procedure A with ethyl but-3-enoate, MT and DPIEA as the base. Purification by flash column chromatography (Hex/EA = 9/1) followed by NaOH (1M) wash to give 17.3 mg product in 51% yield as a brown syrup.

^{1}H NMR (500 MHz, Chloroform-*d*) δ 7.83 (d, *J* = 8.4 Hz, 2H), 7.32 (dd, *J* = 8.6, 0.8 Hz, 2H), 6.91 (dt, *J* = 15.7, 5.3 Hz, 1H), 6.00 (d, *J* = 15.7 Hz, 1H), 4.60 (dd, *J* = 5.3, 1.7 Hz, 2H), 4.20 (q, *J* = 7.1 Hz, 2H), 3.71 (s, 3H), 2.44 (s, 3H), 1.30 (t, *J* = 7.2 Hz, 3H).**^{13}C NMR** (126 MHz, $CDCl_3$) δ 166.1, 152.7, 145.4, 142.2, 136.3, 129.8, 128.9, 123.8, 60.9, 54.4, 47.6, 22.0, 14.5. **HRMS** (ESI) *m/z* Calculated for $C_{15}H_{19}NO_6SNa$ $[M+Na]^+$: 364.0825, found 364.0848.

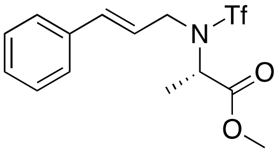

Methyl *N*-cinnamyl-*N*-((trifluoromethyl)sulfonyl)-*L*-alaninate (5f)

The reaction was performed according to general procedure A with AB, methyl ((trifluoromethyl)sulfonyl)-*L*-alaninate and DBU as the base. Purification by flash column chromatography (Hex/DCM = 1/1) to give 22 mg product in 62% yield as yellow oil.

^{1}H NMR (500 MHz, Chloroform-*d*) δ 7.65 – 7.11 (m, 5H), 6.58 (d, *J* = 15.9 Hz, 1H), 6.23 (dt, *J* = 14.9, 6.8 Hz, 1H), 4.68 (q, *J* = 7.3 Hz, 1H), 4.29 (s, 2H), 3.74 (s, 3H), 1.63 (d, *J* = 7.3 Hz, 3H). **^{13}C NMR** (126 MHz, $CDCl_3$) δ 170.6, 135.8, 134.7, 128.8, 128.4, 126.6, 123.6, 121.1, 118.5, 52.9. **^{19}F NMR** (471 MHz, $CDCl_3$) δ -75.80, -76.61.

Due to the peculiar appearances of certain peaks in carbon and fluorine NMR, we decided to conduct further investigation to verify the identity of **5f**. To our delight, all peaks resolved after reduction. Since **S10** is acquired from the reduction of **5f**, the structure of **5f** is indirectly proved.

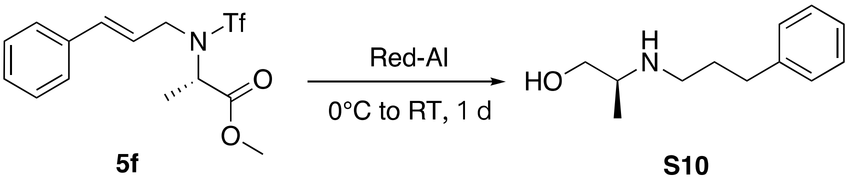

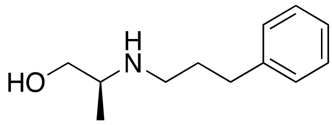

(*S*)-2-((3-phenylpropyl)amino)propan-1-ol (S10)

An flame-dried 10 ml flask was charged with a stir bar, **10** (48 mg, 0.14 mmol, 1 eq.) and toluene (3 ml, 0.05 M). The reaction was cooled to 0 °C and Red-Al (ca. 3.6 M in toluene) (sodium bis(2-methoxyethoxy)aluminumhydride) (1.4 mmol, 40 μL, 10 eq.) was added dropwise and the reaction was allowed to warm up to room temperature and stirred at room temperature for 24h. Upon completion, the reaction was cooled to 0 °C and 5% NH_4Cl was added. Layers were separated and the aqueous layer was further extracted with DCM (30 ml x 3). The organic layers were combined and dried over Na_2SO_4, filtered, concentrated under vacuum and subjected to a flash column chromatography (DCM w/ 10% of 7M ammonia in MeOH) to provide 18 mg pure product in 66% yield.

^{1}H NMR (500 MHz, Acetonitrile-*d*$_3$) δ 7.45 – 6.95 (m, 5H), 3.49 (dd, *J* = 10.9, 4.3 Hz, 1H), 3.42 – 3.35 (m, 3H), 3.31 (dd, *J* = 10.9, 6.7 Hz, 1H), 2.83 – 2.56 (m, 5H), 1.83 (m, 2H), 1.04 (d, *J* = 6.5 Hz, 3H). **^{13}C NMR** (126 MHz,

CD3CN) δ 142.6, 128.7, 128.7, 126.1, 64.9, 55.3, 46.2, 33.3, 31.4, 15.9. **HRMS** (ESI) *m/z* Calculated for $C_{12}H_{19}NO$ $[M+H]^+$: 194.1500, found 194.1558.

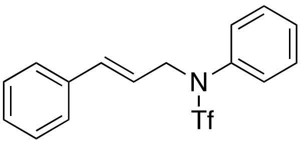

***N*-cinnamyl-1,1,1-trifluoro-*N*-phenylmethanesulfonamide (5g)**

The reaction was performed according to general procedure A with AB, 1,1,1-trifluoro-*N*-phenylmethanesulfonamide and DBU as the base. Purification by flash column chromatography (Hex/EA = 40/1) to give 21.5 mg product in 59% yield as a clear oil.

^{1}H NMR (500 MHz, Chloroform-*d*) δ 7.70 – 7.04 (m, 10H), 6.38 (d, *J* = 15.7 Hz, 1H), 6.14 (dt, *J* = 15.1, 6.9 Hz, 1H), 4.50 (d, *J* = 7.0 Hz, 2H). **^{13}C NMR** (126 MHz, $CDCl_3$) δ 136.9, 135.8, 129.5, 129.4, 129.3, 128.6, 128.3, 126.6, 122.1, 120.3 (q, *J* = 323.4 Hz), 56.0. **^{19}F NMR** (471 MHz, CDCl3) δ -74.08. **IR** (thin film): cm^{-1} 2958, 2922, 2852, 1491, 1454, 1389, 1226, 1186, 1141, 1058, 965, 892,765, 747, 691, 620.

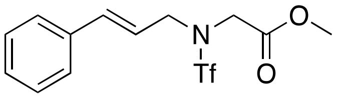

Methyl *N*-cinnamyl-*N*-((trifluoromethyl)sulfonyl)glycinate (5h)

The reaction was performed according to general procedure A with AB, methyl ((trifluoromethyl)sulfonyl)glycinate and DBU as the base. Purification by flash column chromatography (Hex/DCM = 1/1) to give 22 mg product in 68% yield as a white solid.

^{1}H NMR (500 MHz, Chloroform-*d*) δ 7.50 – 7.28 (m, 5H), 6.72 – 6.40 (m, 1H), 6.10 (dt, *J* = 15.8, 7.2 Hz, 1H), 4.33 – 4.25 (m, 2H), 4.12 (s, 2H), 3.76 (s, 3H). **^{13}C NMR** (126 MHz, $CDCl_3$) δ 168.1, 136.7, 135.5, 128.8, 128.7, 126.8, 121.4, 119.9 (q, *J* = 322.1 Hz), 52.7, 51.8, 47.2. **^{19}F NMR** (471 MHz, $CDCl_3$) δ -76.23. IR (thin film): cm^{-1} 1754, 1388, 1350, 1187, 1136, 1070, 971, 923, 784, 736, 691, 597.

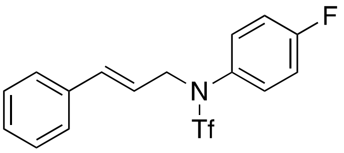

***N*-cinnamyl-1,1,1-trifluoro-*N*-(4-fluorophenyl)methanesulfonamide (5i)**

The reaction was performed according to general procedure A with AB, 1,1,1-trifluoro-*N*-(4-fluorophenyl)methanesulfonamide, and DIPEA as the base. Purification by flash column chromatography (Hex/EA = 40/1) to give 25.6 mg product in 72% yield as a brown oil.

^{1}H NMR (500 MHz, Chloroform-*d*) δ 7.38 – 7.13 (m, 7H), 7.04 (m, 2H), 6.34 (d, *J* = 15.8 Hz, 1H), 6.09 (dt, *J* = 15.7, 7.1 Hz, 1H), 4.43 (d, *J* = 7.1 Hz, 2H). **^{13}C NMR** (126 MHz, $CDCl_3$) δ 163.7, 161.7, 136.2, 135.6, 132.8, 132.8, 131.5, 131.4, 128.7, 128.7, 128.5, 126.7, 121.8, 120.3 (q, *J* = 323.8 Hz), 116.8, 116.6, 56.2. **^{19}F NMR** (471 MHz, $CDCl_3$) δ -74.09. ^{13}C NMR (126 MHz, $CDCl_3$) δ 120.38 (C, F decoupled). **IR** (thin film): cm^{-1} 2955, 2918, 2850, 1737, 1507, 1464, 1393, 1227, 1190, 1144, 1058, 967, 894, 819, 746, 693, 595.

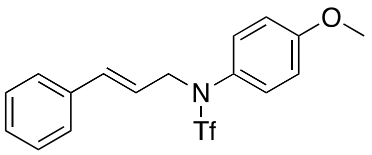

***N*-cinnamyl-1,1,1-trifluoro-*N*-(4-methoxyphenyl)methanesulfonamide (5j)**

The reaction was performed according to general procedure A with AB, 1,1,1-trifluoro-*N*-(4-methoxyphenyl)methanesulfonamide, and DIPEA as the base. Purification by flash column chromatography (Hex/DCM = 3/2) to give 21.4 mg product in 58% yield as clear oil.

^{1}H NMR (500 MHz, Chloroform-*d*) δ 7.48 – 7.13 (m, 7H), 6.89 (d, *J* = 8.9 Hz, 2H), 6.40 (d, *J* = 15.9 Hz, 1H), 6.16 (dt, *J* = 15.8, 7.1 Hz, 1H), 4.47 (d, *J* = 7.1 Hz, 2H), 3.80 (s, 3H). **^{13}C NMR** (126 MHz, $CDCl_3$) δ 160.0, 135.9, 135.8, 130.7, 129.3, 128.7, 128.4, 126.7, 122.3, 120.4 (q, *J* = 324.1 Hz), 114.7, 56.2, 55.5. **^{19}F NMR** (471 MHz, $CDCl_3$) δ -74.09. **IR** (thin film): cm^{-1} 2918, 2850, 1508, 1388, 1252, 1182, 1143, 1058, 1032, 967, 894, 831, 799, 748, 694, 676, 594.

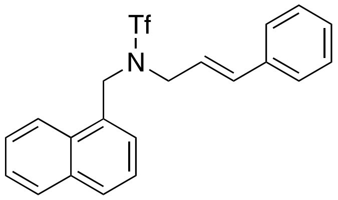

***N*-cinnamyl-1,1,1-trifluoro-*N*-(naphthalen-1-ylmethyl)methanesulfonamide (5k)**

The reaction was performed according to general procedure A with AB, 1,1,1-trifluoro-*N*-(naphthalen-1-ylmethyl)methanesulfonamide, and DBU as the base. Purification by flash column chromatography (Hex/EA = 30/1) to give 23.7 mg product in 59% yield as clear oil.

^{1}H NMR (500 MHz, Chloroform-*d*) δ 8.17 (d, *J* = 8.5 Hz, 1H), 7.96 – 7.84 (m, 2H), 7.65 – 7.48 (m, 4H), 7.36 – 7.25 (m, 3H), 7.22 – 7.13 (m, 2H), 6.21 (d, *J* = 15.8 Hz, 1H), 5.98 (dt, *J* = 15.8, 7.0 Hz, 1H), 5.11 (br s, 2H), 4.07 (br s, 2H). **^{13}C NMR** (126 MHz, $CDCl_3$) δ 136.1, 135.6, 133.9, 131.5, 129.6, 129.1, 129.0, 128.6, 128.4, 127.4, 127.0, 126.6, 126.3, 125.2, 122.9, 121.8, 120.3 (q, *J* = 323.6 Hz), 50.0, 49.2. **^{19}F NMR** (471 MHz, $CDCl_3$) δ -75.07. **HRMS** (ESI) *m/z* Calculated for $C_{21}H_{18}F_3NO_2SNa$ $[M+Na]^+$: 428.0903, found 428.0906.

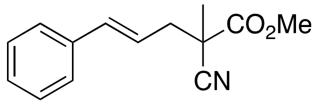

Methyl (*E*)-2-cyano-2-methyl-5-phenylpent-4-enoate (6a)

The reaction was performed according to general procedure B with AB, **S8**, and K_2CO_3 as the base. Purification by flash column chromatography (Hex/EA = 40/1) to give 20.8 mg product in 90% yield as clear oil.

^{1}H NMR (500 MHz, Chloroform-*d*) δ 7.52 – 7.22 (m, 4H), 6.58 (d, *J* = 15.7 Hz, 1H), 6.20 (dt, *J* = 15.7, 7.5 Hz, 1H), 3.84 (s, 3H), 2.86 (ddd, *J* = 13.8, 7.5, 1.3 Hz, 1H), 2.70 (ddd, *J* = 13.8, 7.6, 1.3 Hz, 1H), 1.66 (s, 3H). **^{13}C NMR** (126 MHz, $CDCl_3$) δ 169.5, 136.4, 135.9, 128.7, 128.0, 126.5, 121.6, 119.7, 53.6, 44.0, 41.5, 22.9. **HRMS** (ESI) *m/z* Calculated for $C_{14}H_{15}NO_2$ $[M+H]^+$: 230.1176, found 230.1213.

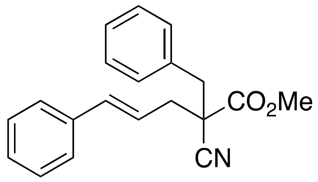

Methyl (*E*)-2-benzyl-2-cyano-5-phenylpent-4-enoate (6b)

The reaction was performed according to general procedure B with AB, **S9**, and K_2CO_3 as the base. Purification by flash column chromatography (Hex/EA = 40/1, Hex/DCM = 1:1, and then Hex/Acetone = 15:1) to give 29.6 mg product in 95% yield as clear oil.

^{1}H NMR (400 MHz, Chloroform-*d*) δ 7.55 – 7.02 (m, 8H), 6.59 (d, *J* = 15.7 Hz, 1H), 6.22 (dt, *J* = 15.4, 7.5 Hz, 1H), 3.68 (s, 3H), 3.28 (d, *J* = 13.4 Hz, 1H), 3.14 (d, *J* = 13.5 Hz, 1H), 2.92 (ddd, *J* = 13.8, 7.8, 1.3 Hz, 1H), 2.76 (ddd, *J* = 13.8, 7.2, 1.3 Hz, 1H). **^{13}C NMR** (126 MHz, $CDCl_3$) δ 168.5, 136.2, 135.7, 133.9, 129.7, 128.5, 128.4, 127.8, 126.4, 121.3, 118.4, 53.1, 51.5, 42.5, 40.6. **HRMS** (ESI) *m/z* Calculated for $C_{20}H_{19}NO_2Na$ $[M+Na]^+$: 328.1309, found 328.1344.

6. Synthesis of Naftifine.

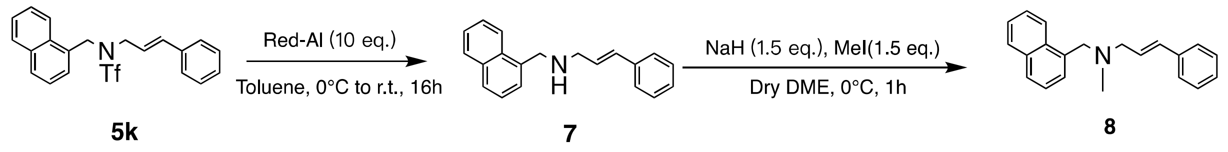

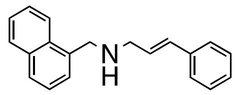

(*E*)-*N*-(naphthalen-1-ylmethyl)-3-phenylprop-2-en-1-amine (7)

An flame-dried 25 ml flask was charged with a stir bar, **15** (139 mg, 0.34 mmol, 1 eq.) and toluene (7 ml, 0.05 M). The reaction was cooled to 0 °C and Red-Al (ca. 3.6 M in toluene) (sodium bis(2-

methoxyethoxy)aluminumhydride) (3.43 mmol, 950 µL, 10 eq.) was added dropwise and the reaction was allowed to warm up to room temperature and stirred at room temperature o/n. Upon completion, the reaction was cooled to 0 °C and 5% NH_4Cl was added. Layers were separated and the aqueous layer was further extracted with DCM (30 ml x 3). The organic layers were combined and dried over Na_2SO_4, filtered, concentrated under vacuum and subjected to a flash column chromatography (Hex/EA = 3/1 w/ 2% TEA) to provide 85 mg pure product in 90% yield.

^{1}H NMR (500 MHz, Chloroform-*d*) δ 8.21 (dd, *J* = 8.4, 1.1 Hz, 1H), 8.00 – 7.89 (m, 1H), 7.85 (d, *J* = 8.1 Hz, 1H), 7.68 – 7.23 (m, 9H), 6.65 (d, *J* = 15.9 Hz, 1H), 6.44 (dt, *J* = 15.8, 6.3 Hz, 1H), 4.35 (s, 2H), 3.60 (dd, *J* = 6.4, 1.5 Hz, 2H). Spectral data for **7** was consistent with previously literature report [21].

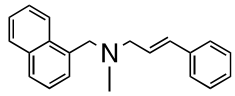

(*E*)-*N*-methyl-*N*-(naphthalen-1-ylmethyl)-3-phenylprop-2-en-1-amine (8)

An flame-dried 10 ml flask was charged with a stir bar, **18** (35 mg, 0.128 mmol, 1 eq.), 1,2-dimethoxyethane (640 µL, 0.2 M) and TEA (178 µL, 1.28 mmol, 10 eq.). NaH (60% Dispersion in Mineral Oil, 7.68 mg, 0.192 mmol, 1.5 eq.) was added under Ar at 0 °C, and the reaction mixture was stirred for 1h at 0 °C. Methyl iodide (27.3 mg, 0.192 mmol, 1.5 eq.) was added, and the mixture was allowed to warm to RT, and stirred for another 4h with occasional monitoring by TLC. After addition of water, the whole was extracted with DCM. The organic layers were combined and dried over Na_2SO_4, filtered, concentrated under vacuum and subjected to a flash column chromatography (Hex/EA = 20/1) to provide 26.6 mg pure product in 72.3% yield.

^{1}H NMR (500 MHz, Chloroform-*d*) δ 8.30 (d, *J* = 8.4 Hz, 1H), 7.84 (d, *J* = 1.5 Hz, 1H), 7.78 (d, *J* = 8.1 Hz, 1H), 7.60 – 7.12 (m, 9H), 6.58 (d, *J* = 15.9 Hz, 1H), 6.38 (dt, *J* = 15.9, 6.7 Hz, 1H), 3.96 (s, 2H), 3.29 (dd, *J* = 6.6, 1.4 Hz, 2H), 2.28 (s, 3H). Spectral data for **8** was consistent with previously literature report [22].

7. X-ray Crystal structure analysis of 4 and S2

Data collection was performed on a Bruker D8 VENTURE X-ray diffractometer with PHOTON 100 CMOS shutterless mode detector equipped with a Mo-target X-ray tube (λ = 0.71073 Å) at T = 100(2) K. Data reduction and integration were performed with the Bruker software package SAINT (version 8.38A).[10] Data were corrected for absorption effects using the empirical methods as implemented in SADABS (version 2016/2).[11] The structure was solved by SHELXT[12] and refined by full-matrix least-squares procedures using the Bruker SHELXTL (version 2017/1)[13] software package. All non-hydrogen atoms were refined anisotropically.

7.1. X-ray Crystal structure analysis of 4

Two H-atoms of water molecule were found in the difference Fourier map. Their positions were refined independently while their atomic displacement parameters were constrained with U_{iso}(H) = 1.5 U_{eq}(O). Other H-

atoms were included at calculated positions and refined as riders, with U_{iso}(H) = 1.2 U_{eq}(C) and U_{iso}(H) = 1.5 U_{eq}(C) for methyl groups. Further crystal and data collection details are listed in Table 1. The structure of the compound is shown in Figure 1. The compound crystallizes in the $P2_1/n$ (No. 14). In the structure, there are three chiral carbons (C4 and C11) and their chiralities are R and R, respectively.

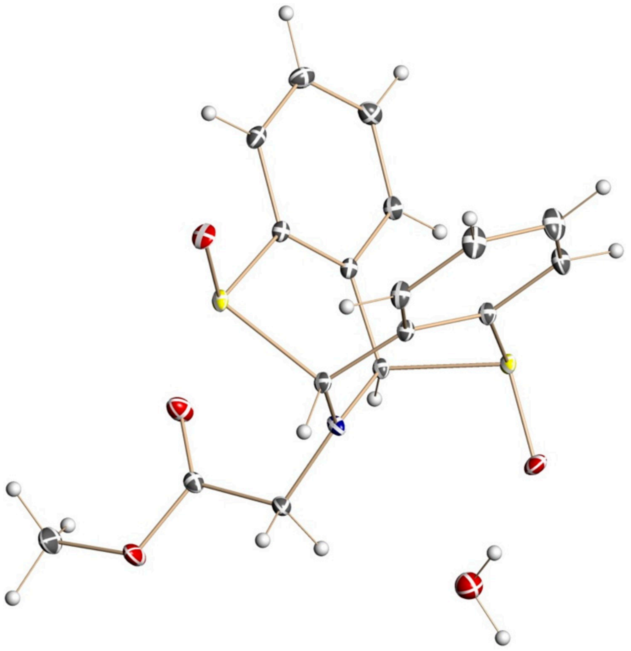

Figure S1. ORTEP drawing of **str2515** drawn with thermal ellipsoids at the 40% probability level. Color scheme used: C: grey; O: red; H: white; N: blue; S: yellow.

Table S1. Experimental details

Crystal data	
Chemical formula	$C_{17}H_{15}NO_4S_2 \cdot H_2O$
M_r	379.43
Crystal system, space group	Monoclinic, $P2_1/n$
Temperature (K)	100
a, *b*, *c* (Å)	12.4260 (5), 7.4787 (3), 17.7176 (8)
β (°)	90.672 (2)
V (Å^3)	1646.39 (12)
Z	4
Radiation type	Mo *K*α
μ (mm^{-1})	0.35

Crystal size (mm)	0.52 × 0.13 × 0.05
Data collection	
Diffractometer	Bruker D8 Venture PHOTON 100 CMOS diffractometer
Absorption correction	Multi-scan<br>*SADABS2016*/2 (Bruker,2016/2) was used for absorption correction. Krause, L., Herbst-Irmer, R., Sheldrick G.M. & Stalke D., J. Appl. Cryst. 48 (2015) 3-10
T_{min}, T_{max}	0.932, 1
No. of measured, independent and observed [$I > 2\sigma(I)$] reflections	75549, 7874, 6121
R_{int}	0.060
$(\sin \theta/\lambda)_{max}$ (Å^{-1})	0.834
Refinement	
$R[F^2 > 2\sigma(F^2)]$, $wR(F^2)$, S	0.054, 0.120, 1.06
No. of reflections	7874
No. of parameters	233
No. of restraints	2
H-atom treatment	H atoms treated by a mixture of independent and constrained refinement
$\Delta\rho_{max}$, $\Delta\rho_{min}$ (e Å^{-3})	0.68, −0.56

Computer programs: *APEX3* v.2017.3-0 (Bruker AXS Inc., 2017), *SAINT* V.8.38A (Bruker AXS Inc., 2017), SHELXT 2014/5 (Sheldrick, 2014), *SHELXL2017*/1 (Sheldrick, 2017), Xshell v.6.3.1 (Bruker AXS Inc., 2016).[10-13]

7.2. X-ray Crystal structure analysis of S2

H-atoms were included at calculated positions and refined as riders, with U_{iso}(H) = 1.2 U_{eq}(C) and U_{iso}(H) = 1.5 U_{eq}(C) for methyl groups. One of the molecules were found to be disordered and was modelled with two

orientations with relative occupancies of 0.61:0.39 for the two parts.Further crystal and data collection details are listed in Table 1. The structure of the compound is shown in Figure 1. The compound crystallizes in the *P*-1 (No. 2). In the each molecule, there are two chiral carbons and their chiralities are R and R, respectively.

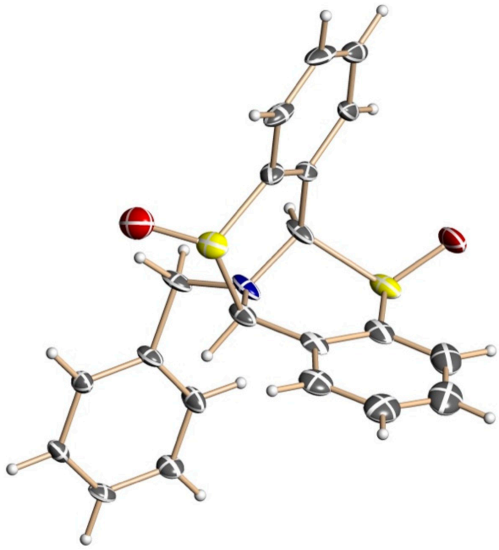

Figure S2. ORTEP drawing of **str2517** drawn with thermal ellipsoids at the 40% probability level. Color scheme used: C: grey; O: red; H: white; N: blue; S: yellow.

Table S2 Experimental details

Crystal data	
Chemical formula	$C_{21}H_{17}NO_2S_2$
M_r	379.48
Crystal system, space group	Triclinic, *P*-1
Temperature (K)	100
a, *b*, *c* (Å)	11.1753 (16), 12.3448 (18), 14.379 (2)
α, β, γ (°)	69.734 (2), 70.618 (2), 88.786 (3)
V (Å^3)	1745.6 (4)
Z	4
Radiation type	Mo *K*α
μ (mm^{-1})	0.32
Crystal size (mm)	0.24 × 0.16 × 0.02

Data collection	
Diffractometer	Bruker D8 Venture PHOTON 100 CMOS diffractometer
Absorption correction	Multi-scan<br>*SADABS2016*/2 (Bruker,2016/2) was used for absorption correction. Krause, L., Herbst-Irmer, R., Sheldrick G.M. & Stalke D., J. Appl. Cryst. 48 (2015) 3-10
T_{min}, T_{max}	0.916, 1
No. of measured, independent and observed [$I > 2\sigma(I)$] reflections	27978, 7153, 4686
R_{int}	0.074
$(\sin \theta/\lambda)_{max}$ (Å^{-1})	0.627
Refinement	
$R[F^2 > 2\sigma(F^2)]$, $wR(F^2)$, S	0.109, 0.308, 1.03
No. of reflections	7153
No. of parameters	680
No. of restraints	1784
H-atom treatment	H-atom parameters constrained
	$w = 1/[\sigma^2(F_o^2) + (0.1498P)^2 + 11.3856P]$<br>where $P = (F_o^2 + 2F_c^2)/3$
$\Delta\rho_{max}$, $\Delta\rho_{min}$ (e Å^{-3})	2.68, −1.14

Computer programs: *APEX3* v.2017.3-0 (Bruker AXS Inc., 2017), *SAINT* V.8.38A (Bruker AXS Inc., 2017), SHELXT 2014/5 (Sheldrick, 2014), *SHELXL2017*/1 (Sheldrick, 2017), Xshell v.6.3.1 (Bruker AXS Inc., 2016).[10-13]

8. NMR spectra

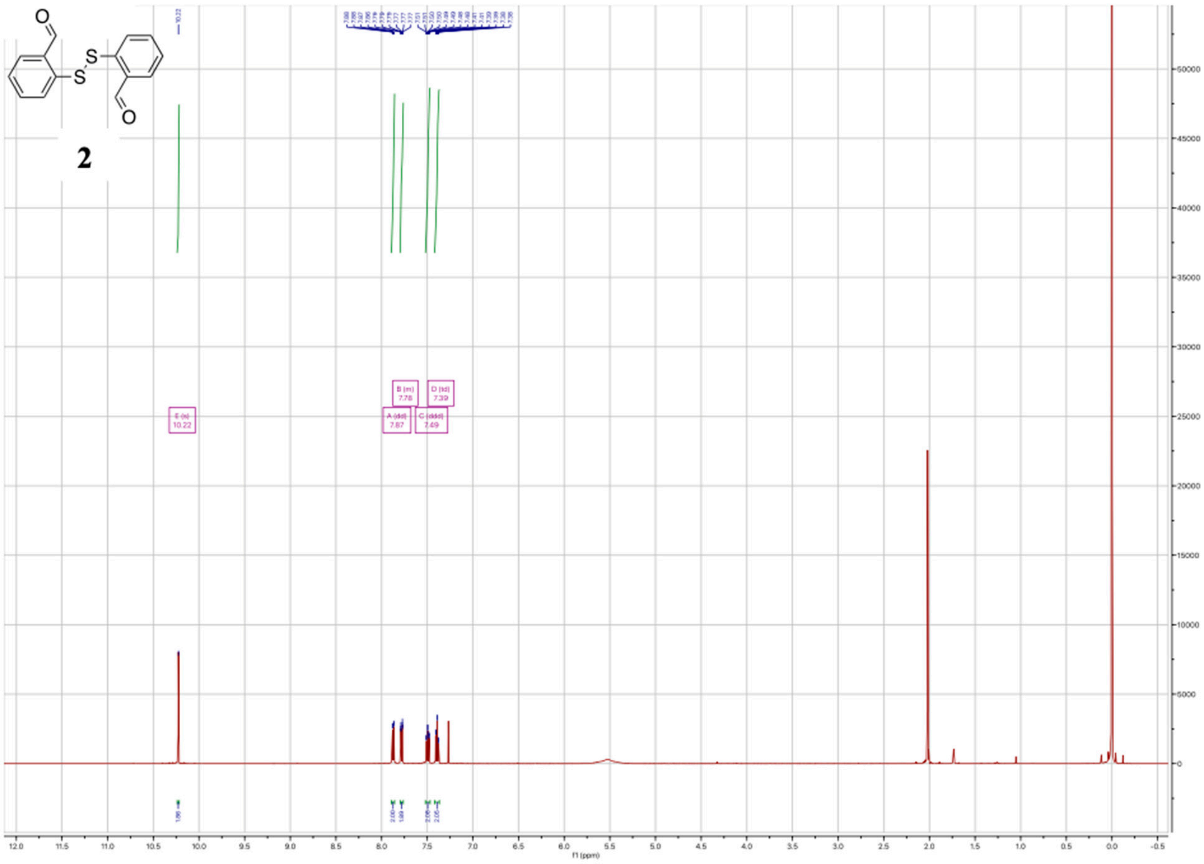

Figure S3: **^{1}H NMR** (500 MHz, Chloroform-*d*) spectrum of **2.**

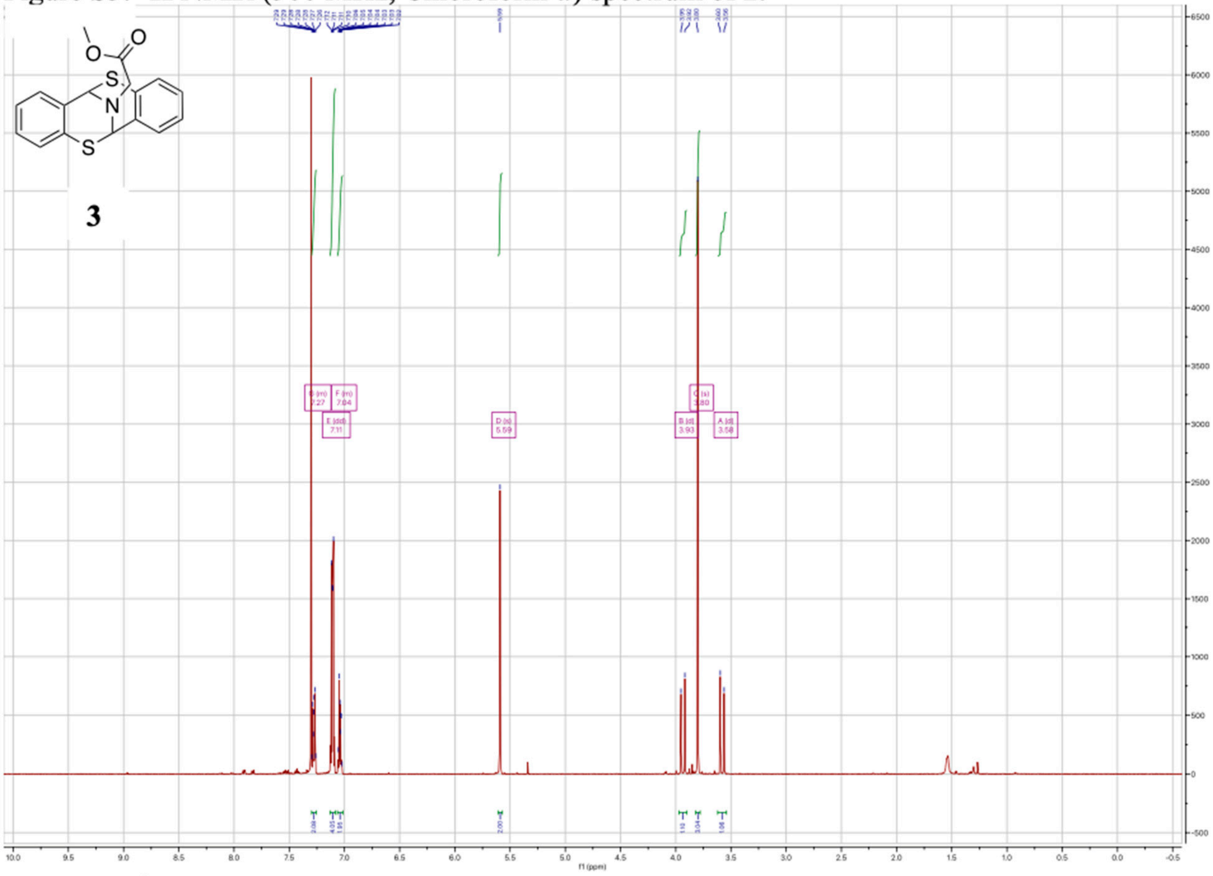

Figure S4: **^{1}H NMR** (500 MHz, Chloroform-*d*) spectrum of **3.**

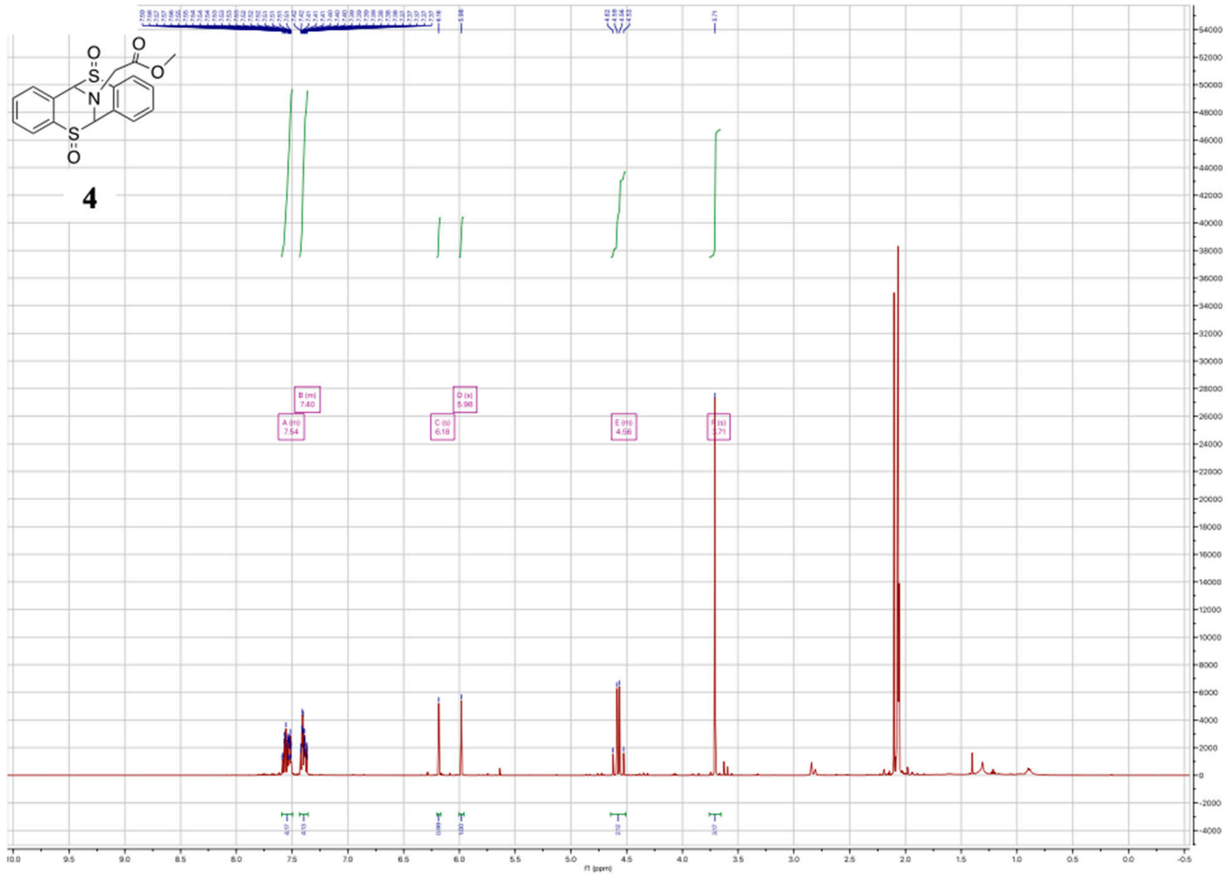

Figure S5: **^{1}H NMR** (500 MHz, Acetone-d_6) spectrum of **4.**

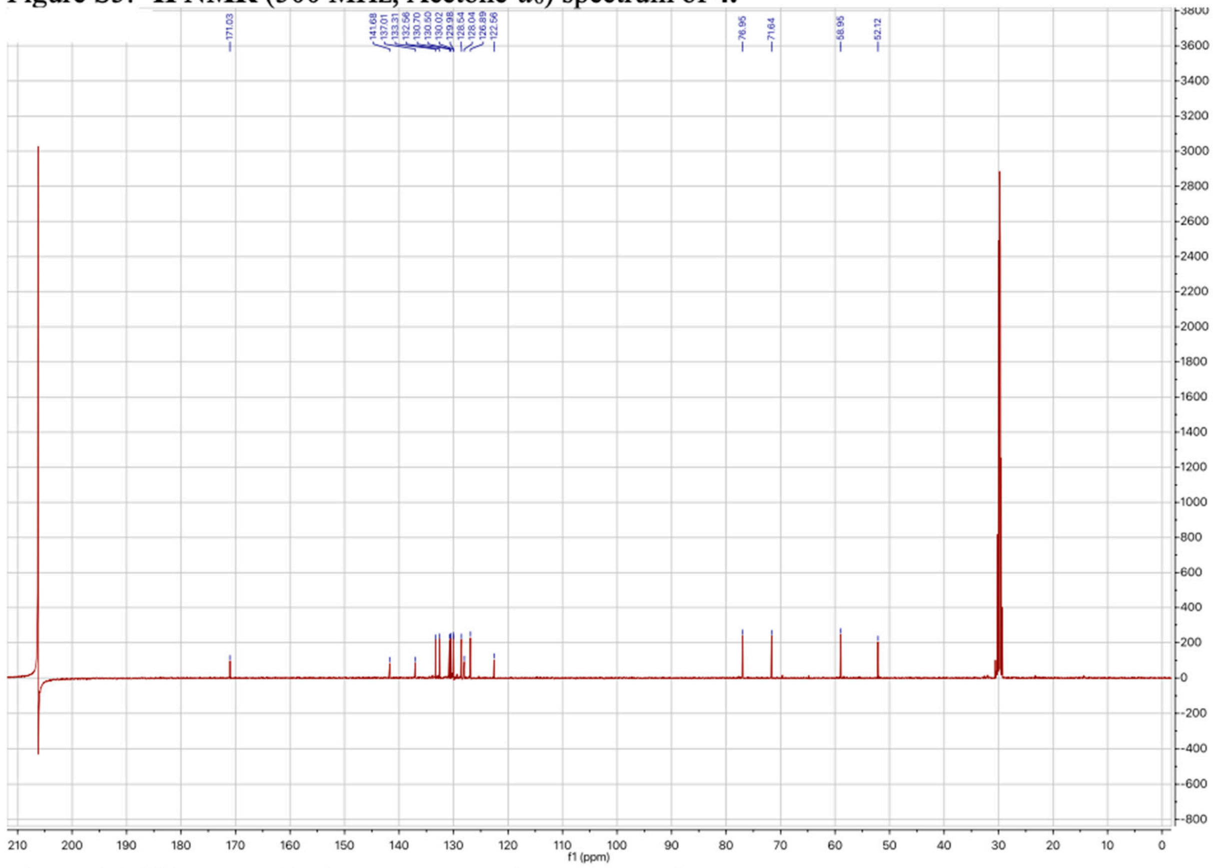

Figure S6: **^{13}C NMR** (126 MHz, Acetone) spectrum of **4.**

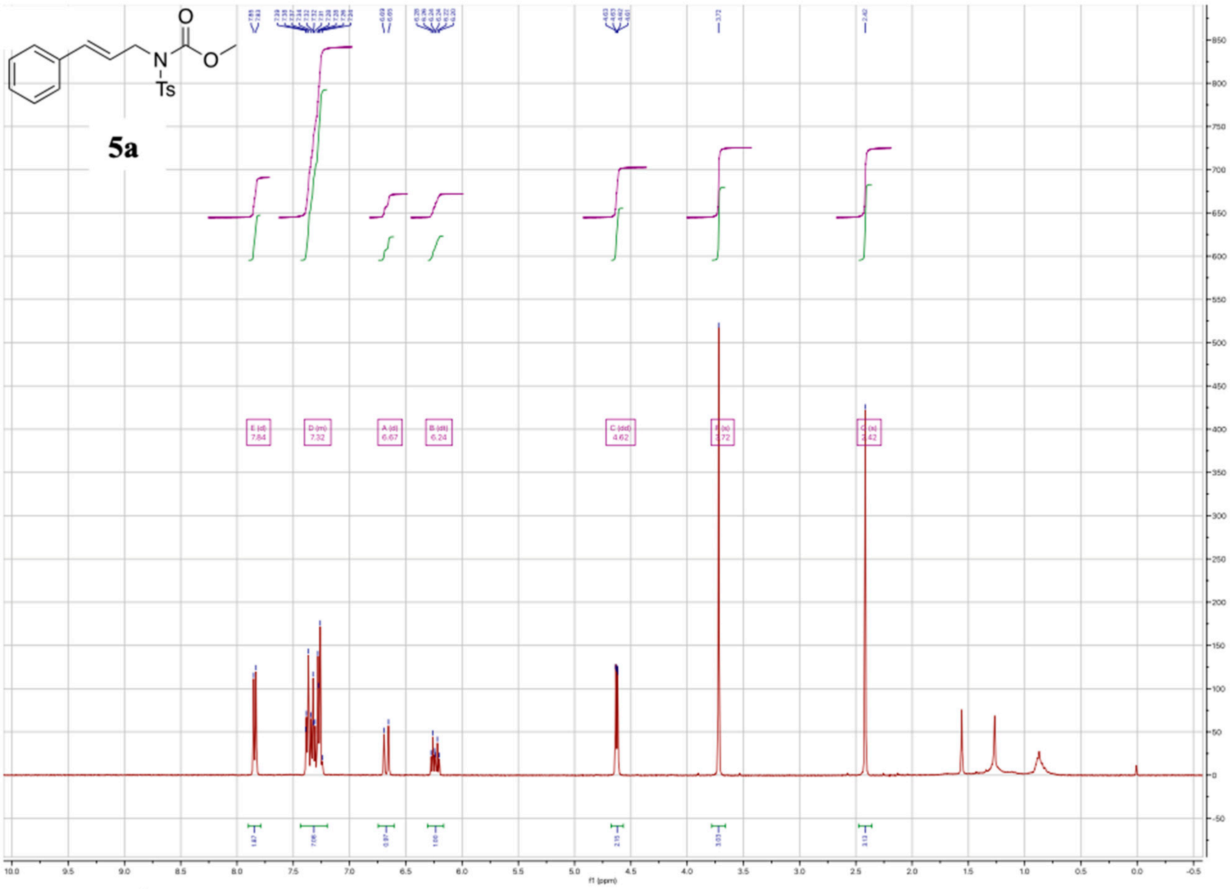

Figure S7: **^{1}H NMR** (500 MHz, Chloroform-*d*) spectrum of **5a**

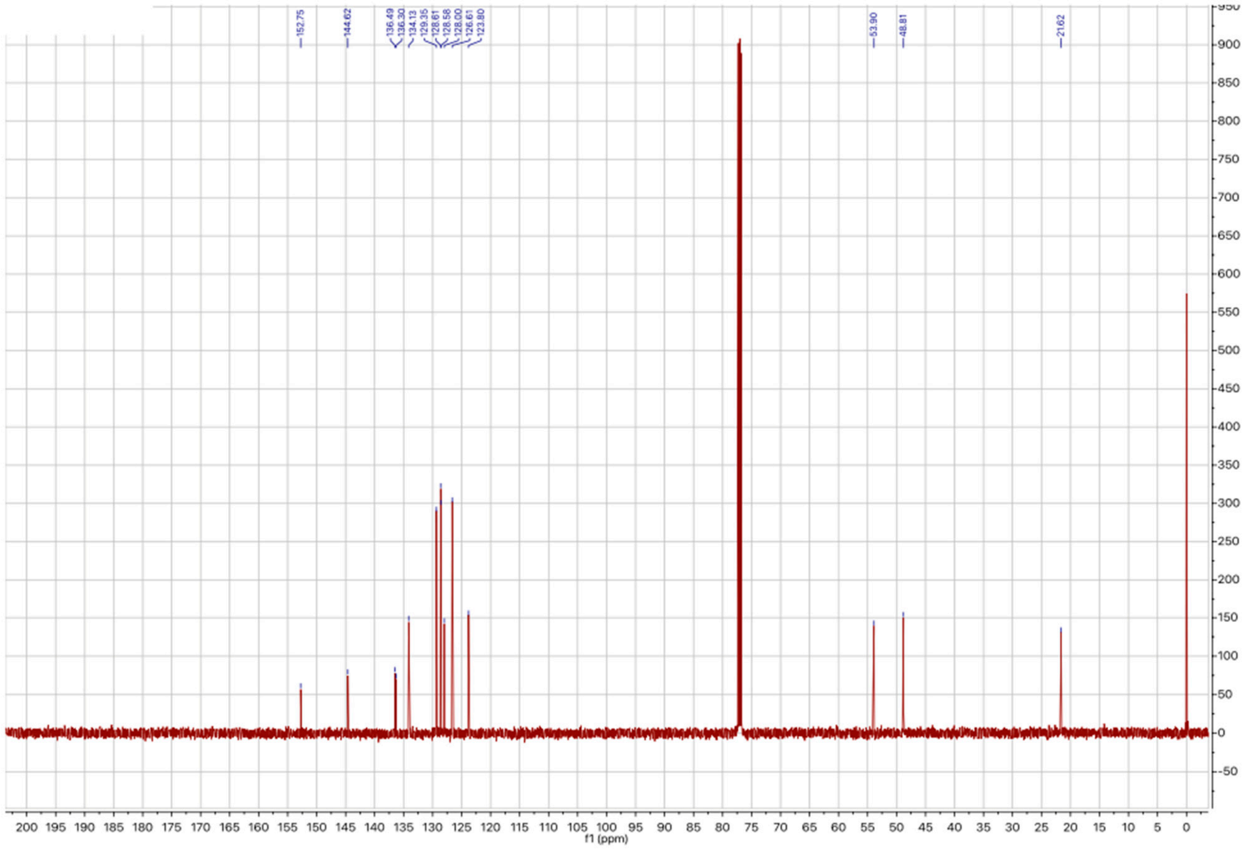

Figure S8: **^{13}C NMR** (126 MHz, $CDCl_3$) spectrum of **5a**

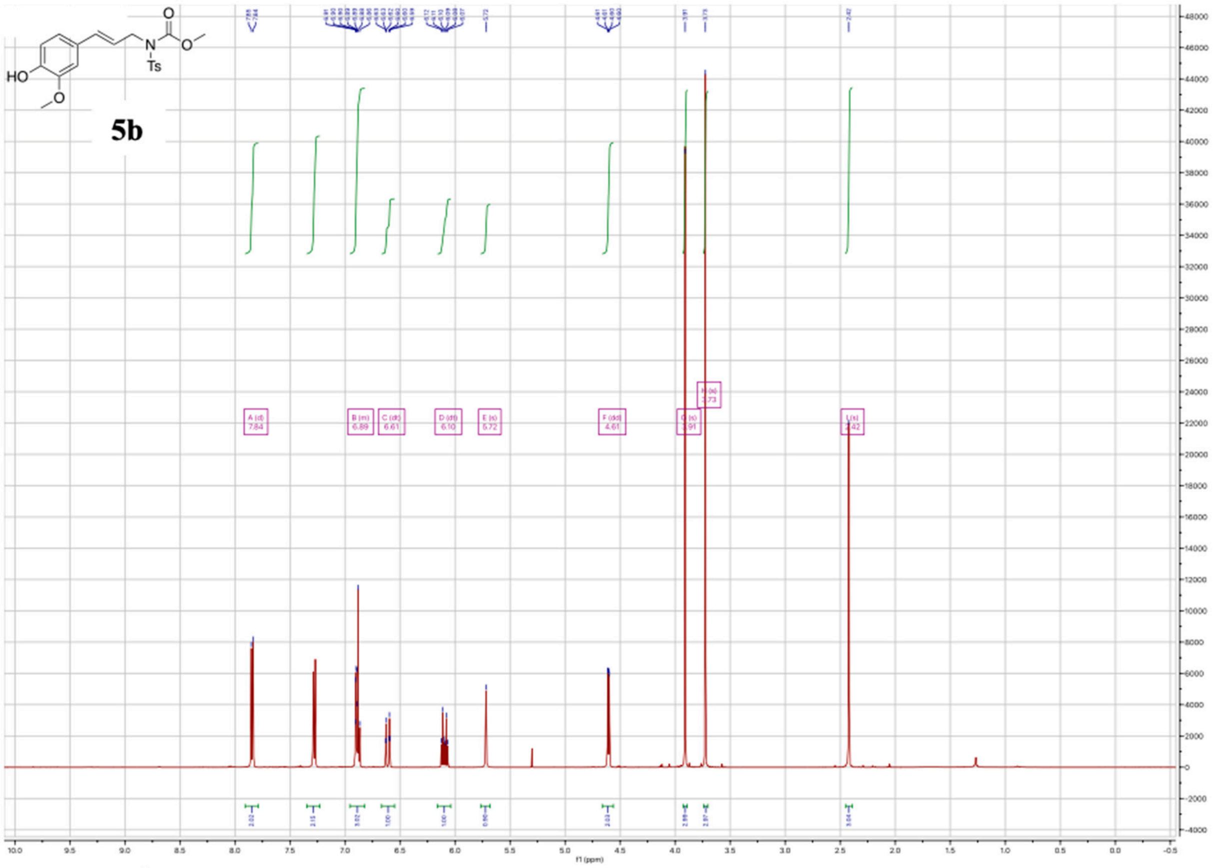

Figure S9: ^{1}**H NMR** (500 MHz, Chloroform-*d*) spectrum of **5b.**

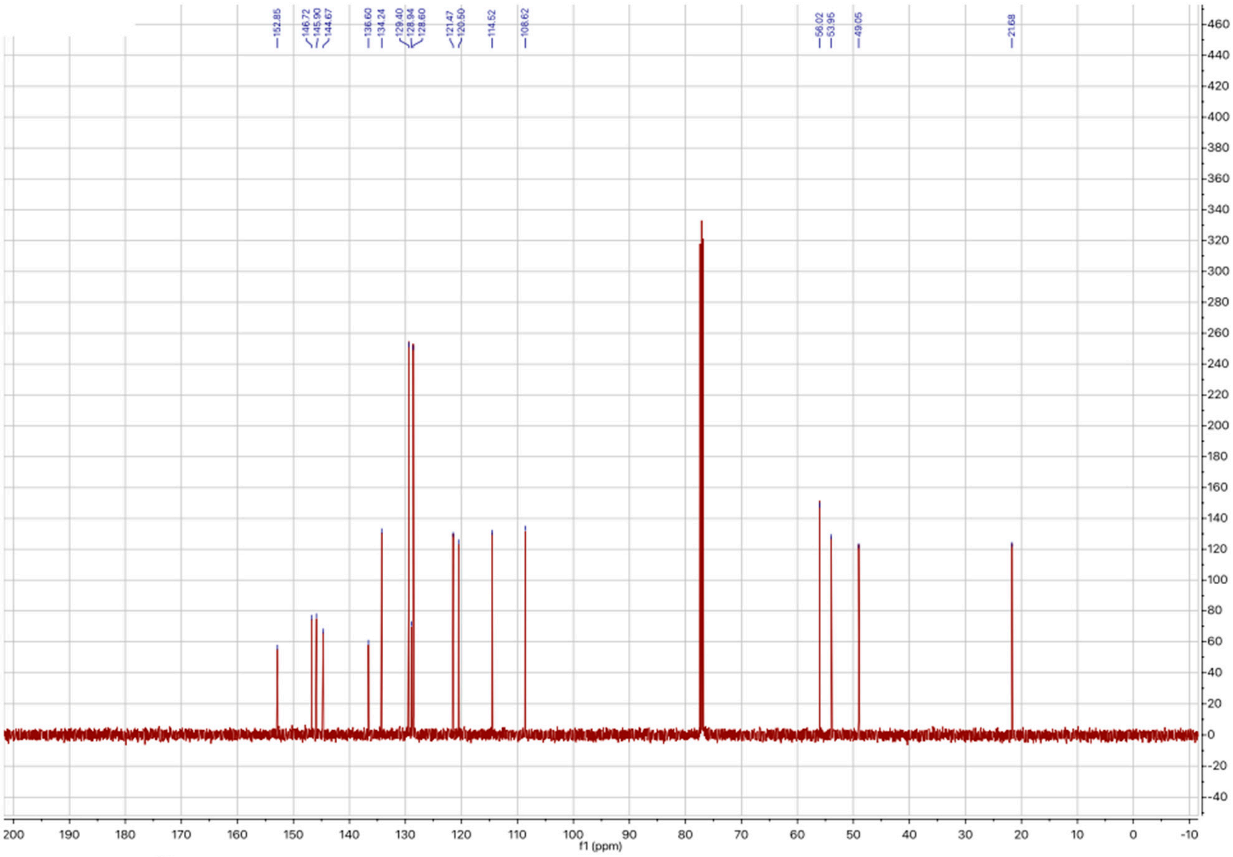

Figure S10: ^{13}**C NMR** (126 MHz, $CDCl_3$) spectrum of **5b.**

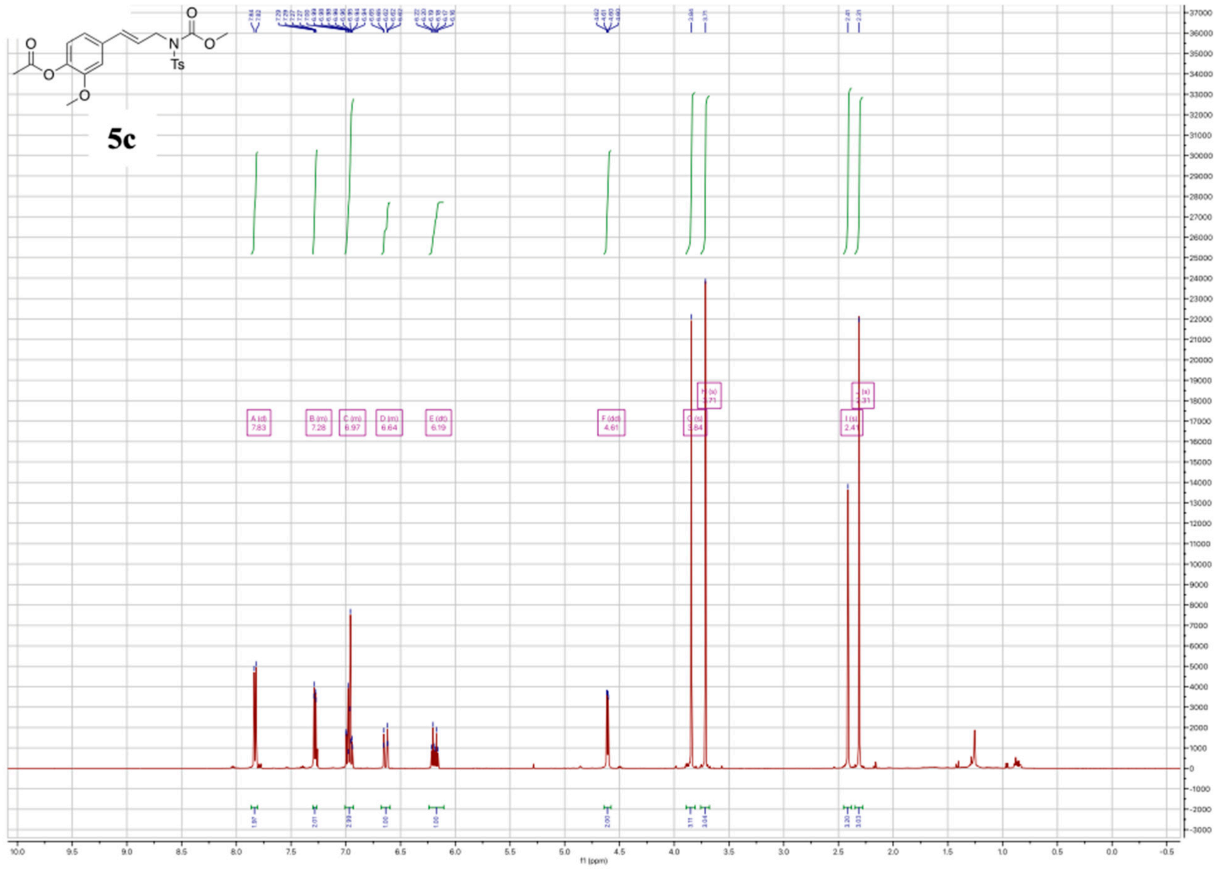

Figure S11: **^{1}H NMR** (500 MHz, Chloroform-*d*) spectrum of **5c.**

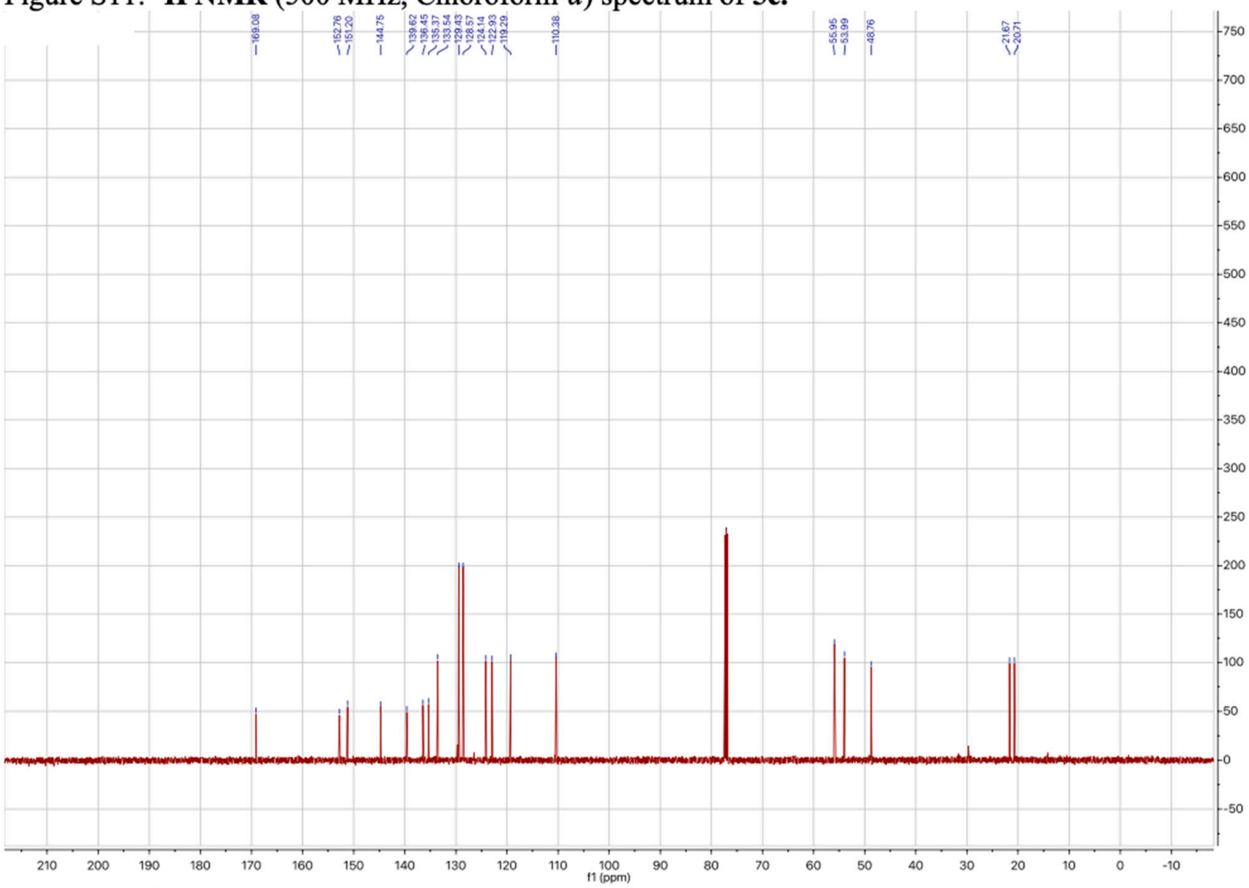

Figure S12: **^{13}C NMR** (126 MHz, $CDCl_3$) spectrum of **5c.**

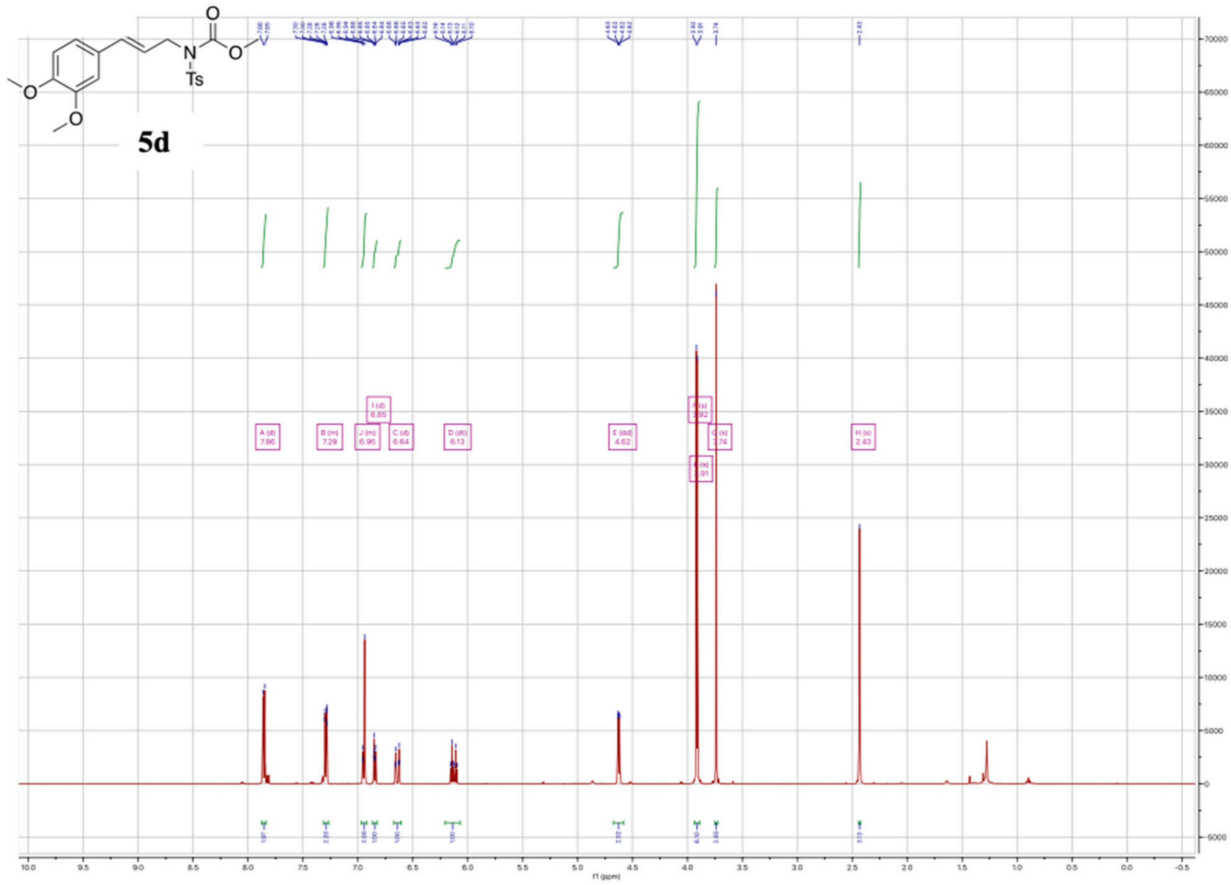

Figure S13: **^{1}H NMR** (500 MHz, Chloroform-*d*) spectrum of **5d.**

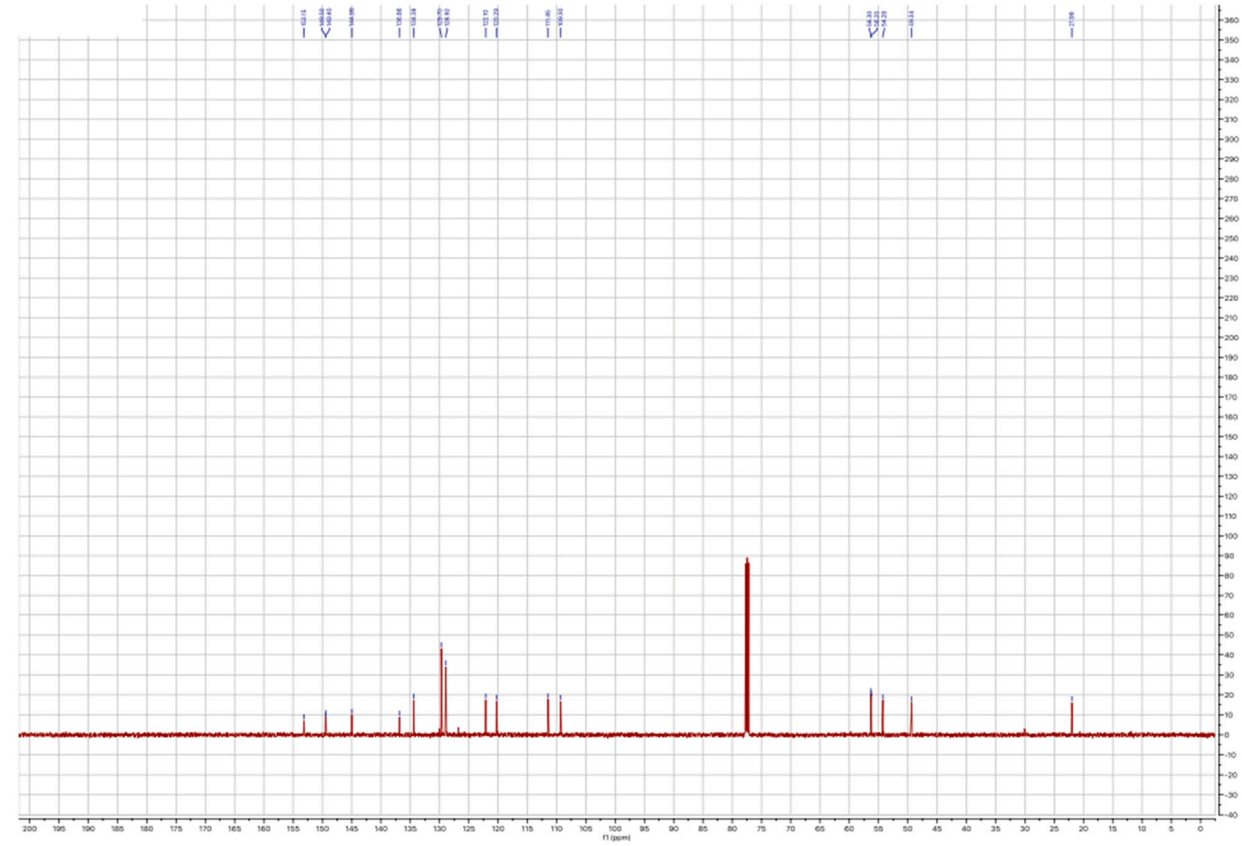

Figure S14: **^{13}C NMR** (126 MHz, $CDCl_3$) spectrum of **5d.**

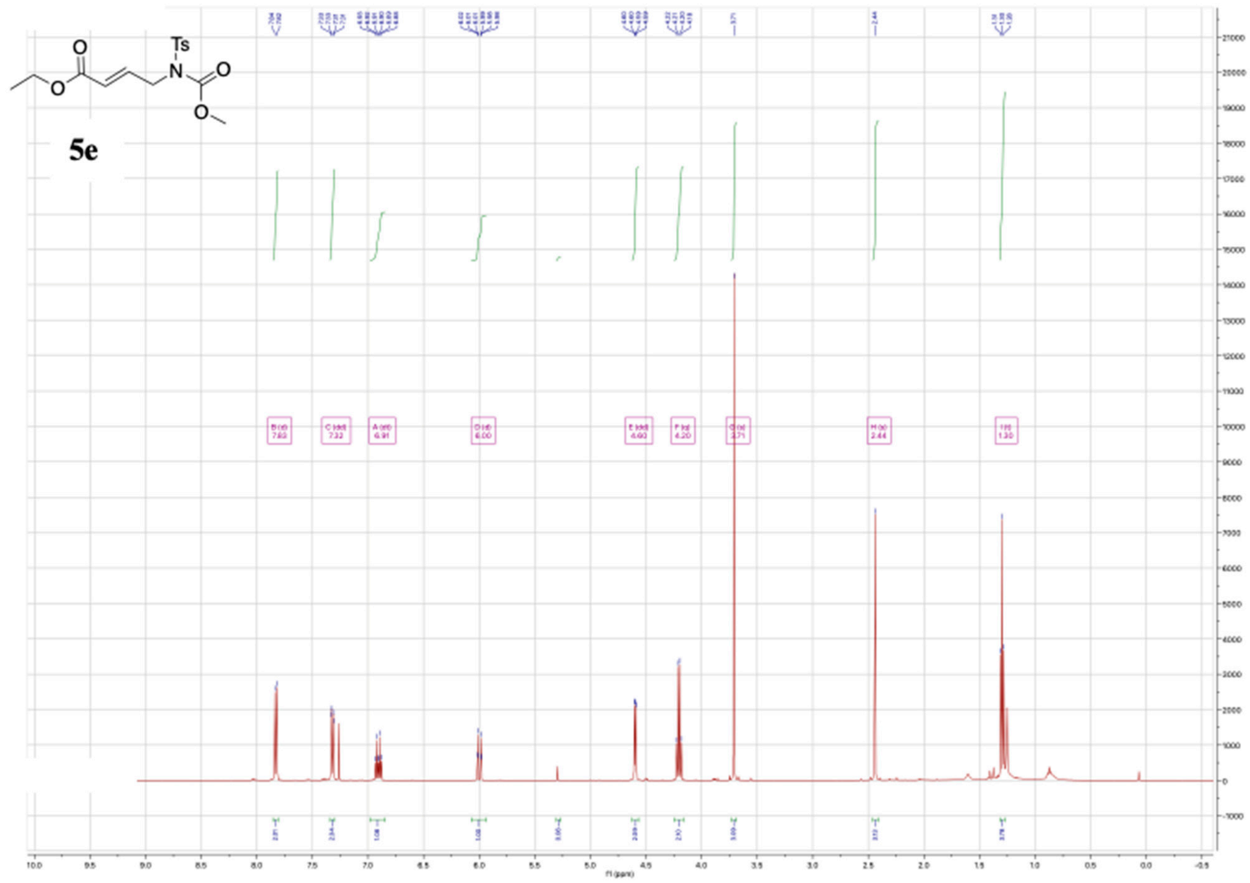

Figure S55: **^{1}H NMR** (500 MHz, Chloroform-*d*) spectrum of **5e.**

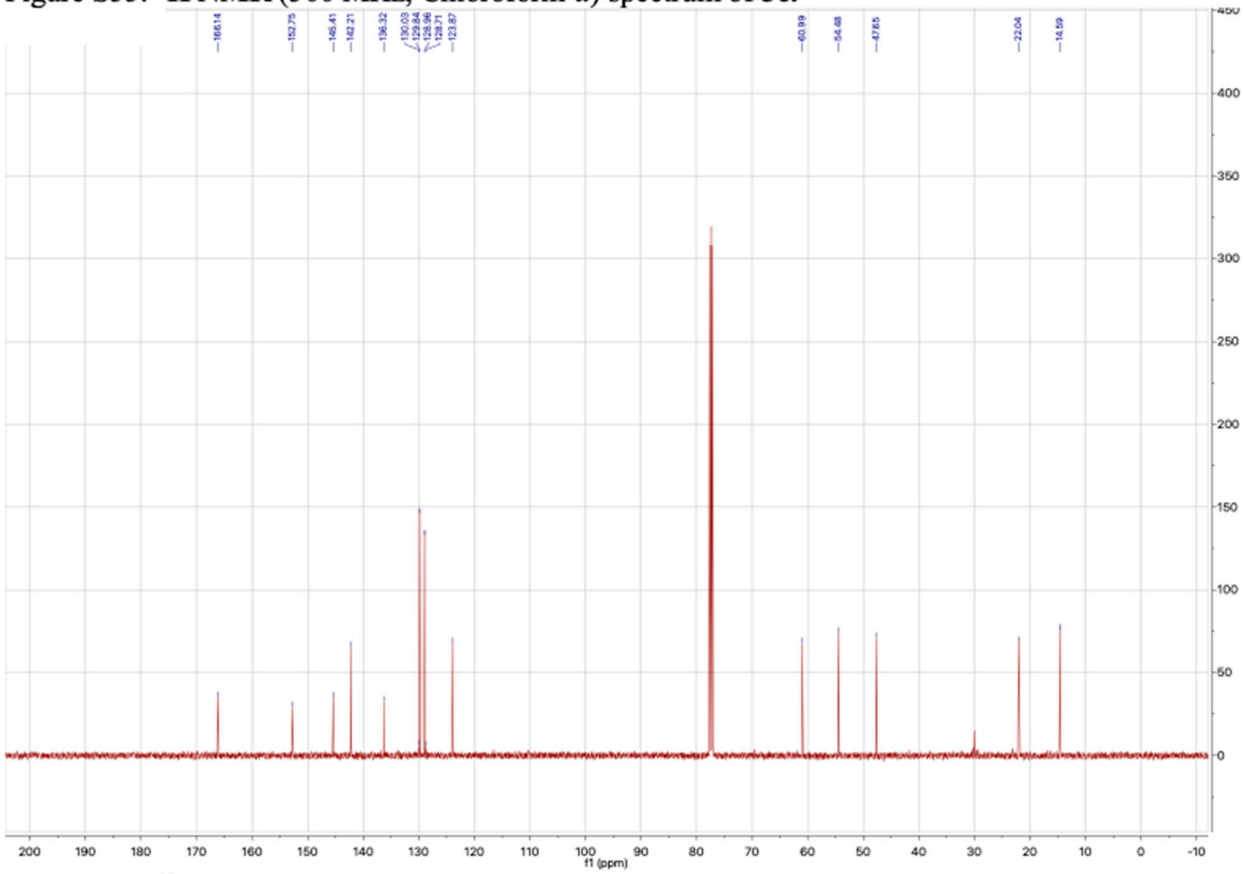

Figure S56: **^{13}C NMR** (126 MHz, $CDCl_3$) spectrum of **5e.**

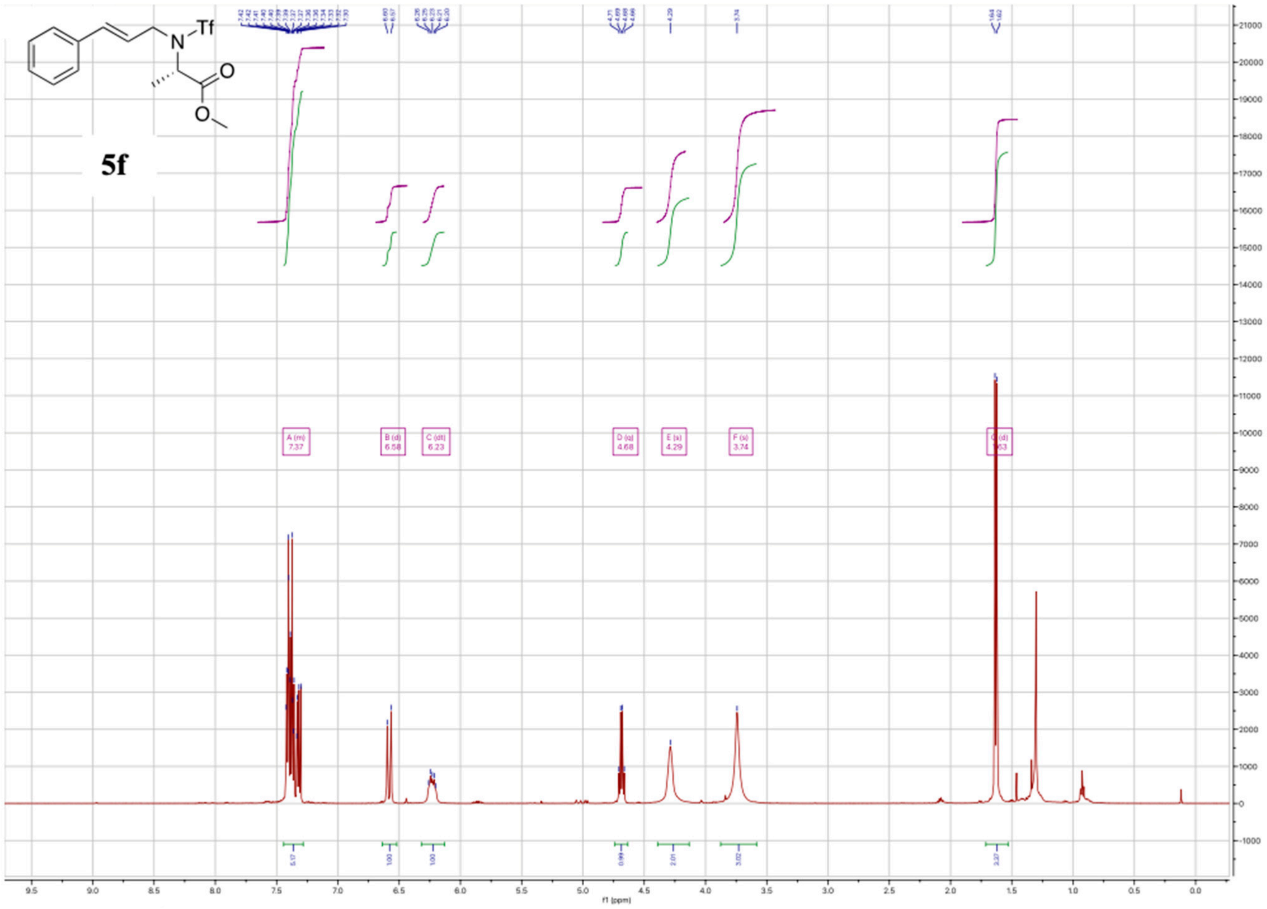

Figure S15: **^{1}H NMR** (500 MHz, Chloroform-*d*) spectrum of **5f.**

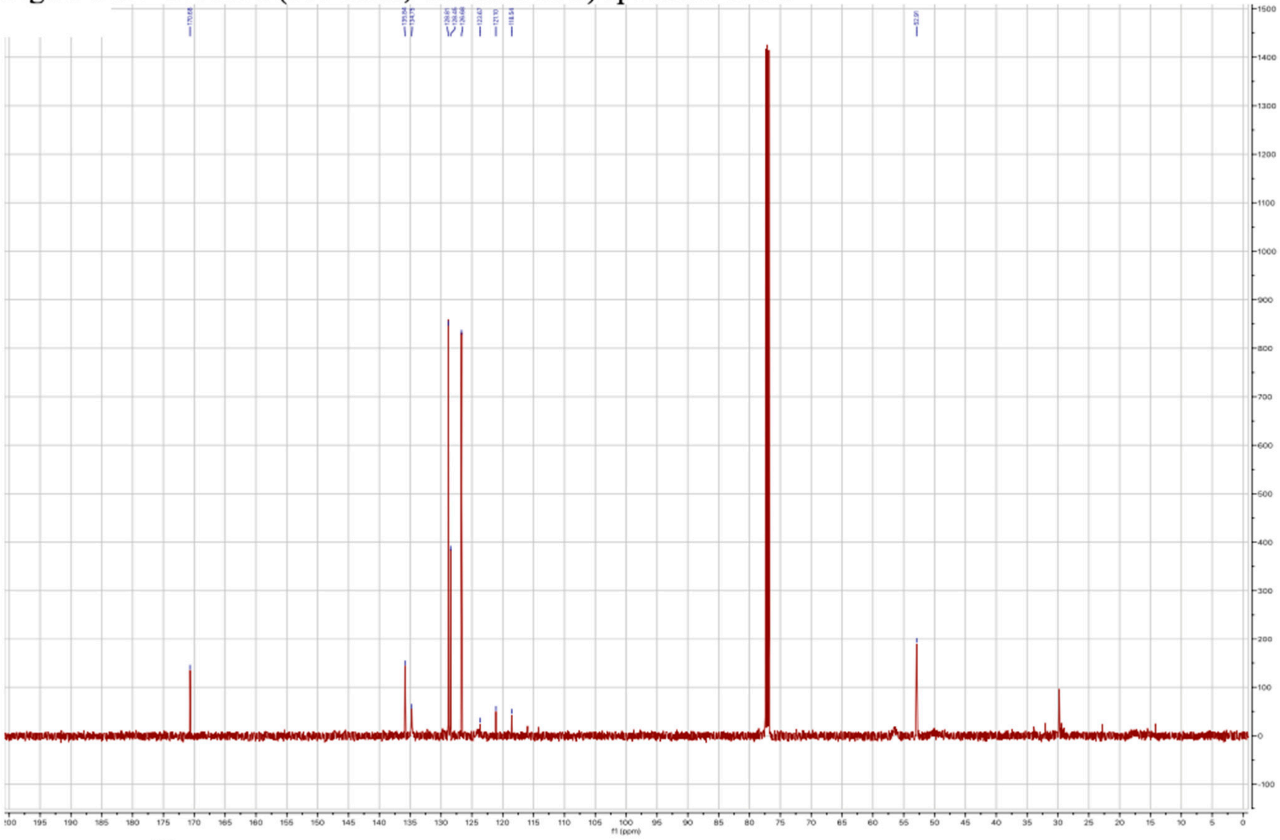

Figure S16: **^{13}C NMR** (126 MHz, $CDCl_3$) spectrum of **5f.**

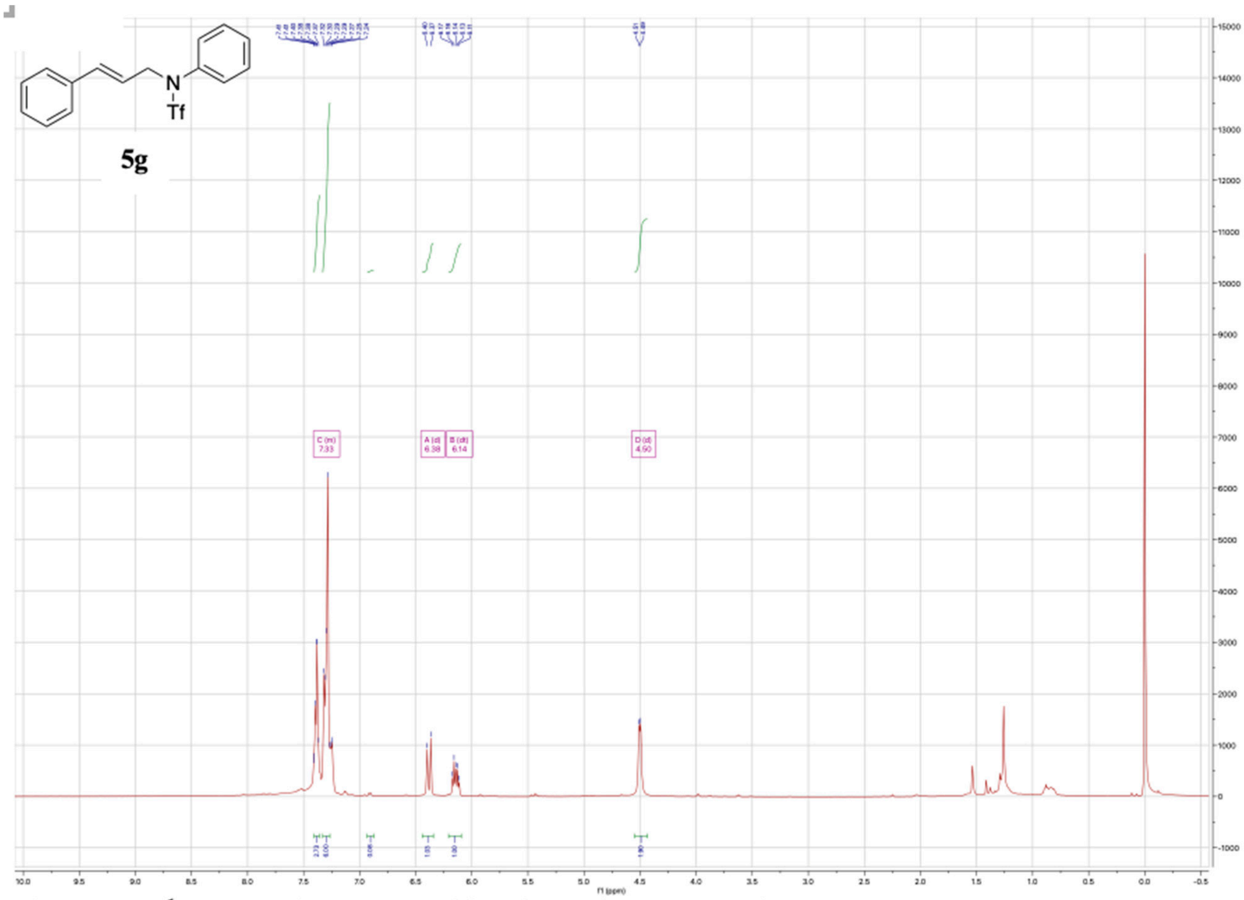

Figure S17: **^{1}H NMR** (500 MHz, Chloroform-*d*) spectrum of **5g.**

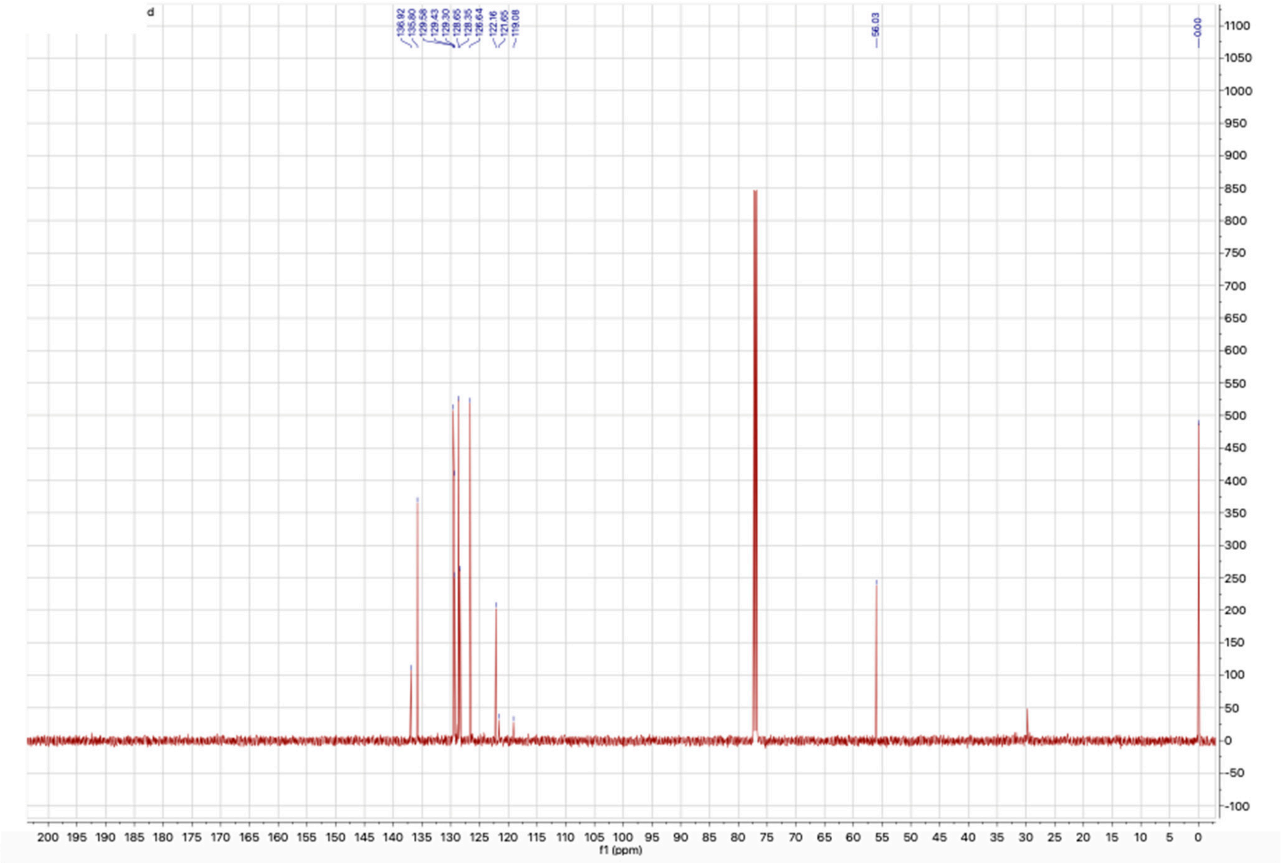

Figure S18: **^{13}C NMR** (126 MHz, $CDCl_3$) spectrum of **5g.**

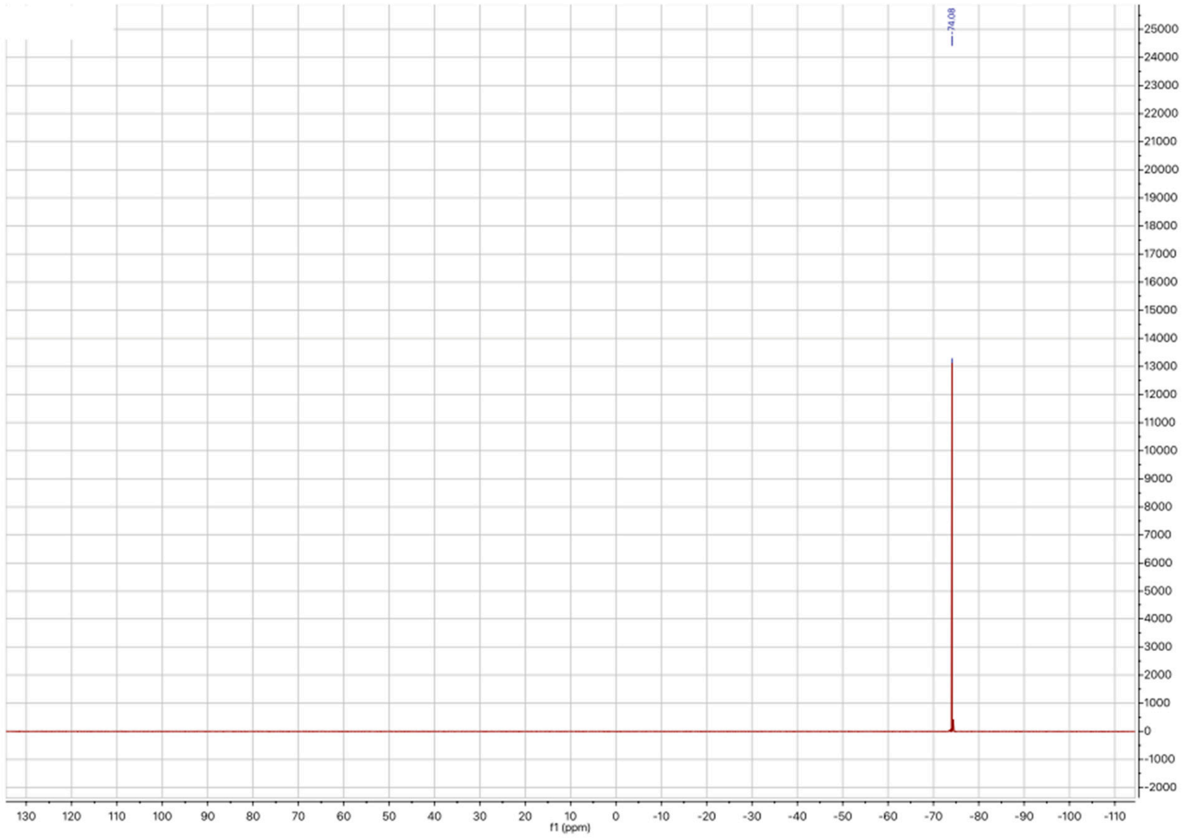

Figure S19: **^{19}F NMR** (471 MHz, $CDCl_3$) spectrum of **5g.**

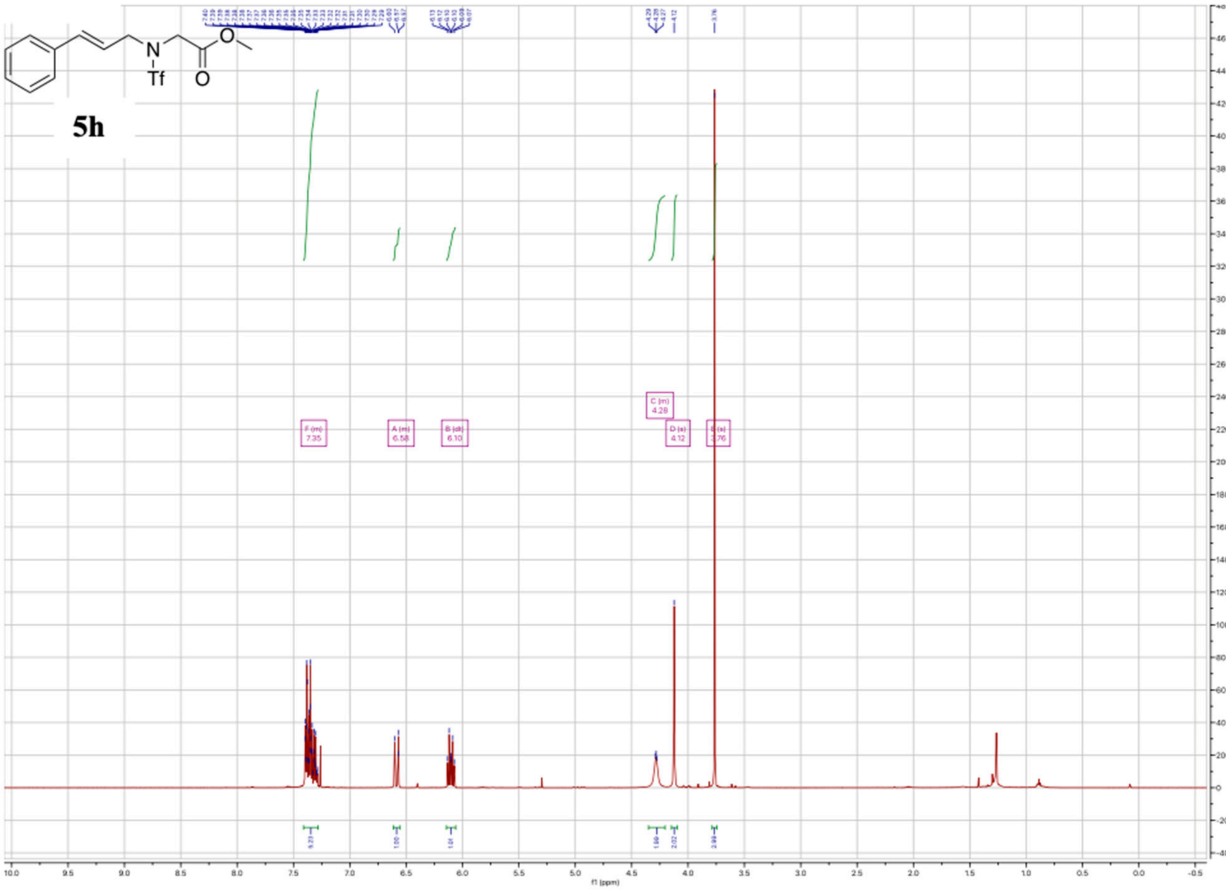

Figure S20: **^{1}H NMR** (500 MHz, Chloroform-*d*) spectrum of **5h.**

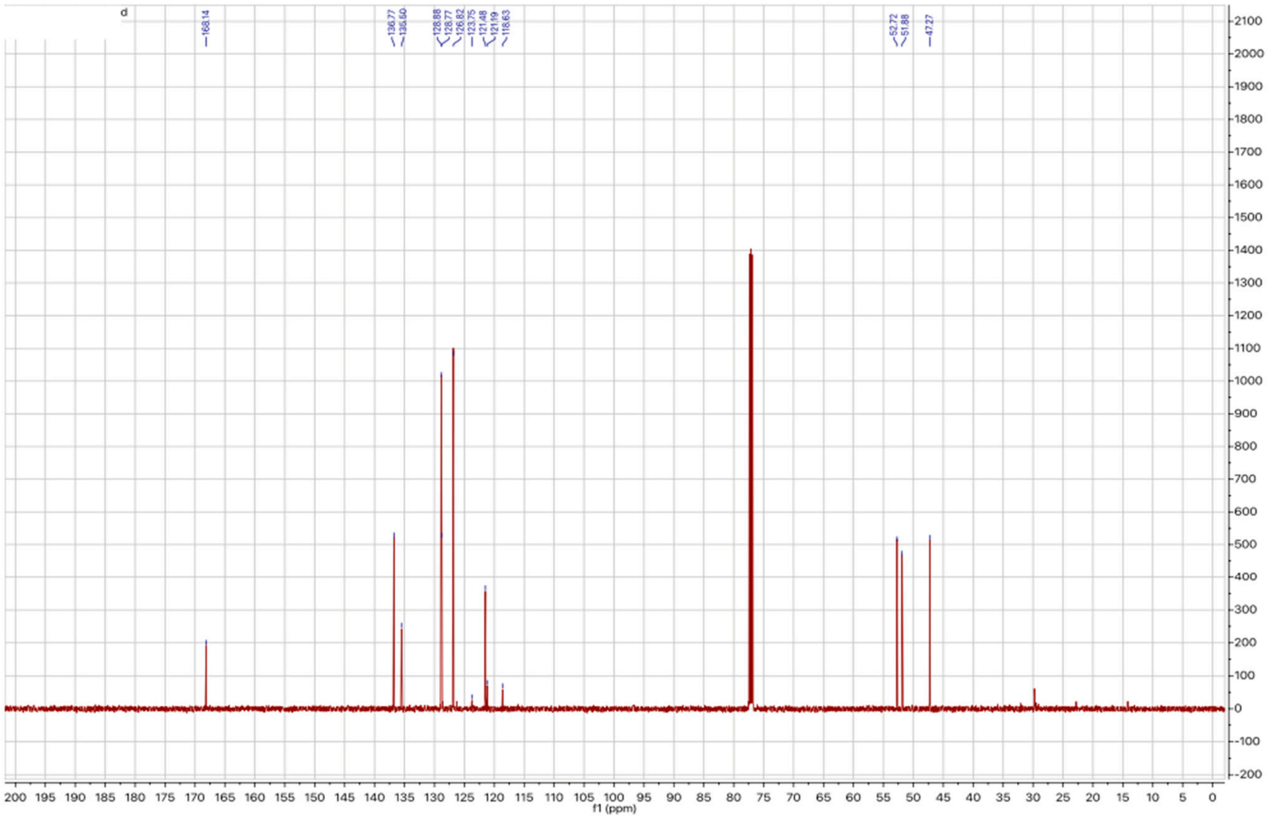

Figure S21: **^{13}C NMR** (126 MHz, $CDCl_3$) spectrum of **5h.**

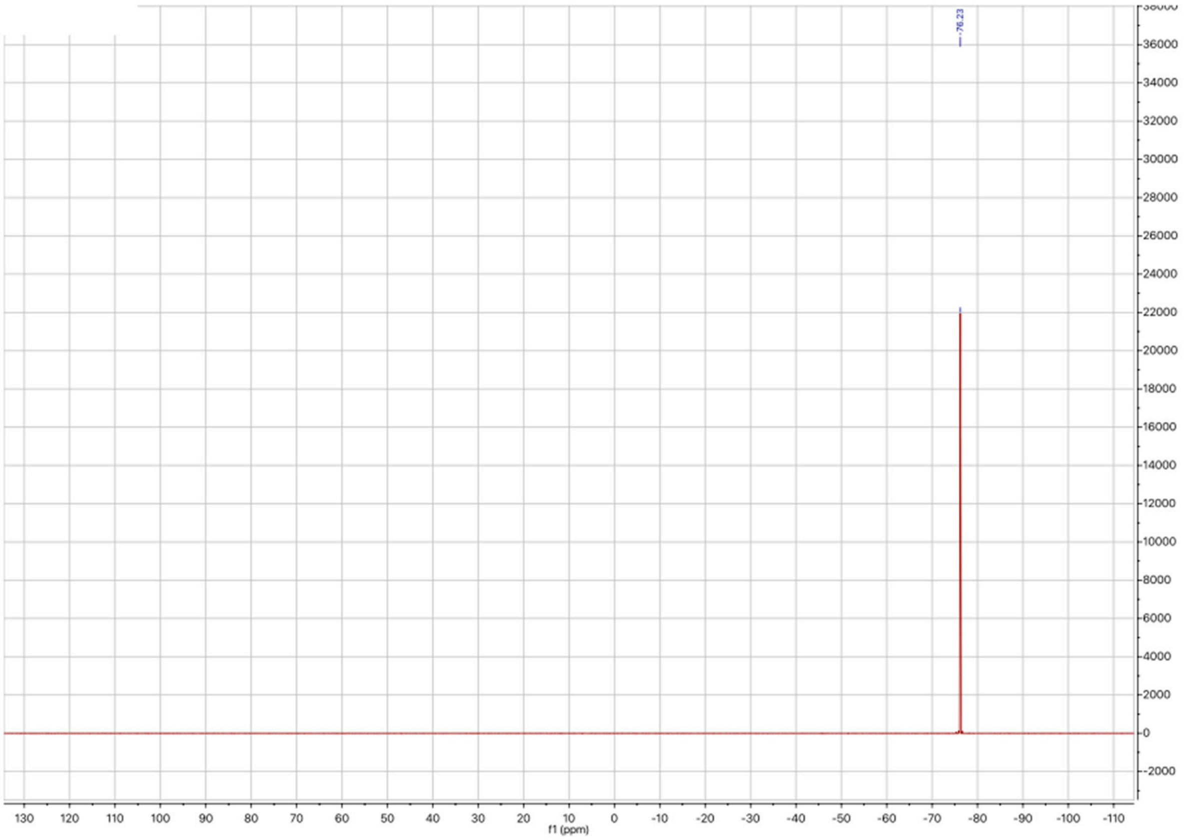

Figure S22: ^{19}F NMR (471 MHz, $CDCl_3$) spectrum of **5h.**

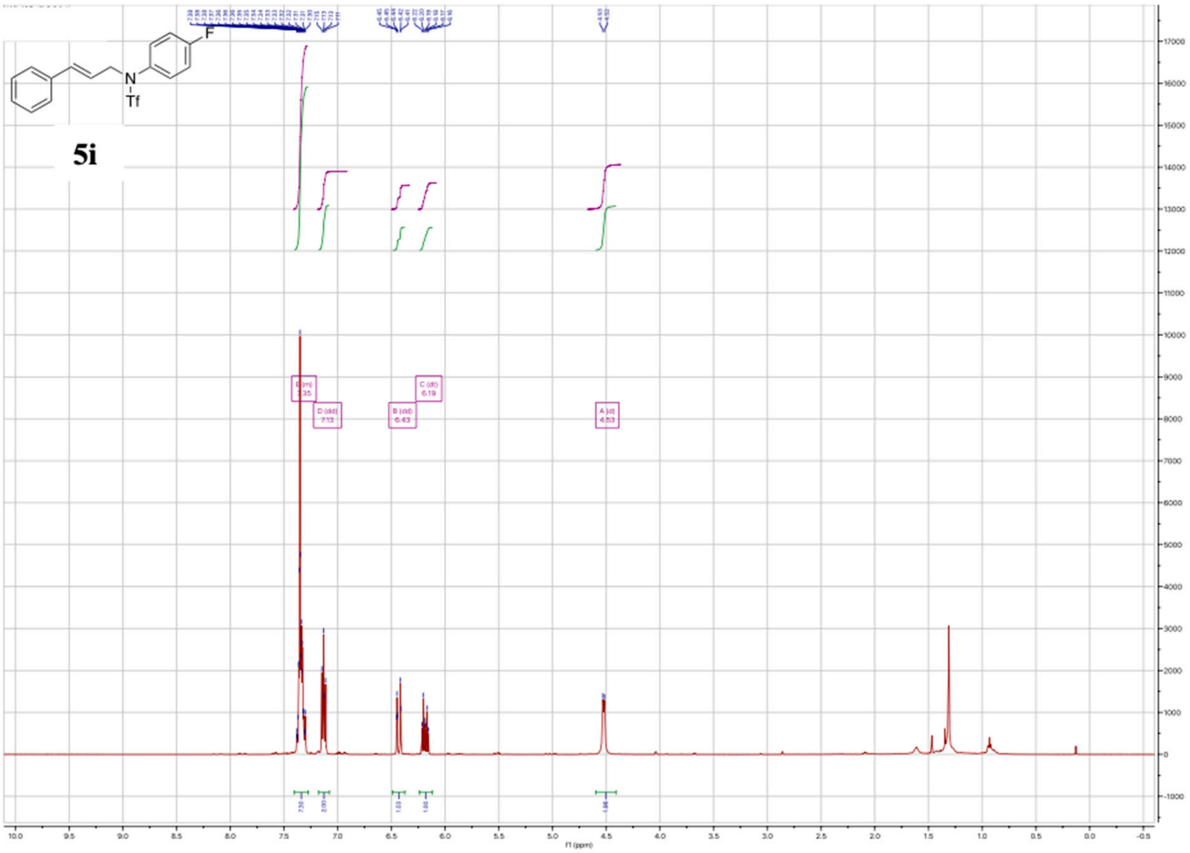

Figure S23: **^{1}H NMR** (500 MHz, Chloroform-*d*) spectrum of **5i.**

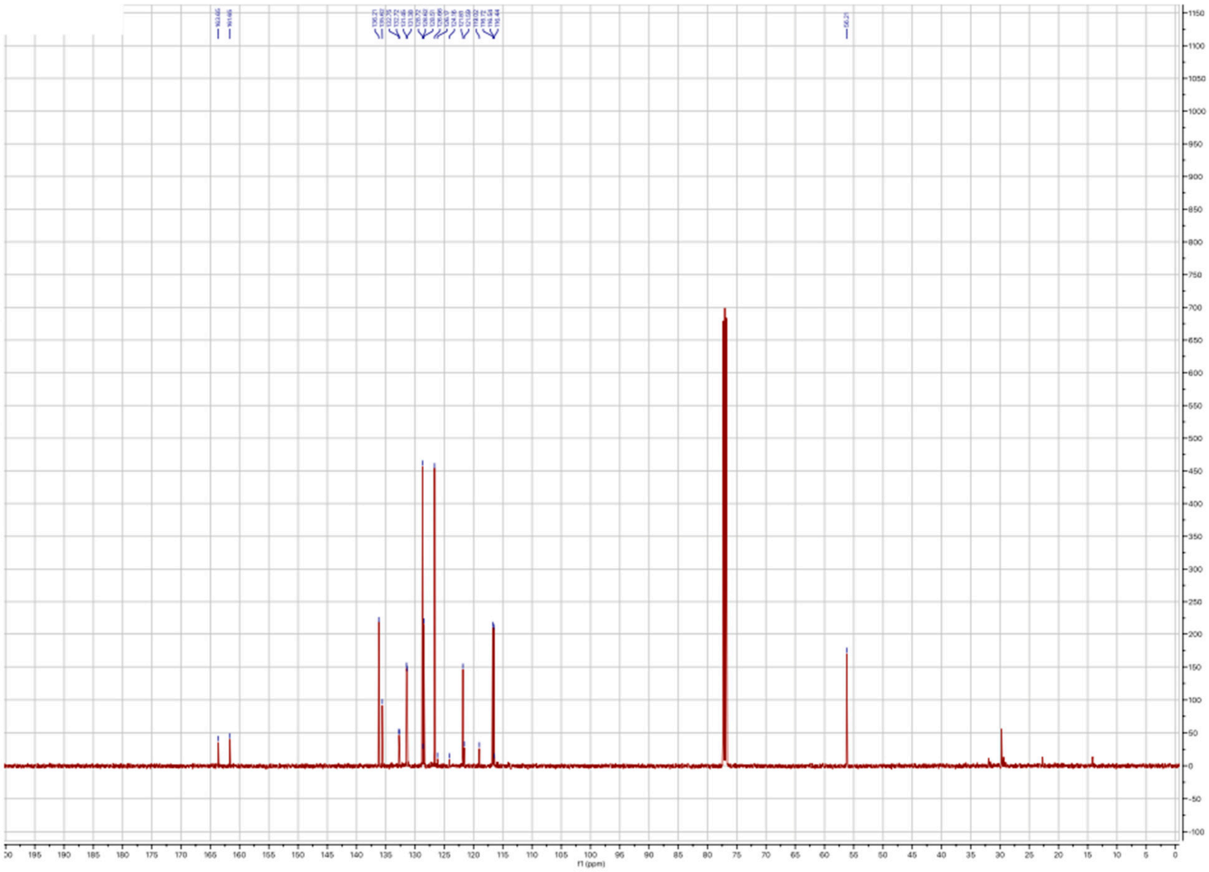

Figure S24: **^{13}C NMR** (126 MHz, $CDCl_3$) spectrum of **5i.**

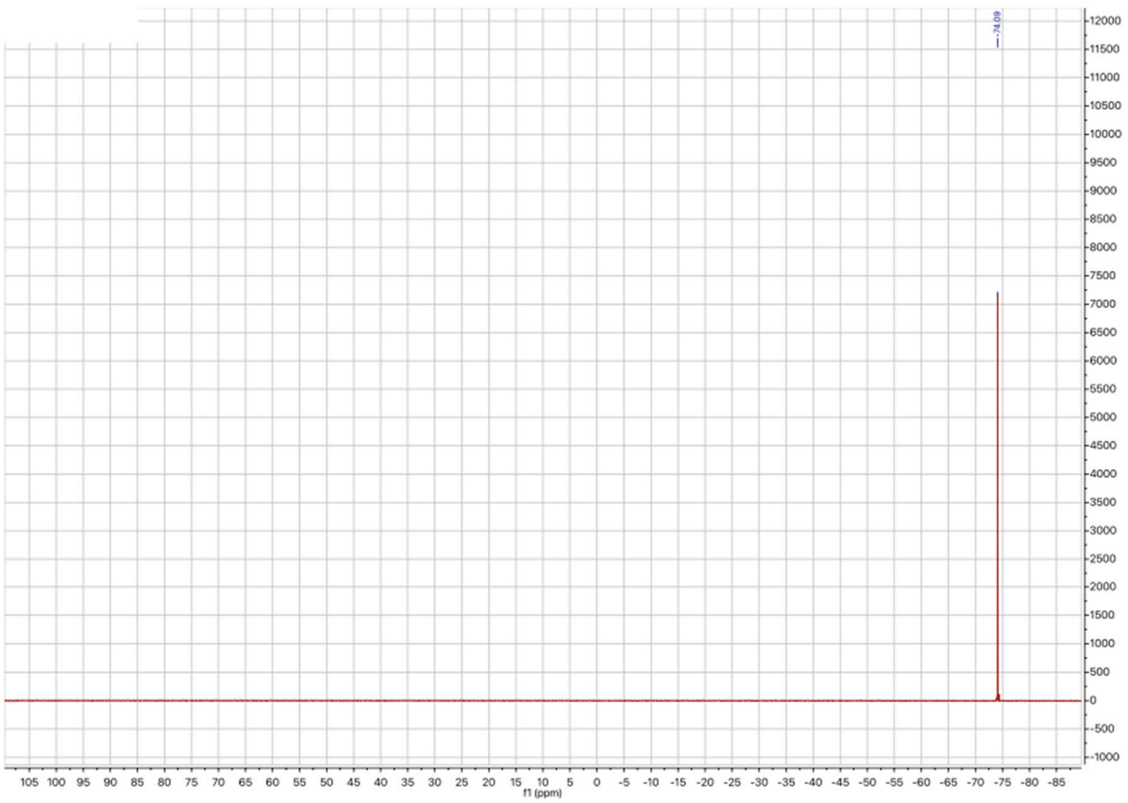

Figure S25: **^{19}F NMR** (471 MHz, $CDCl_3$) spectrum of **5i.**

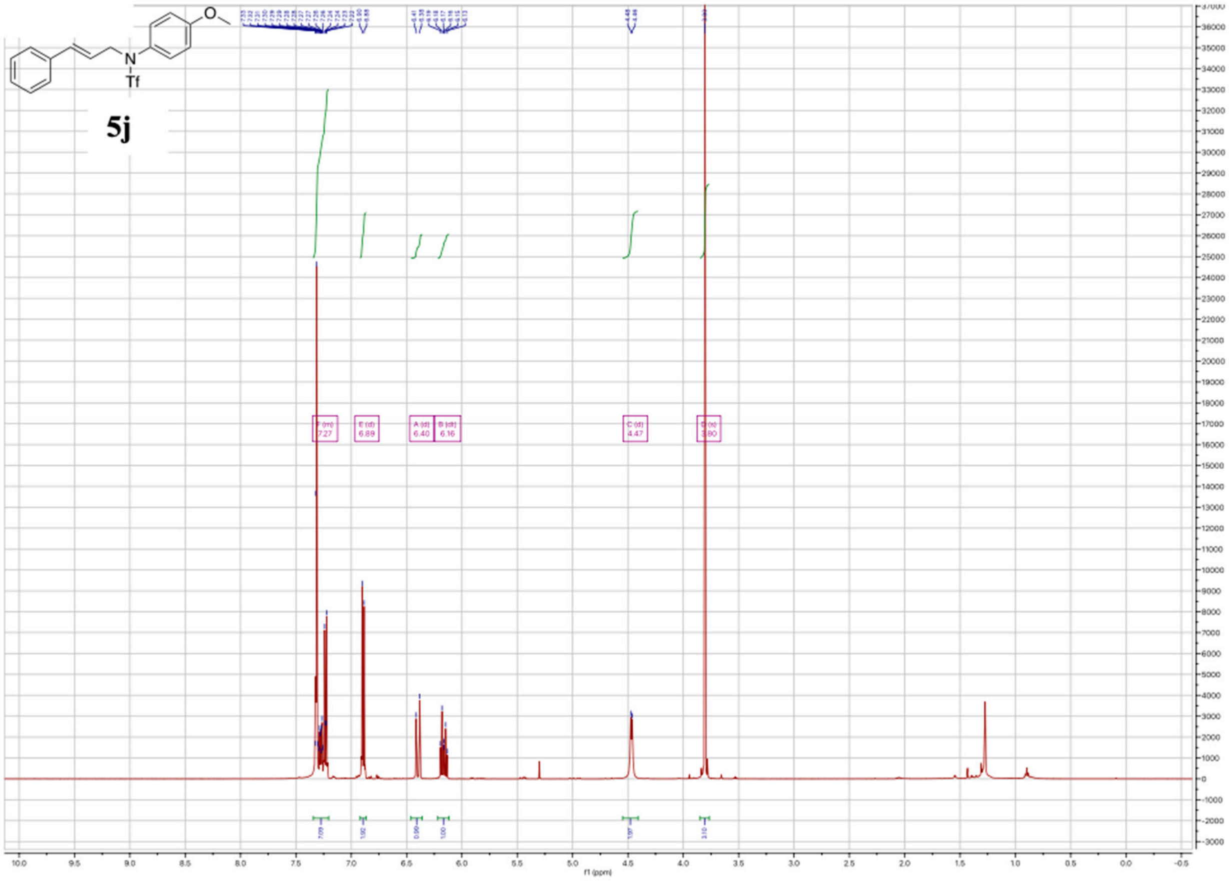

Figure S26: **^{1}H NMR** (500 MHz, Chloroform-*d*) spectrum of **5j.**

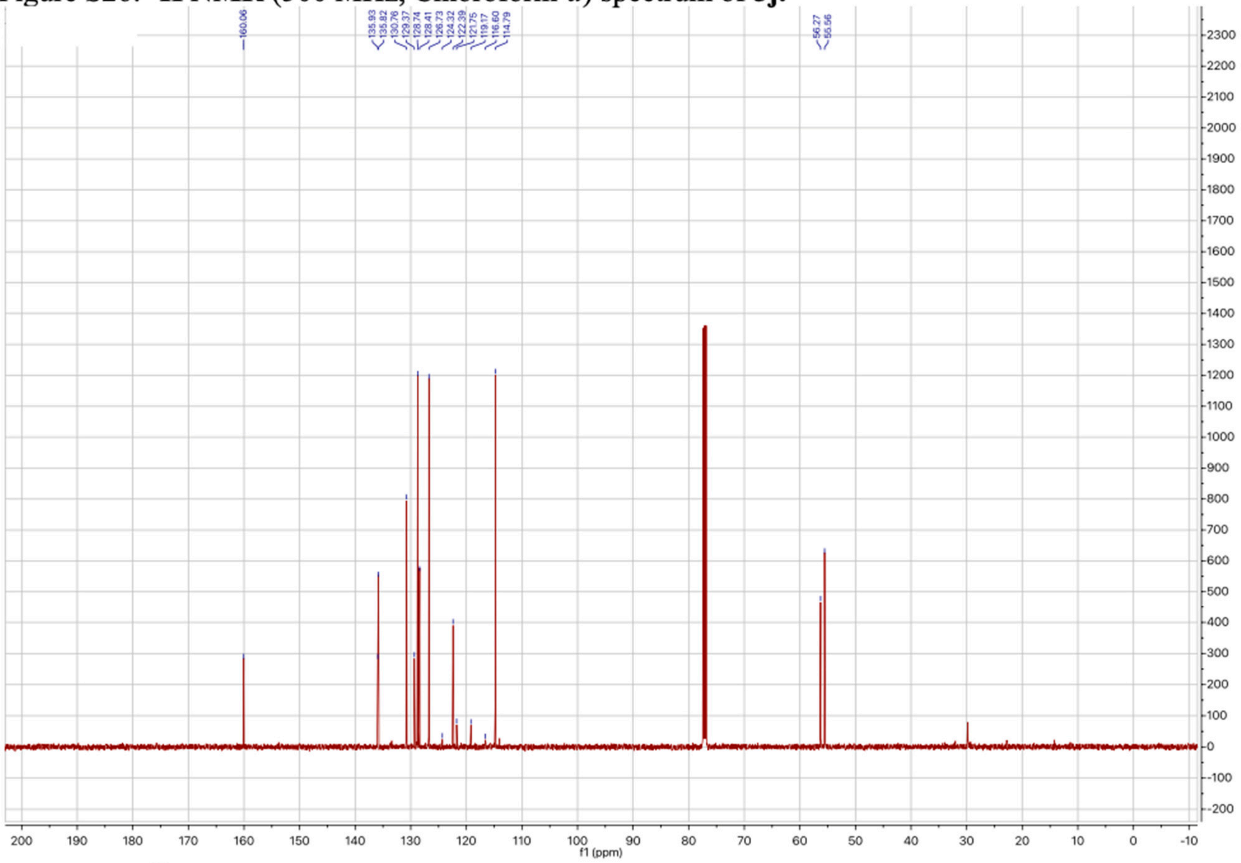

Figure S27: **^{13}C NMR** (126 MHz, $CDCl_3$) spectrum of **5j.**

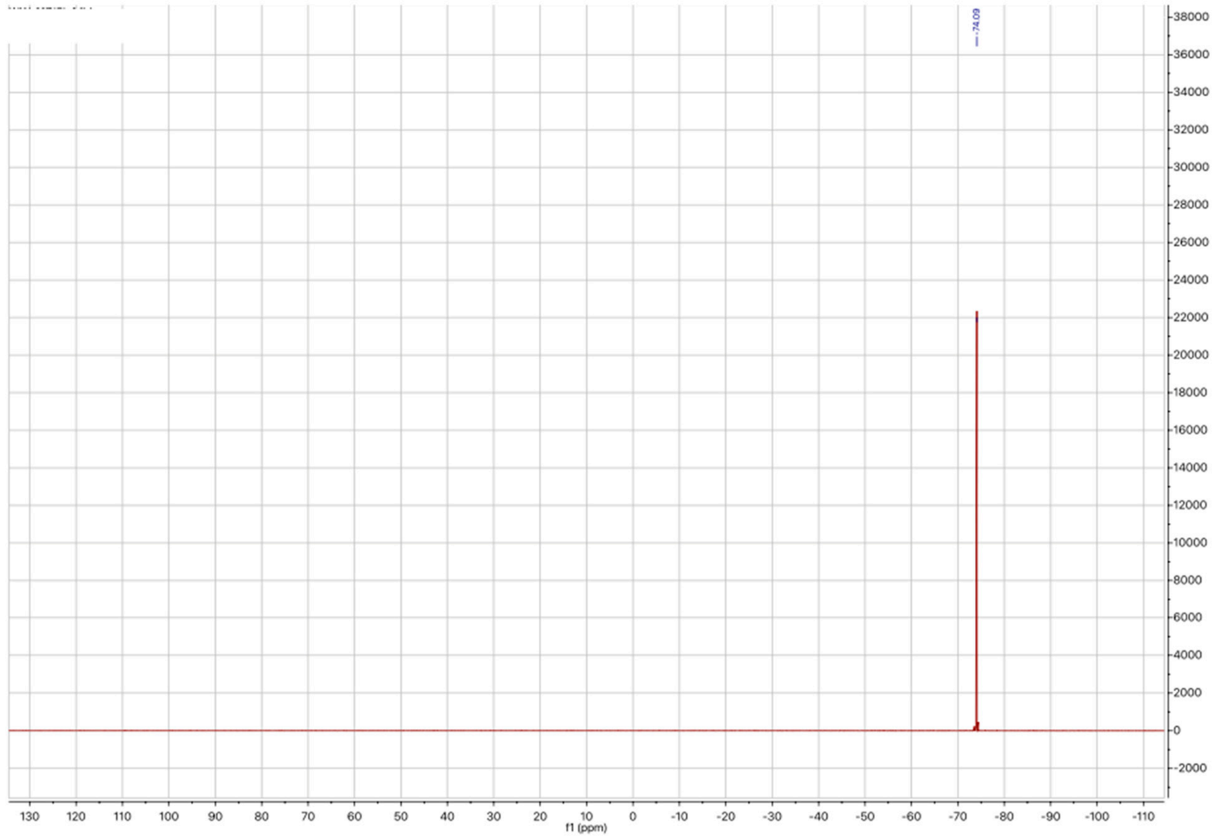

Figure S28: ^{19}**F NMR** (471 MHz, $CDCl_3$) spectrum of **5j.**

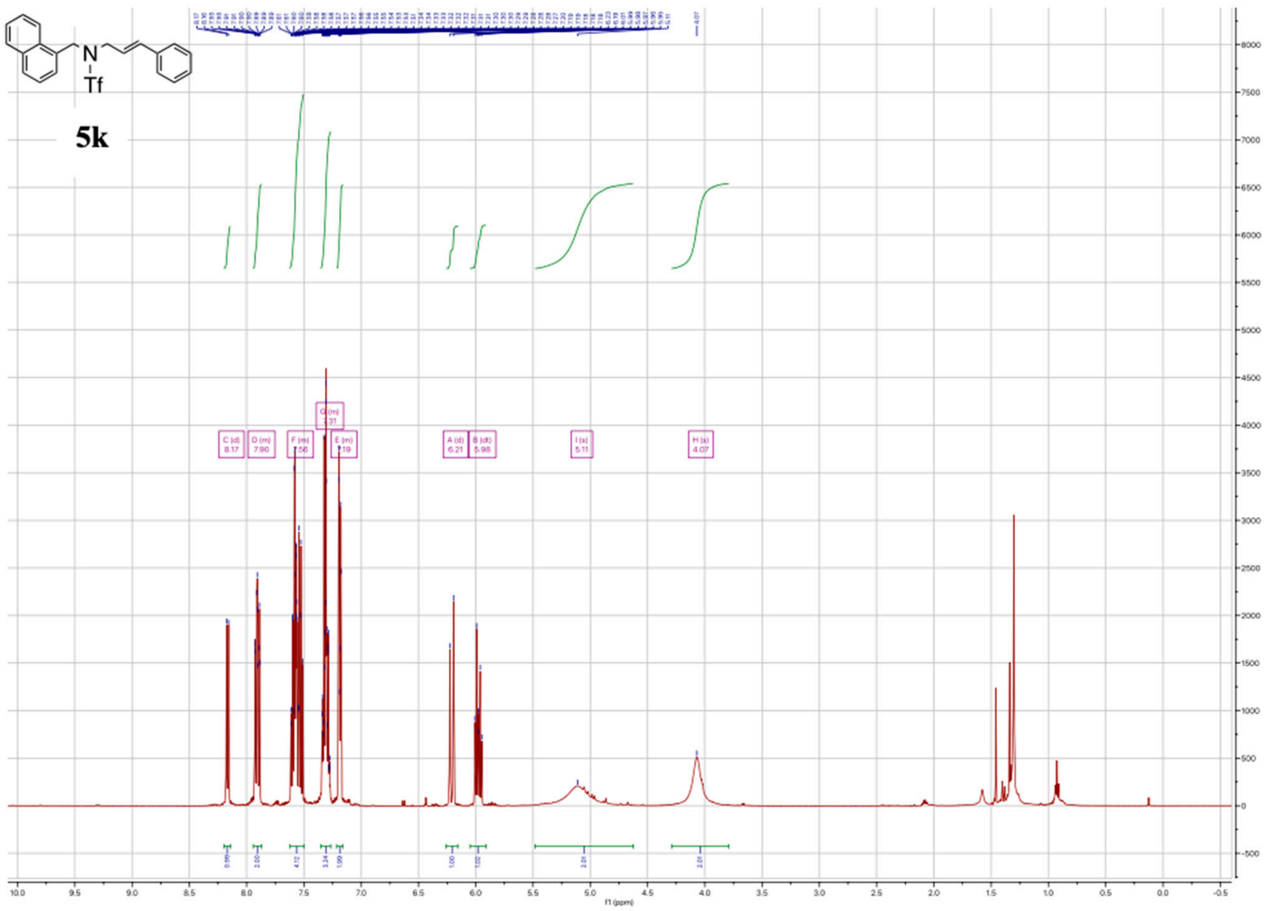

Figure S29: **^{1}H NMR** (500 MHz, Chloroform-*d*) spectrum of **5k.**

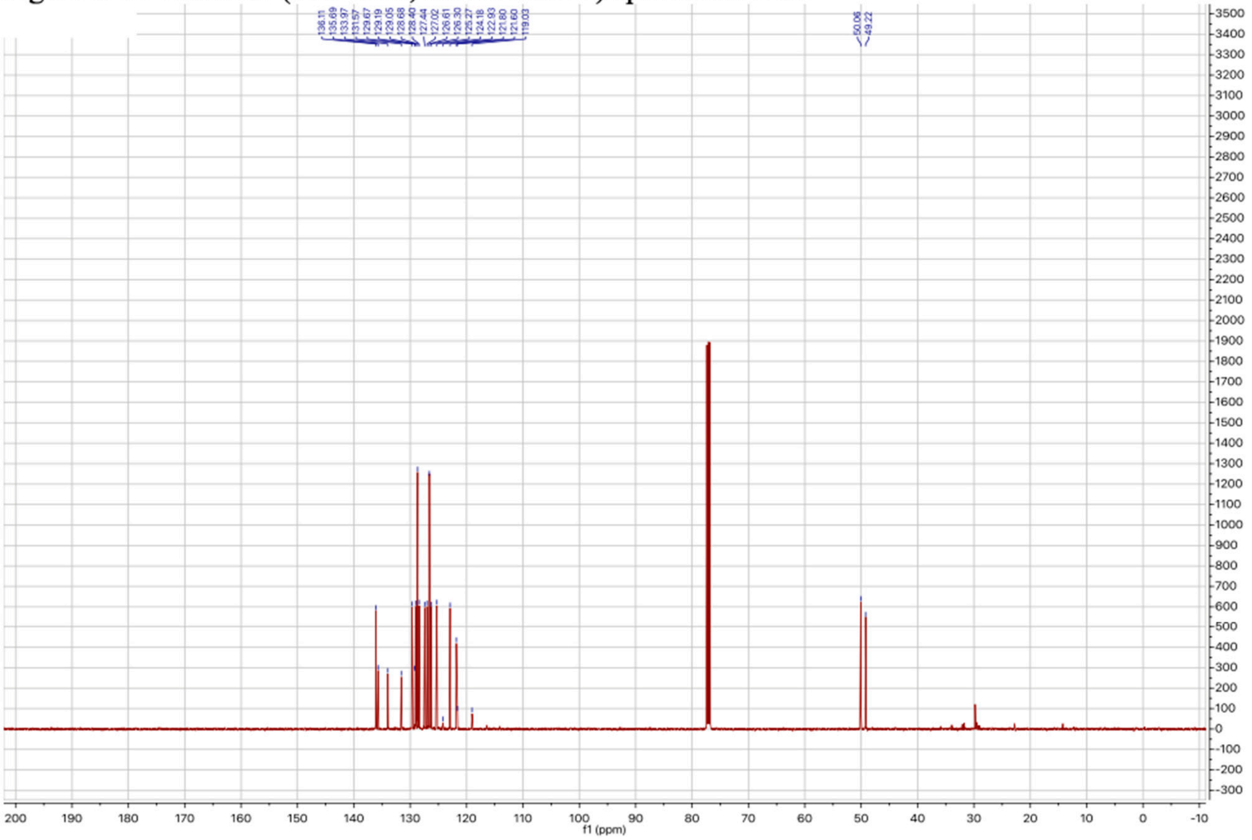

Figure S30: **^{13}C NMR** (126 MHz, $CDCl_3$) spectrum of **5k.**

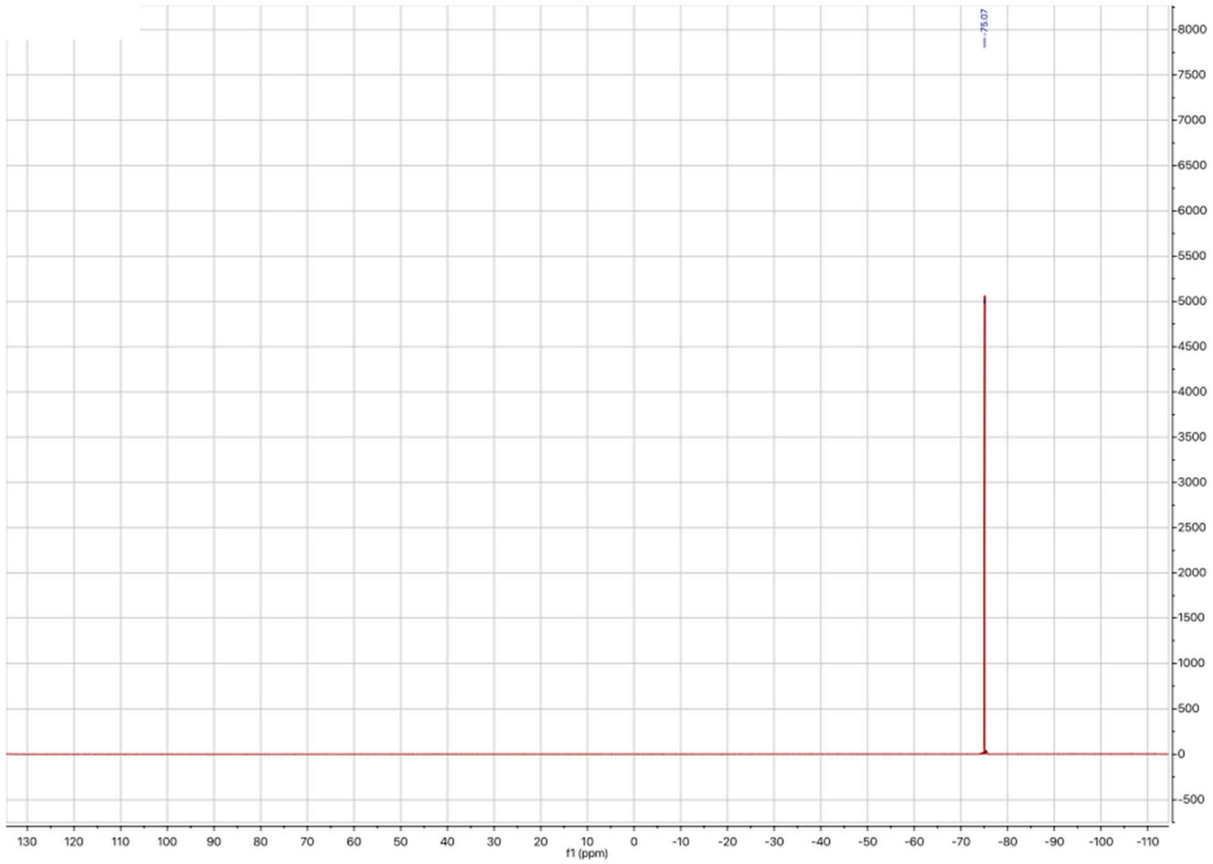

Figure S31: **^{19}F NMR** (471 MHz, $CDCl_3$) spectrum of **5k.**

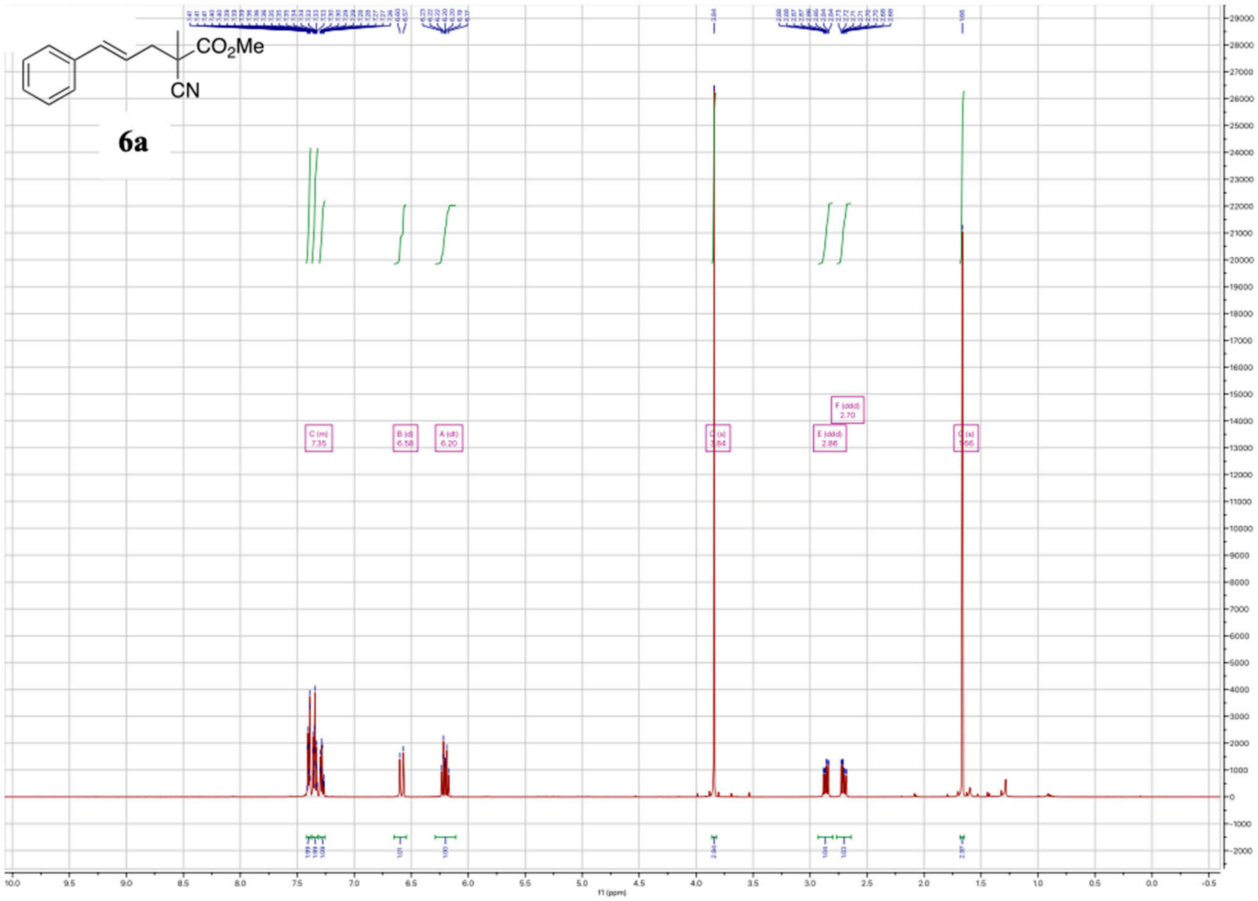

Figure S32: **^{1}H NMR** (500 MHz, Chloroform-*d*) spectrum of **6a.**

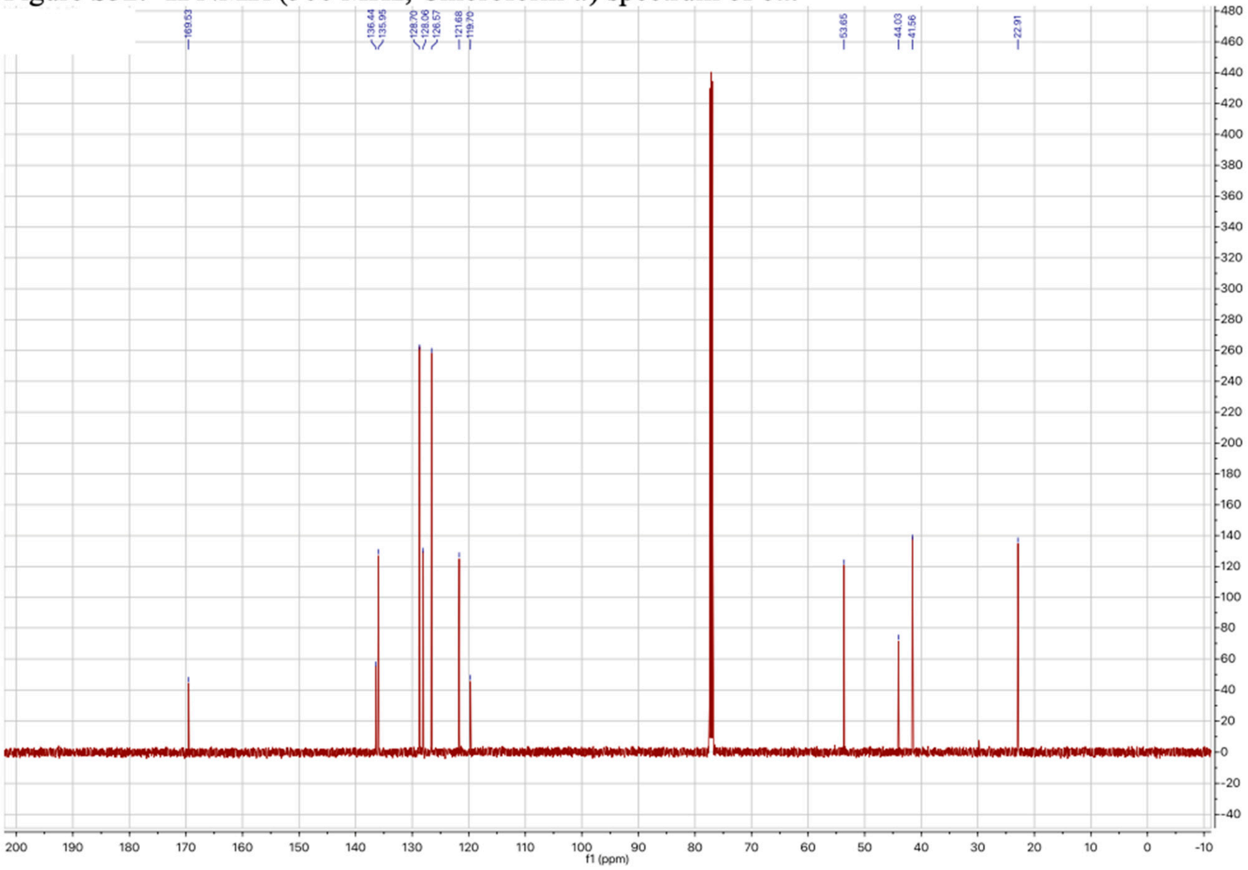

Figure S33: **^{13}C NMR** (126 MHz, $CDCl_3$) spectrum of **6a.**

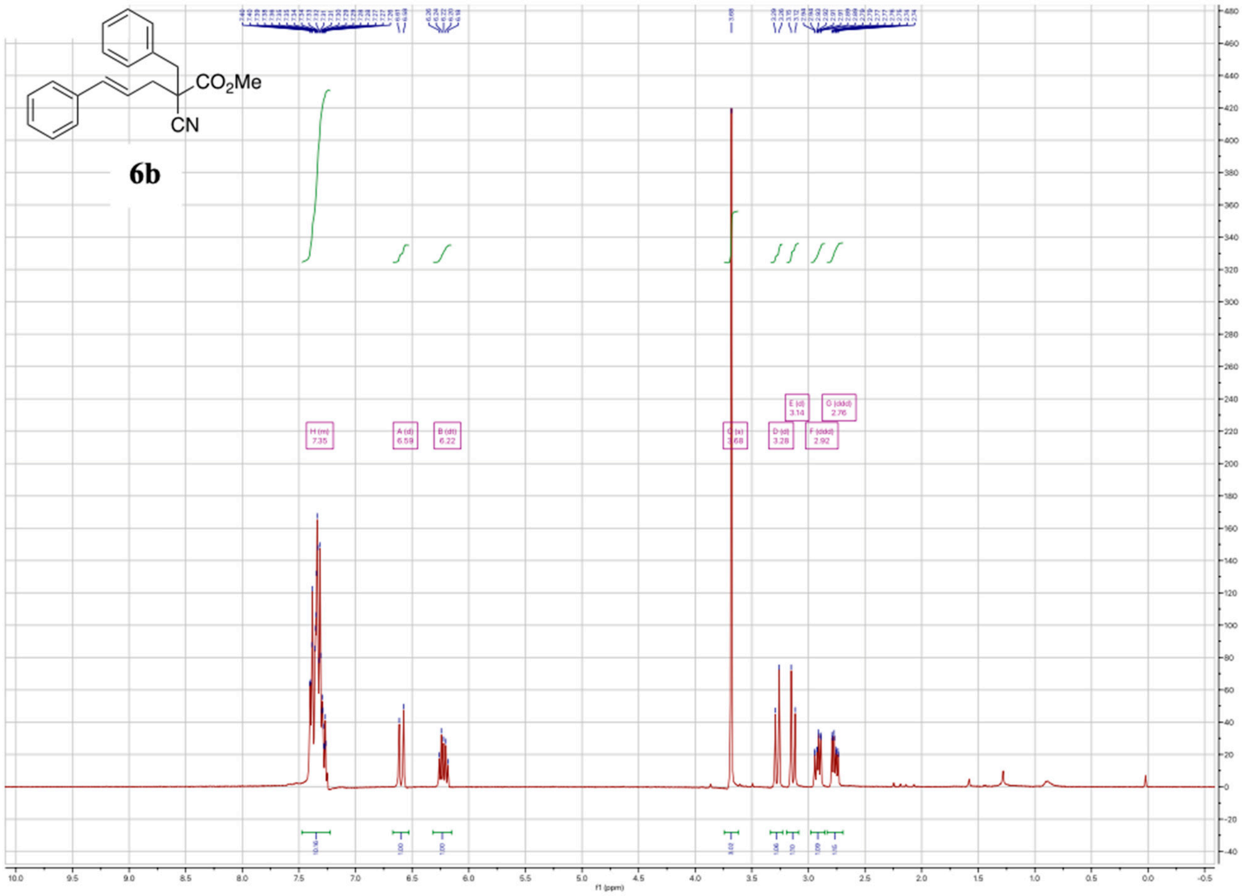

Figure S34: **^{1}H NMR** (400 MHz, Chloroform-*d*) spectrum of **6b.**

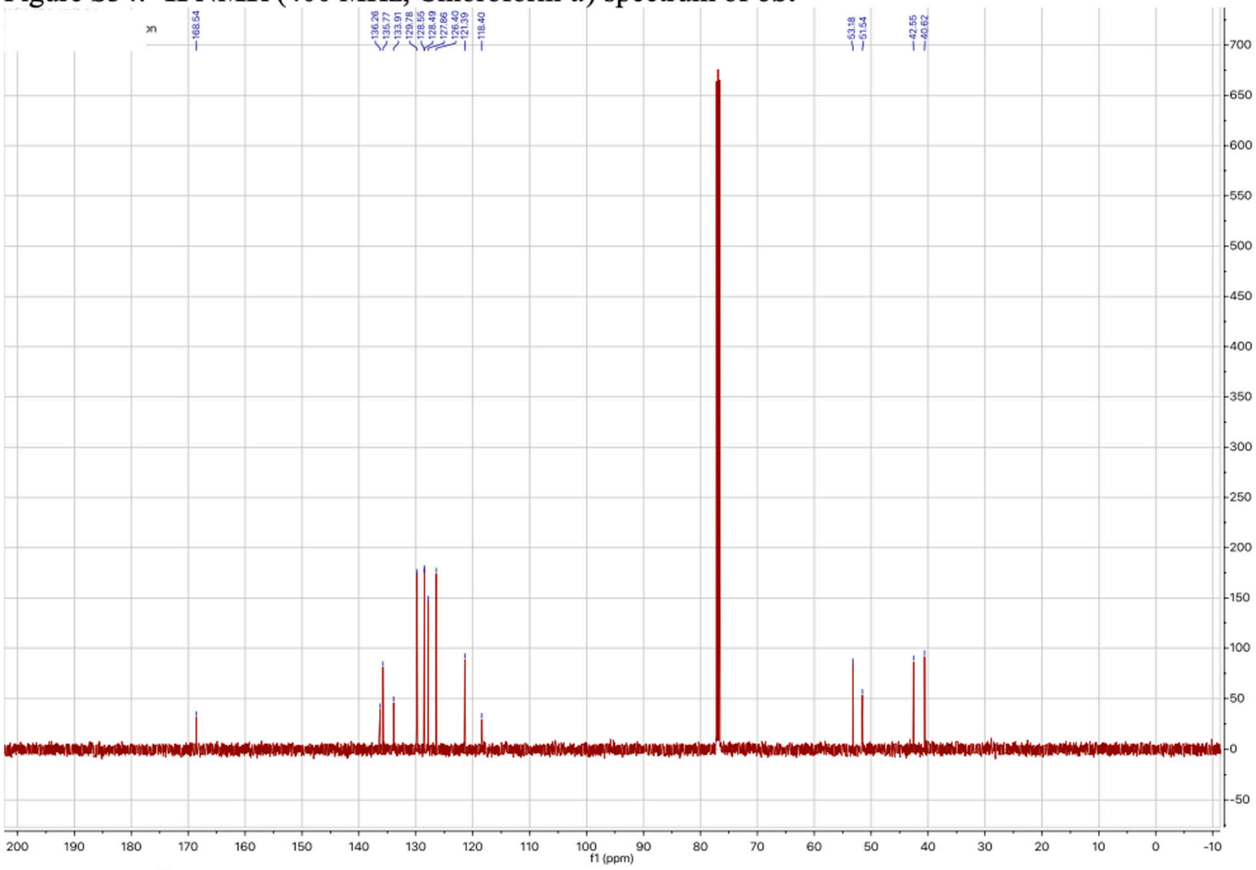

Figure S35: **^{13}C NMR** (126 MHz, $CDCl_3$) spectrum of **6b.**

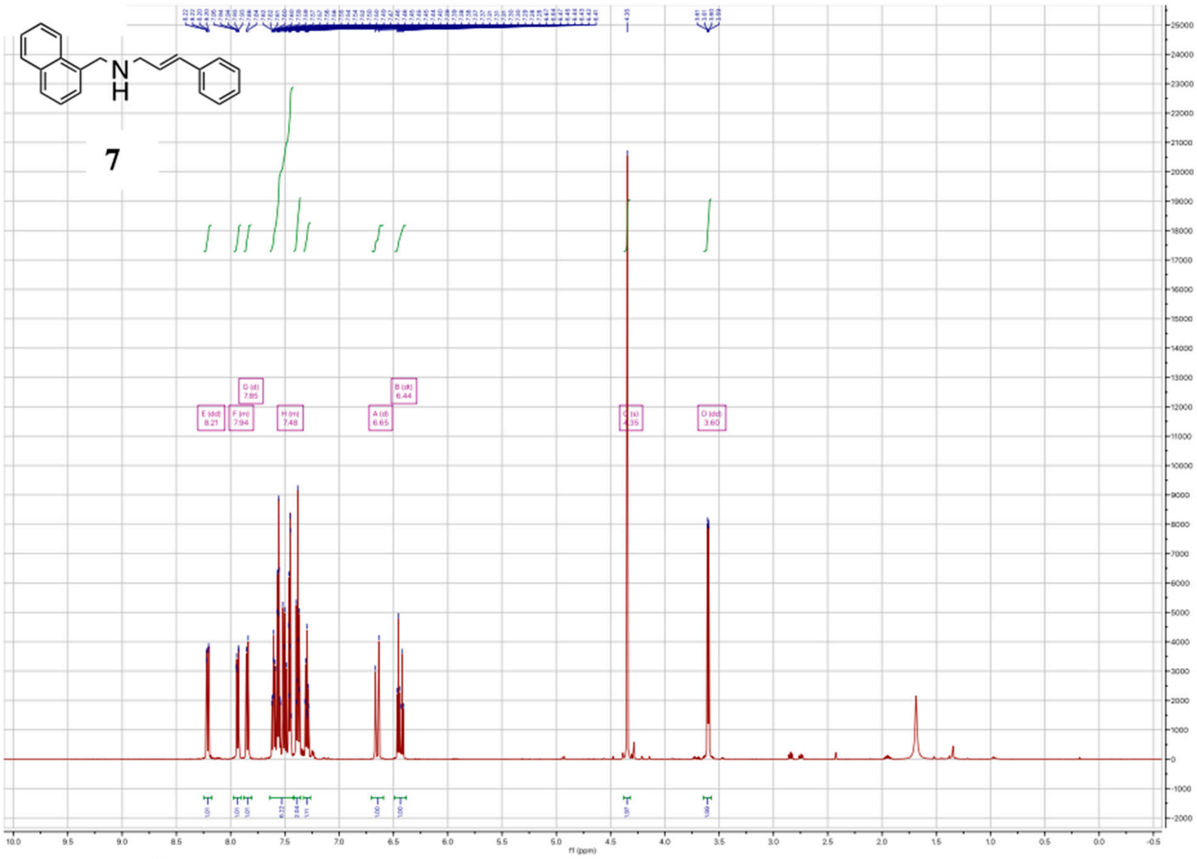

Figure S36: **^{1}H NMR** (500 MHz, Chloroform-*d*) spectrum of **7.**

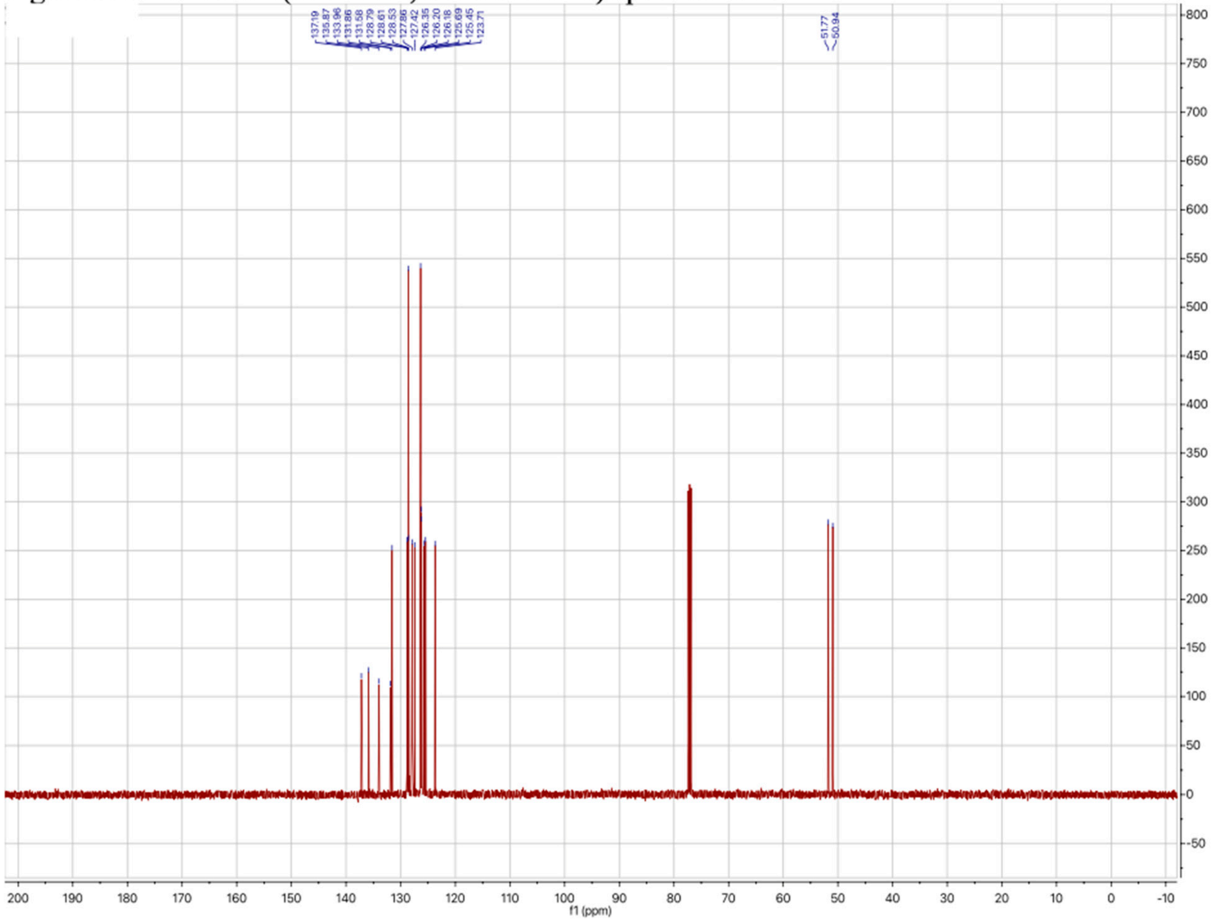

Figure S37: **^{13}C NMR** (126 MHz, $CDCl_3$) spectrum of **7.**

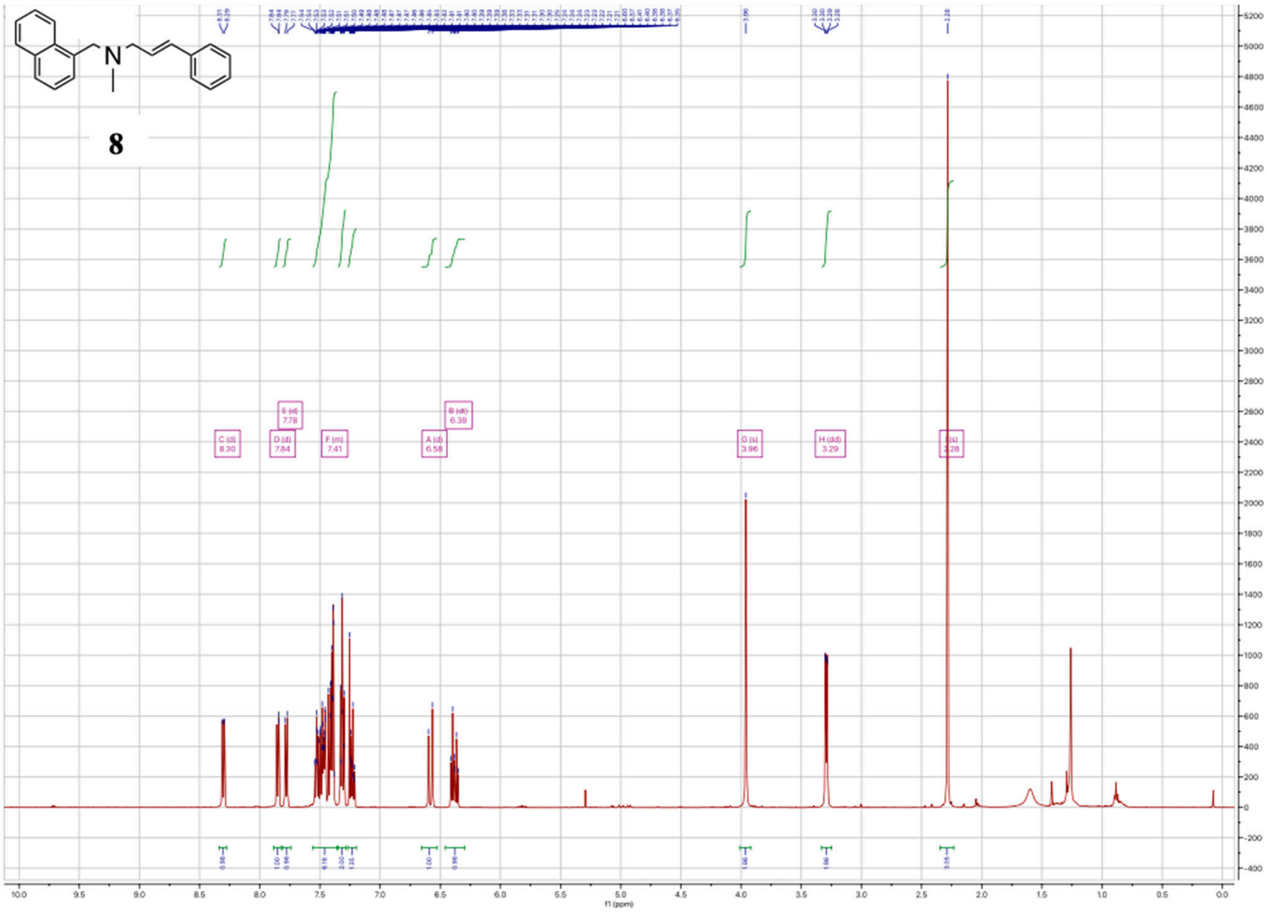

Figure S38: **^{1}H NMR** (500 MHz, Chloroform-*d*) spectrum of **8.**

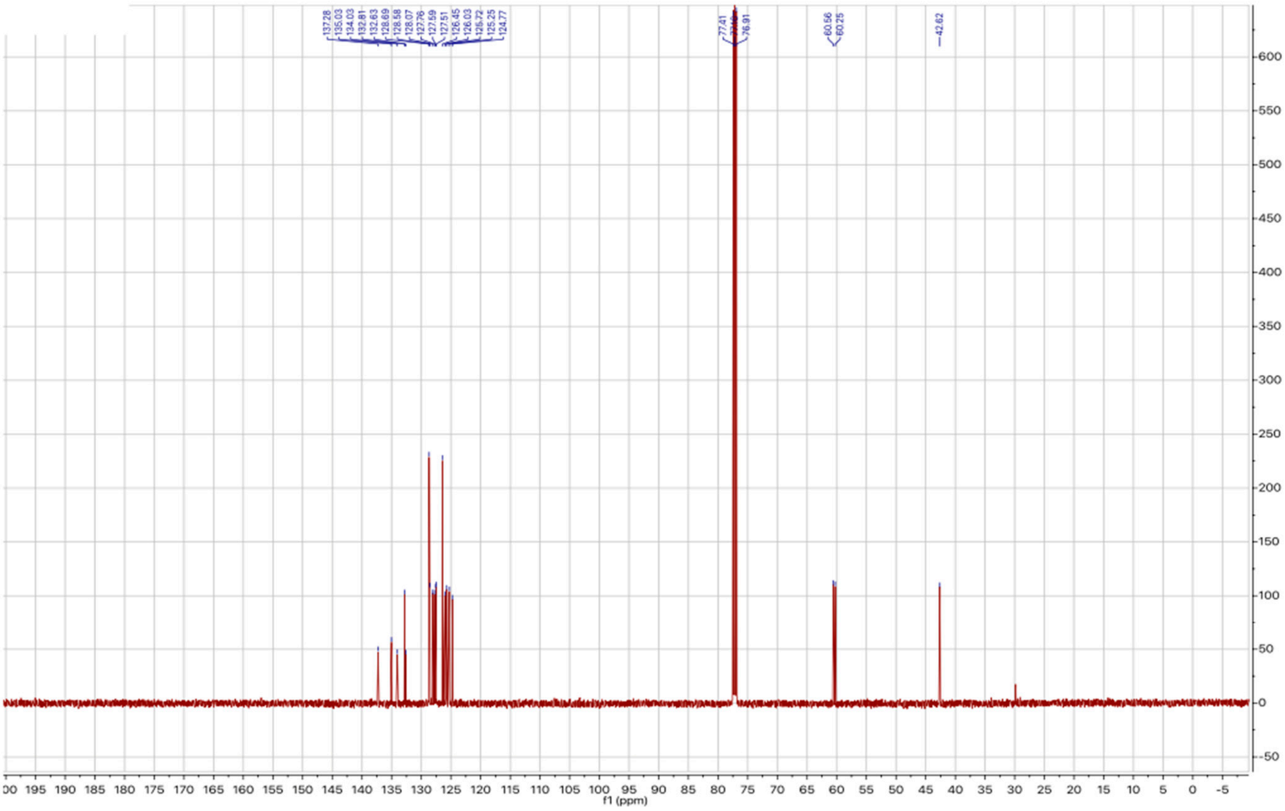

Figure S39: **^{13}C NMR** (126 MHz, $CDCl_3$) spectrum of **8.**

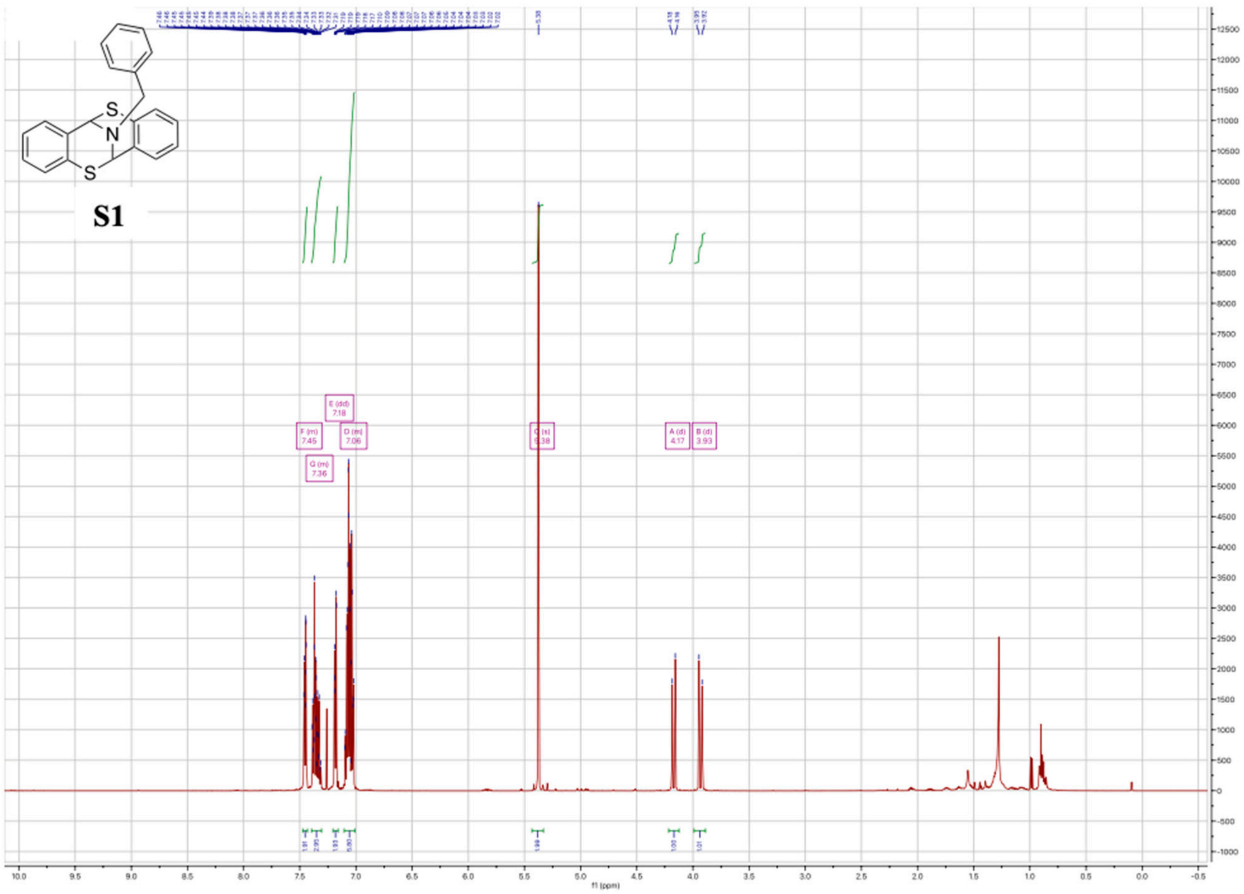

Figure S40: **^{1}H NMR** (500 MHz, Chloroform-*d*) spectrum of **S1.**

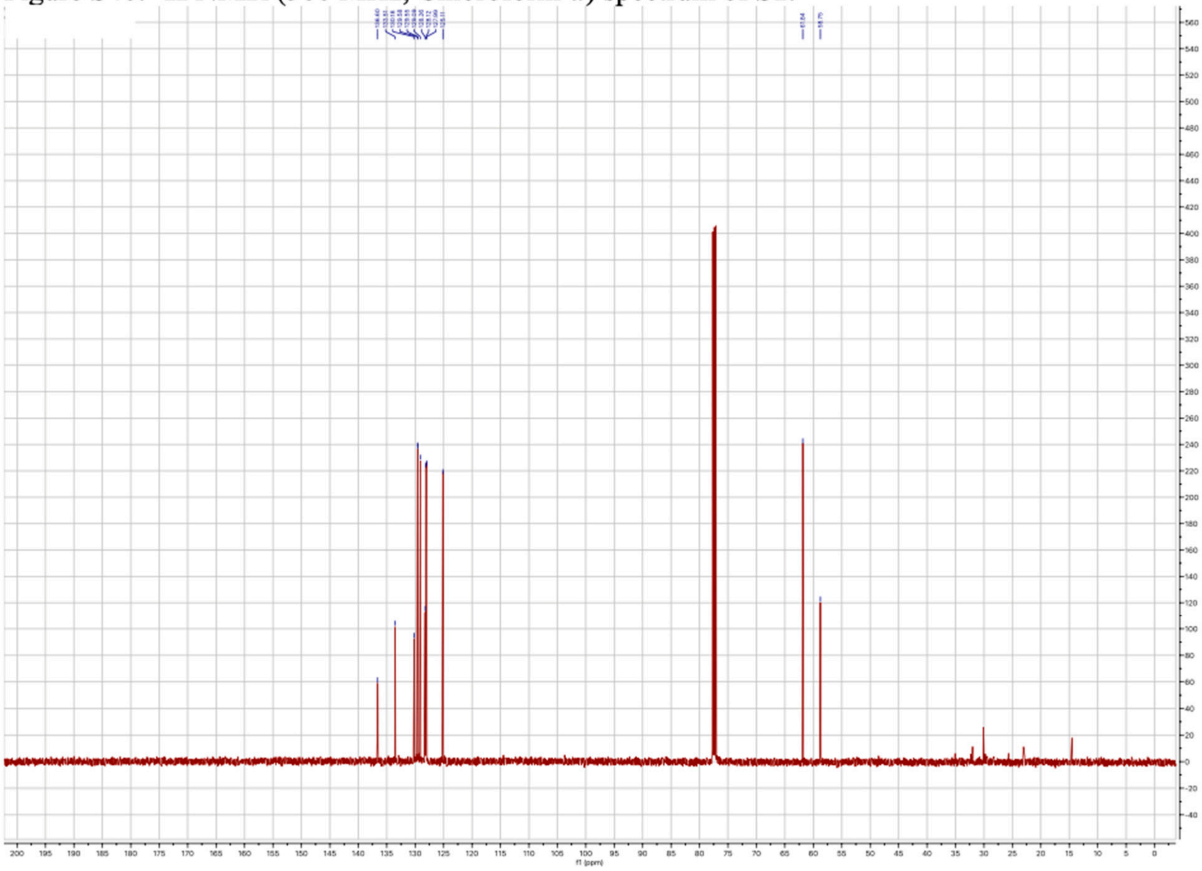

Figure S41: **^{13}C NMR** (126 MHz, $CDCl_3$) spectrum of **S1.**

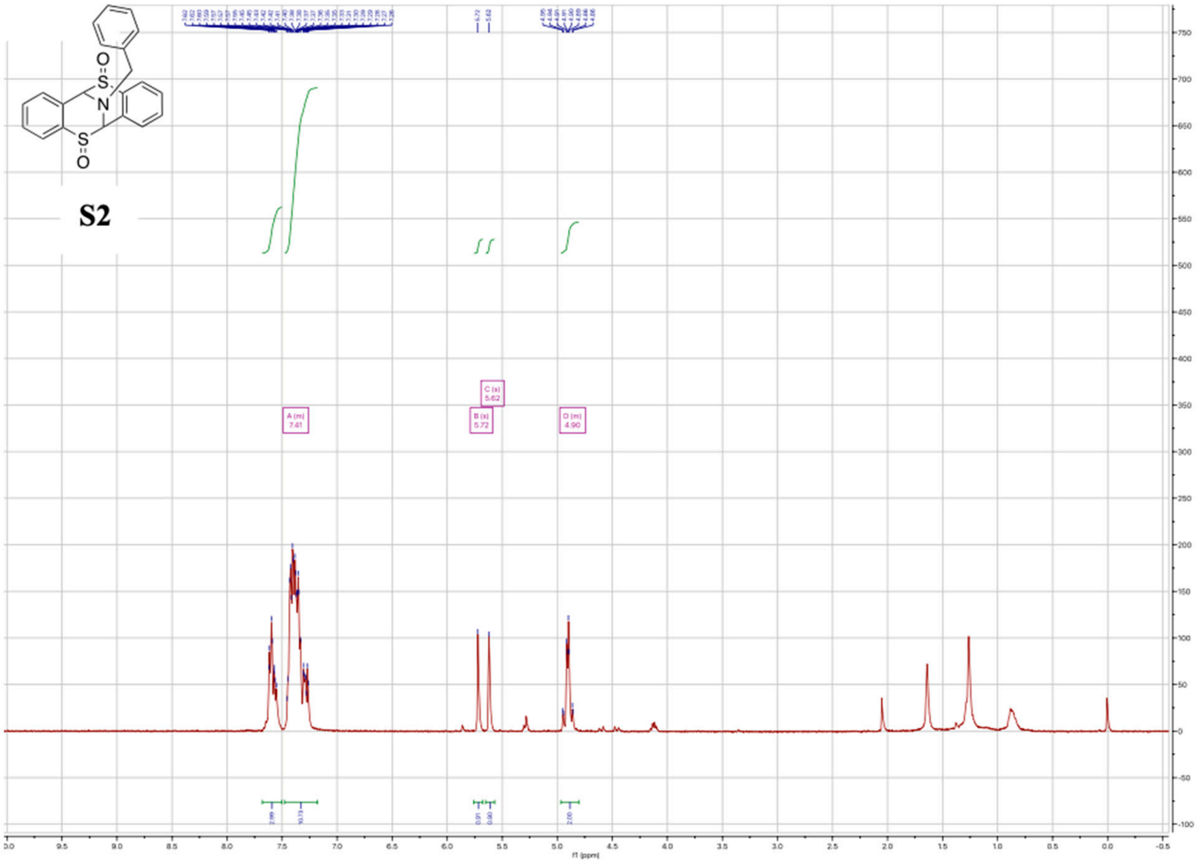

Figure S42: **^{1}H NMR** (400 MHz, Chloroform-*d*) spectrum of **S2.**

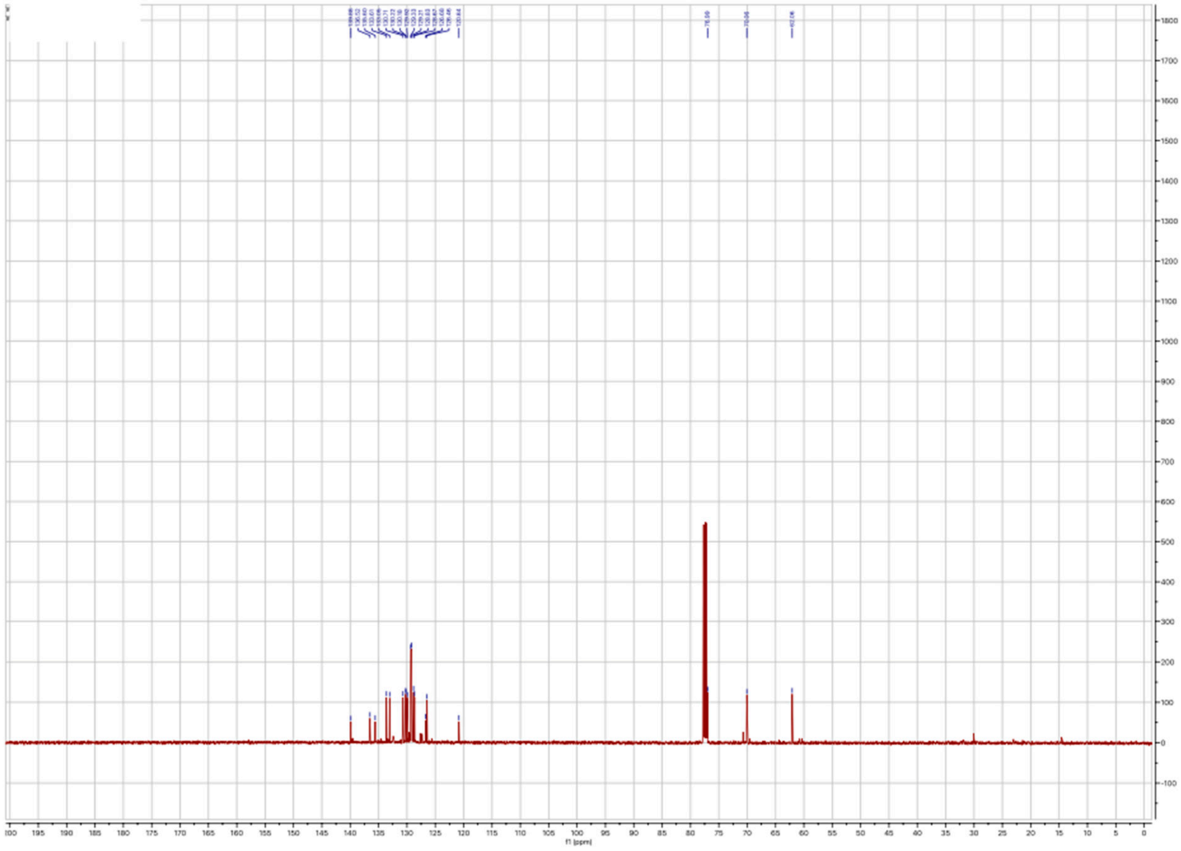

Figure S43: **^{13}C NMR** (126 MHz, $CDCl_3$) spectrum of **S2.**

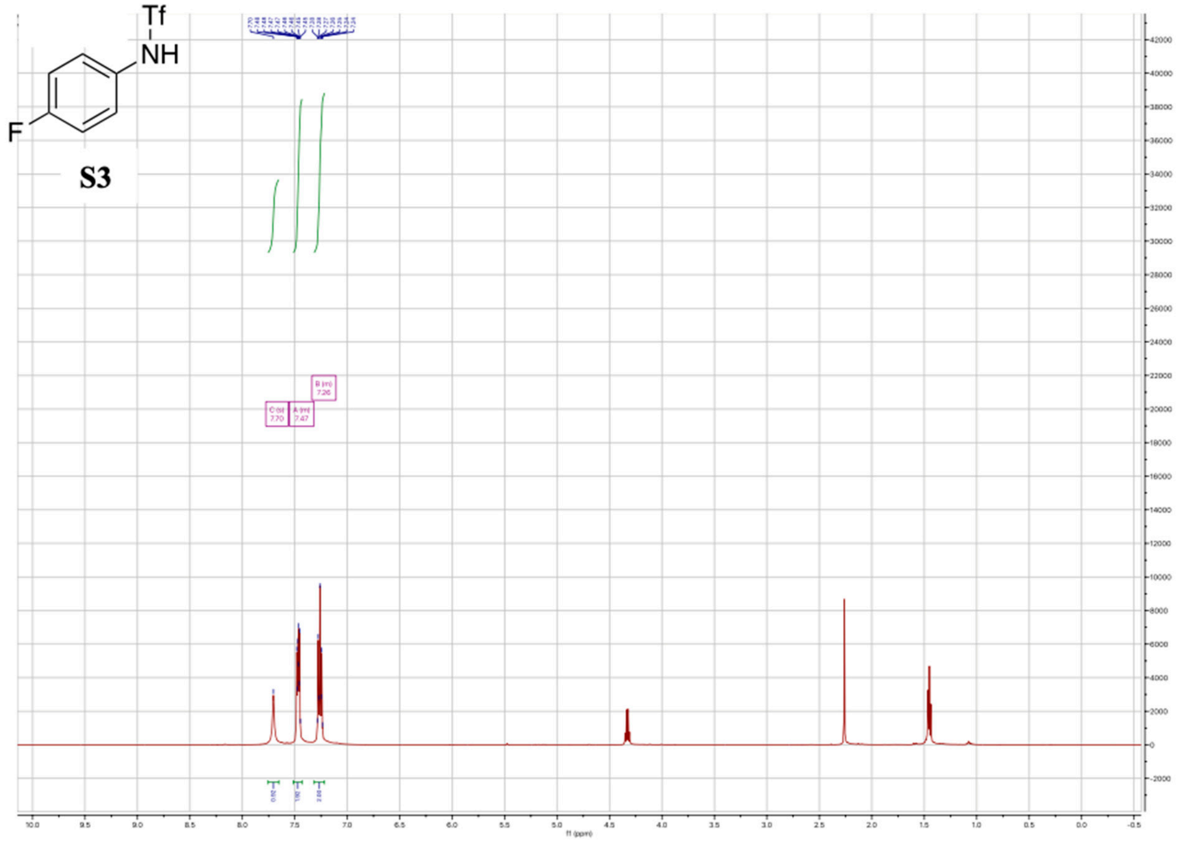

Figure S44: **¹H NMR** (500 MHz, Chloroform-*d*) spectrum of **S3.**

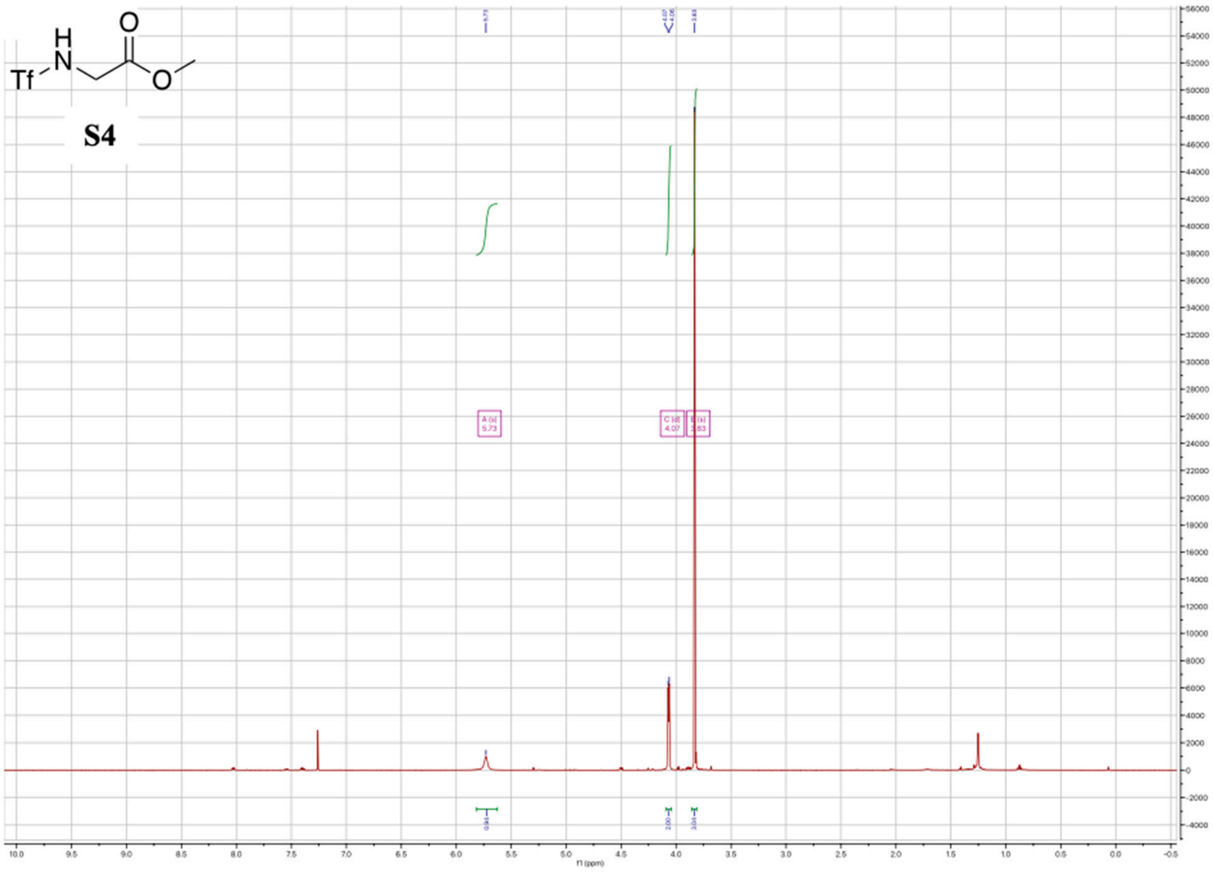

Figure S45: **¹H NMR** (500 MHz, Chloroform-*d*) spectrum of **S4.**

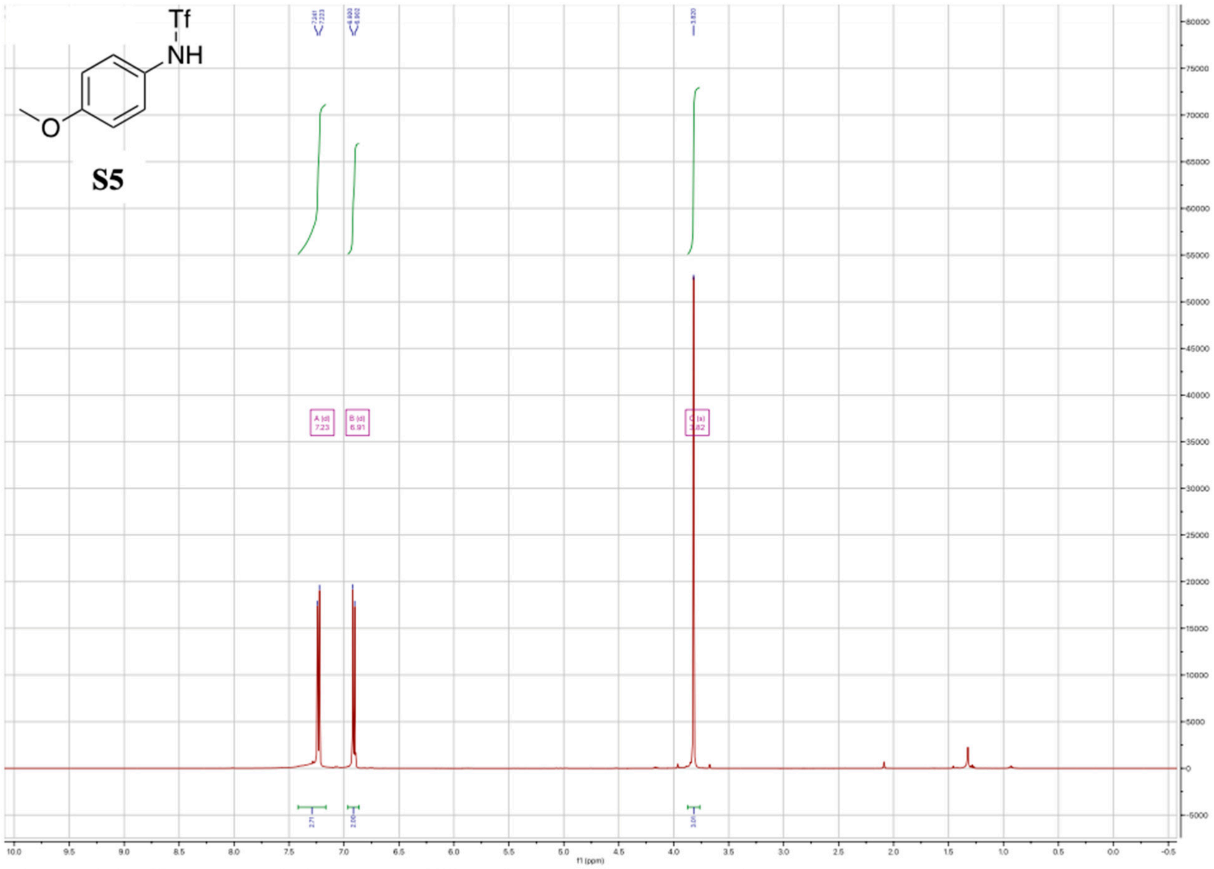

Figure S46: ^{1}H NMR (500 MHz, Chloroform-*d*) spectrum of **S5.**

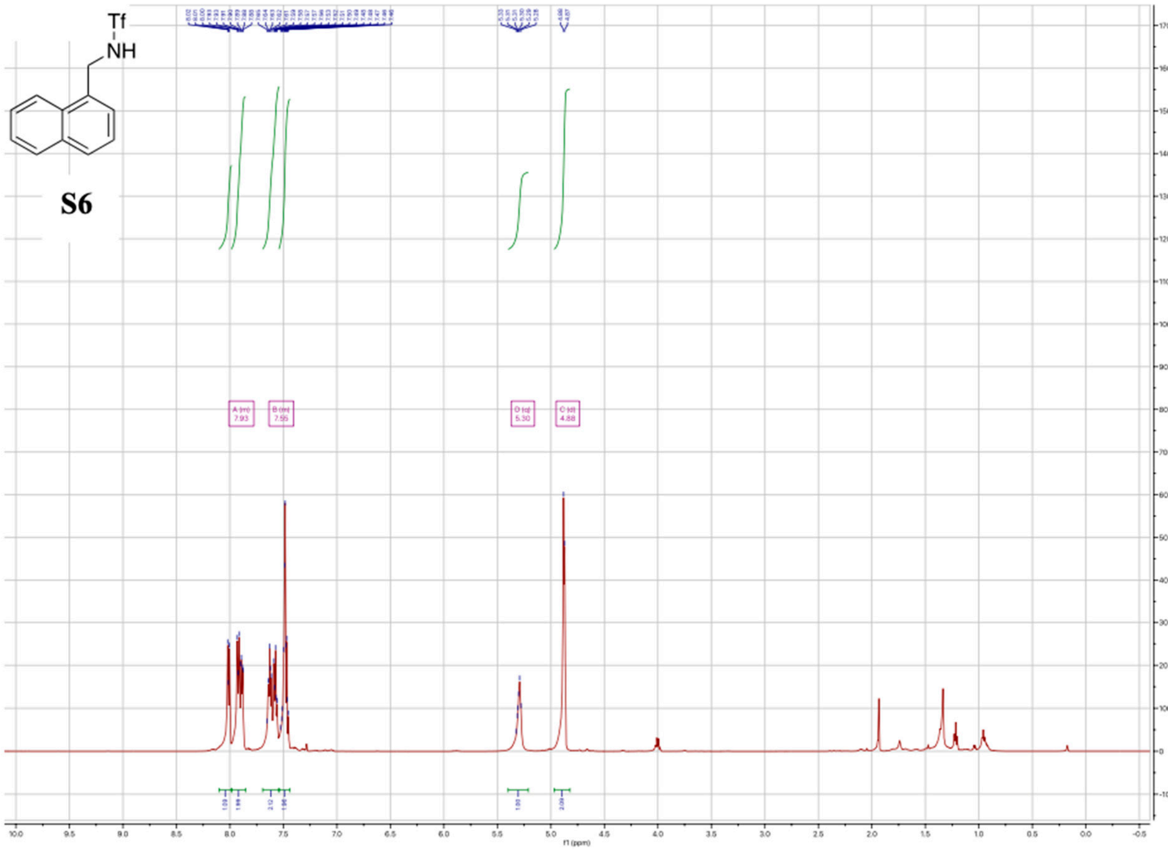

Figure S47: ^{1}H NMR (500 MHz, Chloroform-*d*) spectrum of **S6.**

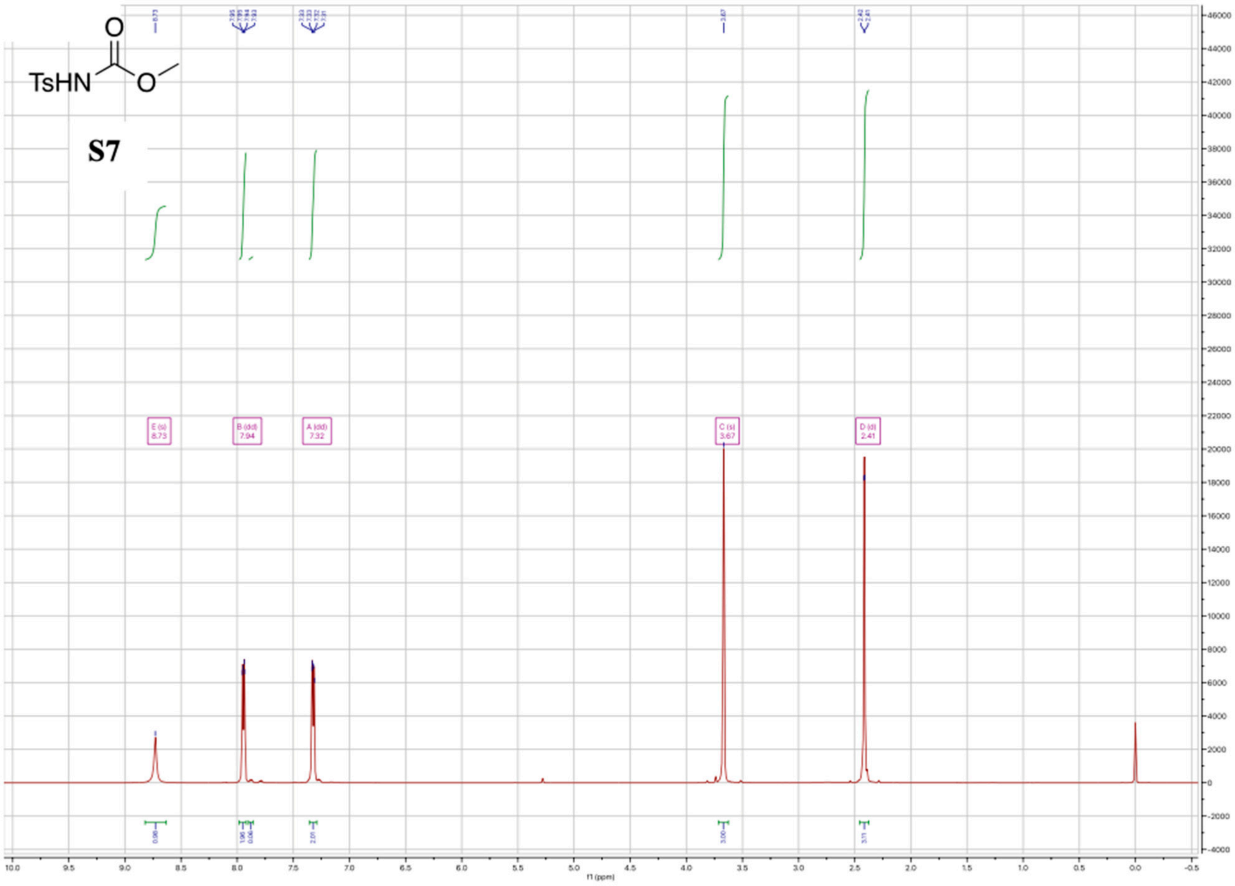

Figure S48: **^{1}H NMR** (500 MHz, Chloroform-*d*) spectrum of **S7.**

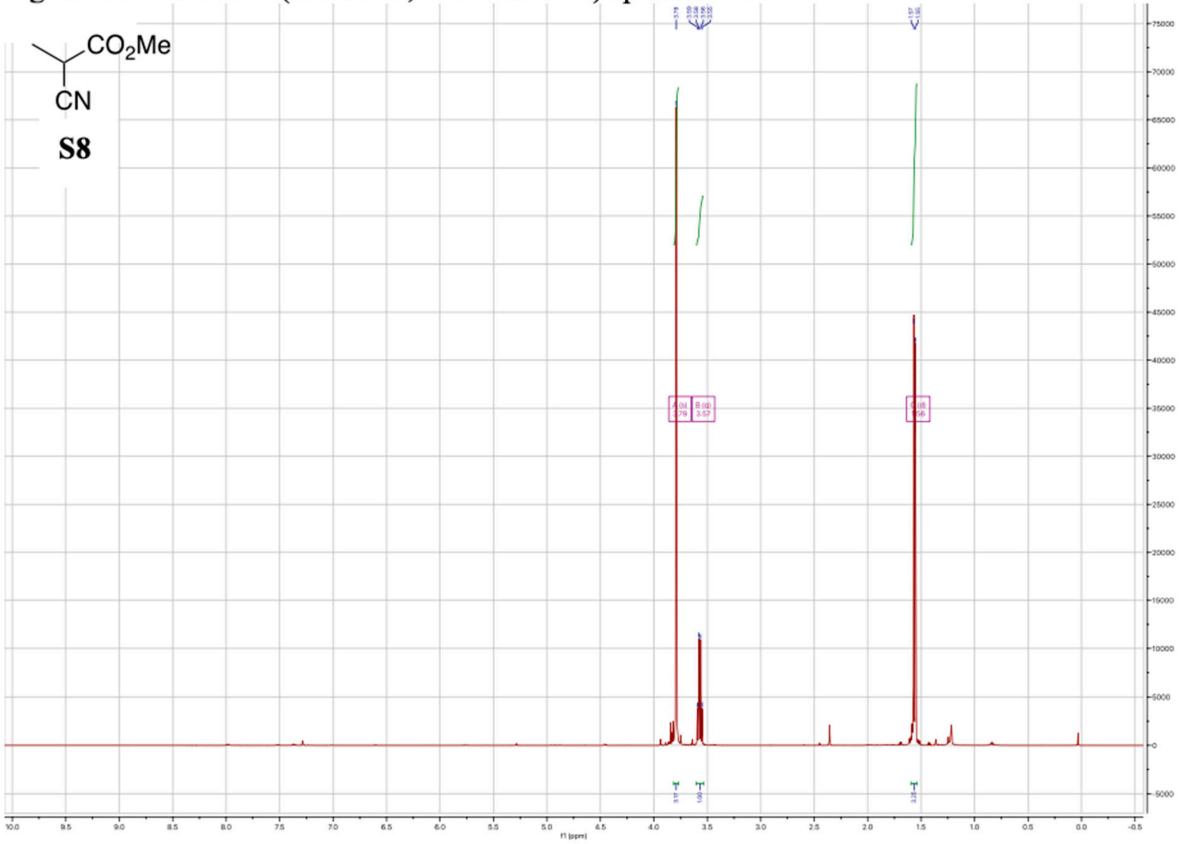

Figure S49: **^{1}H NMR** (500 MHz, Chloroform-*d*) spectrum of **S8.**

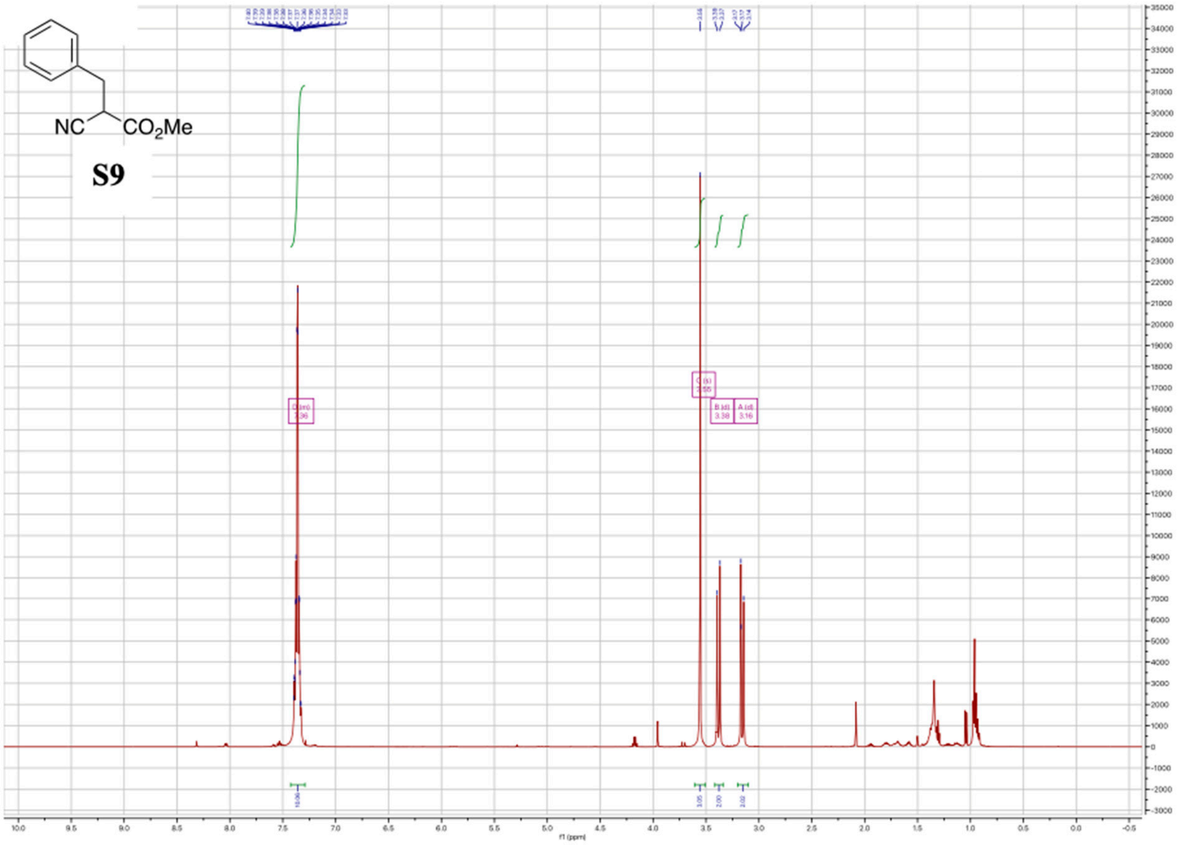

Figure S50: ^{1}**H NMR** (500 MHz, Chloroform-*d*) spectrum of **S9.**

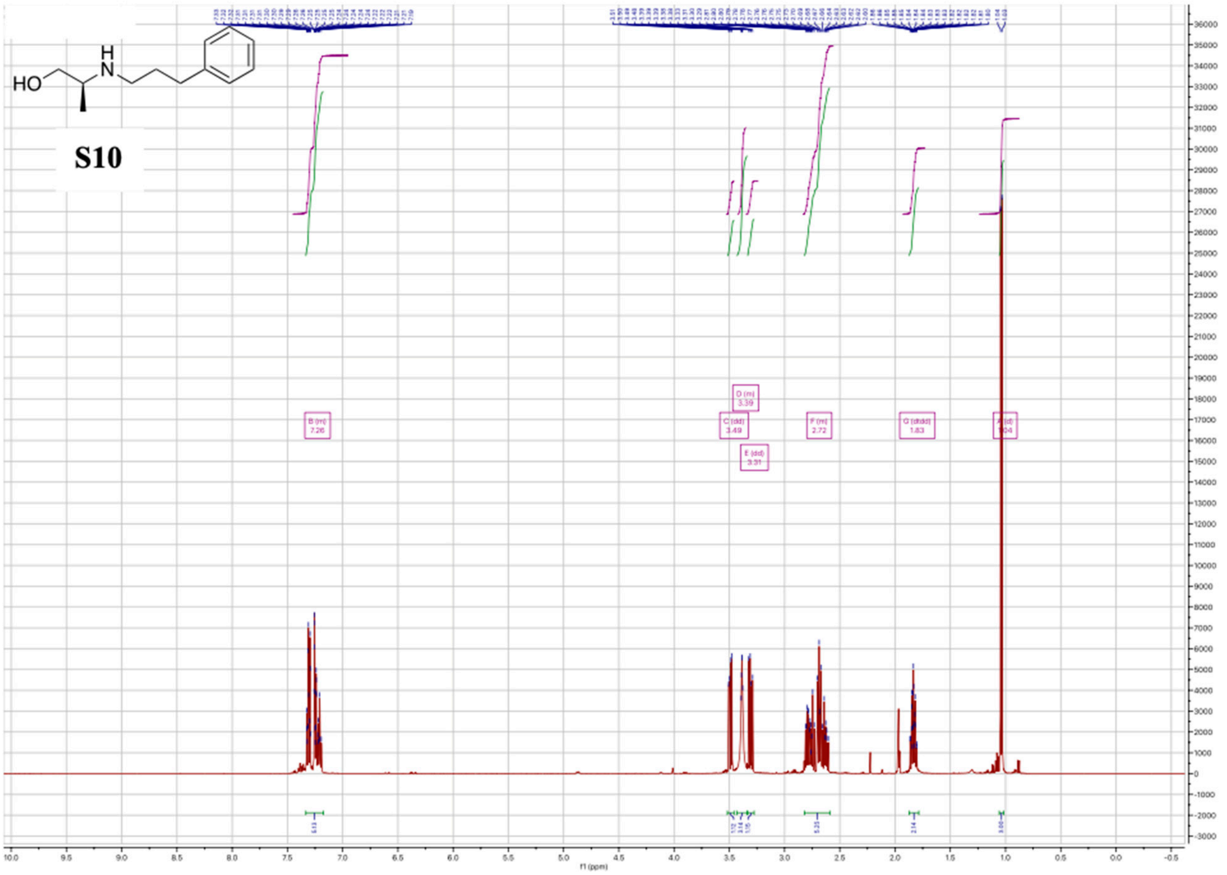

Figure S51: ^{1}**H NMR** (500 MHz, Acetonitrile-d_3) spectrum of **S10.**

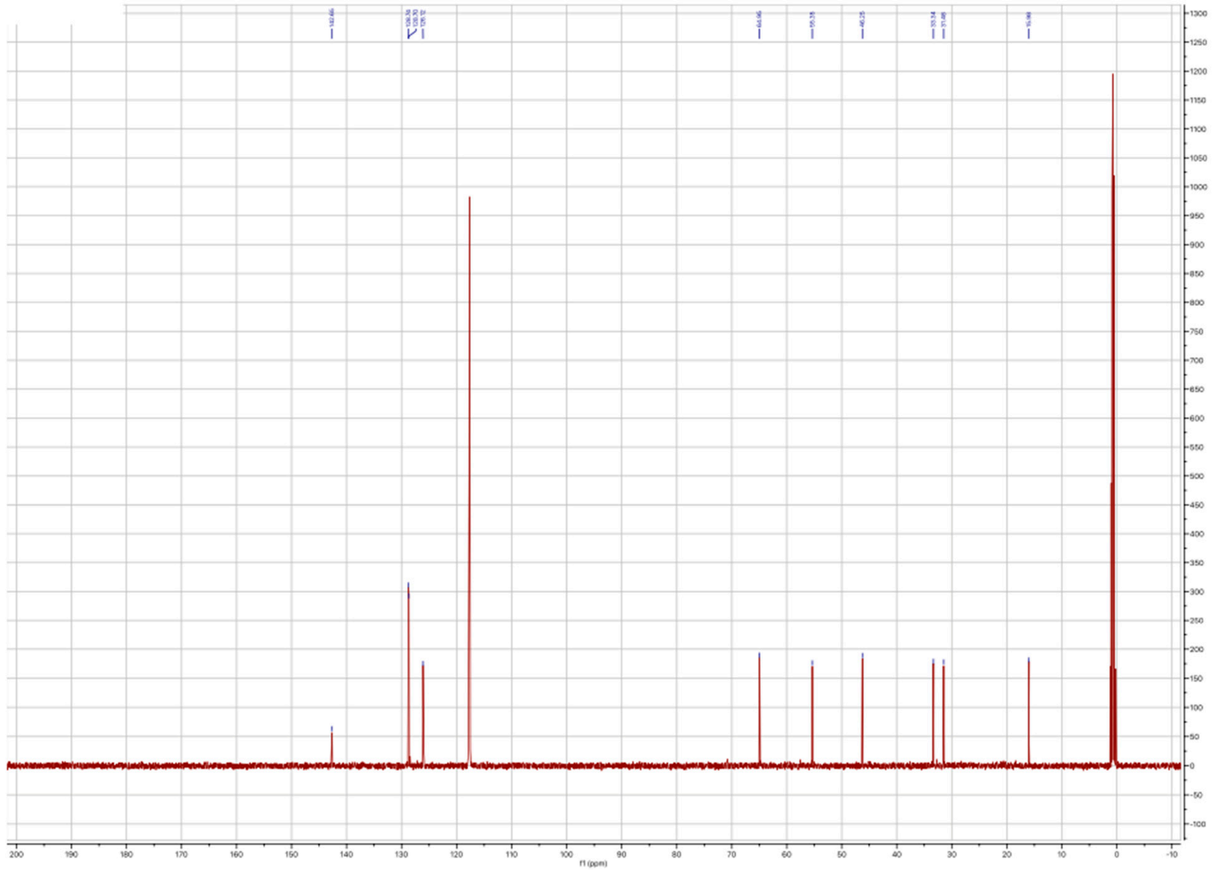

Figure S52: ^{13}**C NMR** (126 MHz, CD_3CN) spectrum of **S10.**

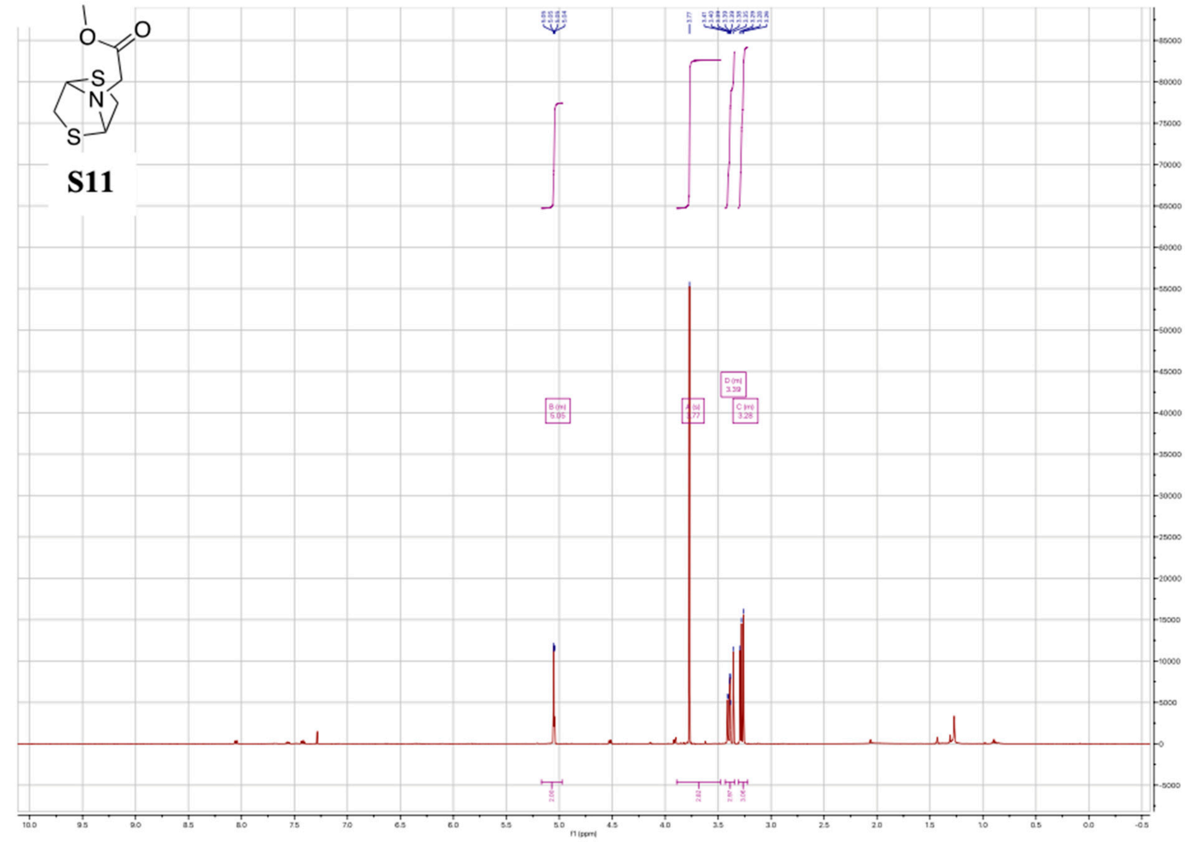

Figure S53: **^{1}H NMR** (500 MHz, Chloroform-*d*) spectrum of **S11.**

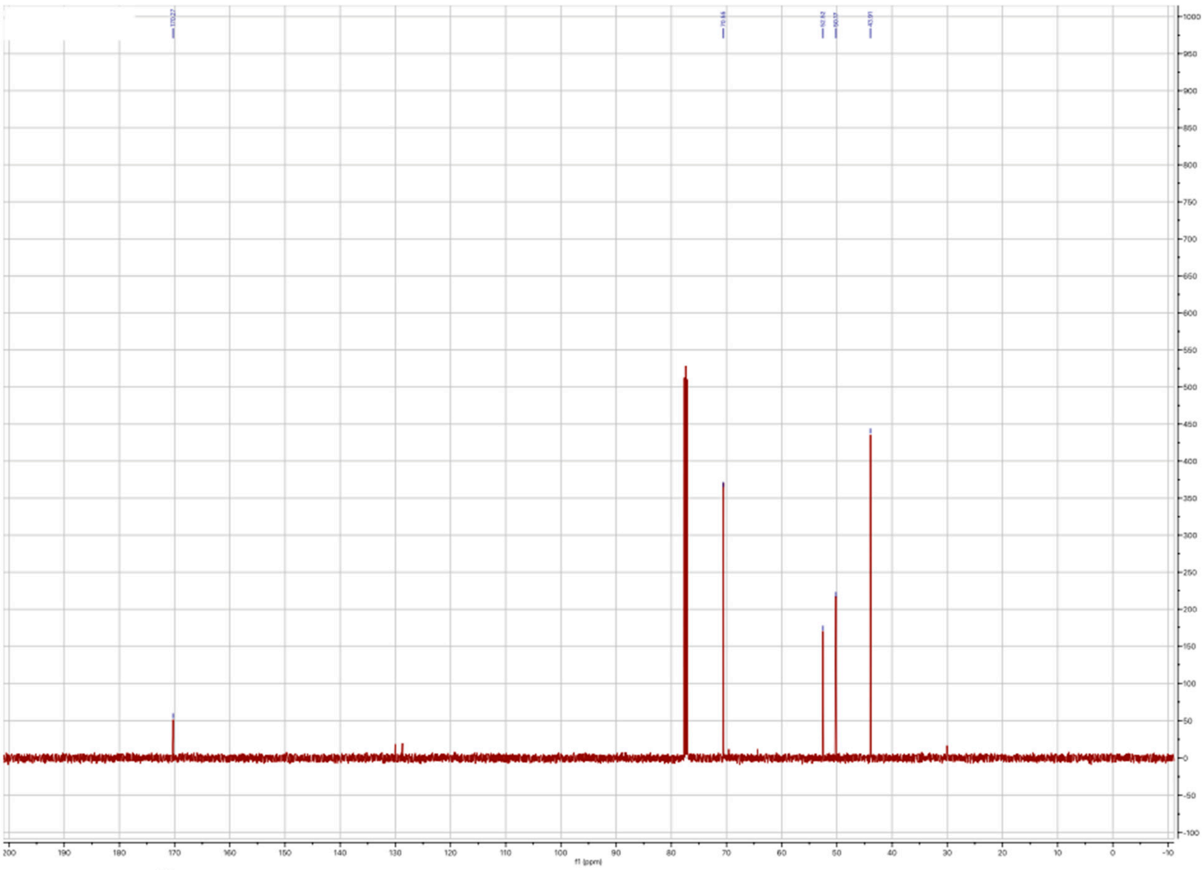

Figure S54: **^{13}C NMR** (126 MHz, $CDCl_3$) spectrum of **S11.**
